# Effects of common genetic variants associated with colorectal cancer risk on survival outcomes after diagnosis: A large population‐based cohort study

**DOI:** 10.1002/ijc.32550

**Published:** 2019-07-27

**Authors:** Yazhou He, Evropi Theodoratou, Xue Li, Farhat V.N. Din, Peter Vaughan‐Shaw, Victoria Svinti, Susan M. Farrington, Harry Campbell, Malcolm G. Dunlop, Maria Timofeeva

**Affiliations:** ^1^ Cancer Research UK Edinburgh Centre Medical Research Council Institute of Genetics & Molecular Medicine, Western General Hospital, The University of Edinburgh Edinburgh United Kingdom; ^2^ Colon Cancer Genetics Group, Medical Research Council Human Genetics Unit Medical Research Council Institute of Genetics & Molecular Medicine, Western General Hospital, The University of Edinburgh Edinburgh United Kingdom; ^3^ Centre for Global Health Research Usher Institute of Population Health Sciences and Informatics, The University of Edinburgh Edinburgh United Kingdom

**Keywords:** common genetic variants, colorectal cancer, survival, cohort study

## Abstract

Genome‐wide association studies have thus far identified 130 genetic variants linked to colorectal cancer (CRC) risk (*r* ^2^ < 0.2). Given their implication in disease causation, and thus plausible biologically effects on cancer‐relevant biological pathways, we investigated whether these variants are associated with CRC prognosis and also whether they might provide predictive value for survival outcome. We conducted the analysis in a well‐characterized population‐based study of 5,675 patients after CRC diagnosis in Scotland. None of the genetic risk variants were associated with either overall survival (OS) or CRC‐specific survival. Next, we combined the variants in a polygenic risk score, but again we observed no association between survival outcome and overall genetic susceptibility to CRC risk—as defined by common genetic variants (OS: hazard ratio = 1.00, 95% confidence interval = 0.96–1.05). Furthermore, we found no incremental increase in the discriminative performance when adding these genetic variants to the baseline CRC‐survival predictive model of age, sex and stage at diagnosis. Given that our study is well‐powered (>0.88) to detect effects on survival for 74% of the variants, we conclude that effects of common variants associated with CRC risk which have been identified to date are unlikely to have clinically relevant effect on survival outcomes for patients diagnosed with CRC.

## Abstract 2

**What's new?**

Genetic variants associated with colorectal cancer (CRC) risk potentially also exert effects on disease prognosis, though little is known about the heritability of CRC survival. In this population‐based study, genetic variants of CRC susceptibility were investigated for their relevance in CRC survival based on analyses of variants identified in genome‐wide association studies (GWASs). Nonsignificant associations were detected between small numbers of genetic variants and overall survival and CRC‐specific survival. Overall, the findings do not support the existence of prognostic effects of common CRC risk variants. Rather, CRC survival may have distinct genetic determinants, warranting separate investigation by GWAS.

**Abbreviations**
AJCC: American Joint Committee on Cancer
CI: confidence interval
CRC: colorectal cancer
FDR: false positive rates
HR: hazard ratio
LASSO: least absolute shrinkage and selection operator.
MAF: minor allele frequency
PRS: polygenic risk score
SCR: Scottish Cancer Registry
SOCCS: Study of Colorectal Cancer in Scotland

## Introduction

Globally, colorectal cancer (CRC) is the second leading cause of cancer‐related deaths, accounting for 9.2% of all cancer‐related deaths (0.8 million CRC deaths in 2018).^1^ The strongest known predictor of CRC outcome is stage, but even within one stage, there is considerable heterogeneity in survival. Identification of biomarkers of cancer prognosis can inform clinical management and treatment of disease. Evidence of the family concordance for CRC‐specific survival,^2^ together with some suggestions of improved survival in cancer patients with a family history compared to patients without a family history,^3^ indicates that genetic signature can affect prognosis of CRC patients after diagnosis. Indeed, improved survival for Lynch syndrome patients with germline rare variations in DNA mismatch repair genes is well documented,^4^ suggesting that genetic variants associated with CRC pathogenesis may subsequently affect tumor progression. However, very few studies with sufficient power tested roles of common genetic risk variants in CRC prognosis. Previously published smaller studies examined up to 30 CRC risk genetic loci and detected no or little evidence of associations with survival.^5^, ^6^, ^7^ Two recent large meta‐analyses of genome‐wide association studies (GWAS)^8^, ^9^ have identified more than 70 new genetic variants associated with CRC risk. In this analysis, we investigated the association between all previously and newly GWAS‐identified common genetic variants and CRC survival.

## Materials and Methods

We included 5,675 CRC cases (detailed patient selection in Supporting Information Fig. S1) with genome‐wide genotyping data and data on age at diagnosis, sex and American Joint Committee on Cancer (AJCC) stage information from a population‐based case–control study (Study of Colorectal Cancer in Scotland, SOCCS; 1999‐current).^10^ Ethics approval was obtained from the MultiCentre Research Ethics committee for Scotland (approval number MREC/01/0/5) and other committees (presented elsewhere^10^). A total of 130 genetic variants identified by previous GWAS studies^8^, ^9^ were genotyped or imputed (25/130 variants were directly genotyped). For correlated variants (linkage disequilibrium *r* ^2^ > 0.2), we selected ones with smaller *p* values in the association with CRC risk. Genotyping was conducted using the Illumina HumanHap300, HumanHap240S and OmniExpressExome BeadChip 8v1 arrays. Standard quality control measures were applied as described previously.^9^, ^11^, ^12^ Untyped variants were imputed using SHAPEIT v2^13^ and IMPUTEv2^14^ softwares based on a merged reference panel comprising of 1,000 Genomes Project (phase 1, December 2013 release) and UK10K (April 2014 release) samples. We excluded poorly imputed variants (information measure <0.80) and rare variants (minor allele frequency [MAF] <0.05%) as presented in the previous publication.^9^ Death registration and cause of death was ascertained from the Scottish Cancer Registry (SCR), and patients were prospectively followed up until death or July 1, 2017 (censored date), whichever came first. The survival outcomes included overall survival (OS) and CRC specific survival (CSS). The criteria of assigning cause of death can be found elsewhere.^15^ In order to measure the overall genetic CRC susceptibility, we created polygenic risk scores (PRS) including all the 130 variants based on the number of CRC risk alleles carried by each patient. We employed Cox proportional hazards models to investigate effects of individual variant (additive model) and the PRS on survival outcomes adjusting for age at diagnosis, gender and AJCC stage. Bonferroni correction for multiple testing was adopted and *p* < 0.0005 was considered statistical significance. We also used the false positive rates (FDR) approach (*p* < 0.05 was the significance threshold after correction) as a sensitivity analysis.^16^ A summary of the 130 included genetic variants is presented in Supporting Information Table S1. Using the method provided by Owzar *et al*.,^17^ we calculated the power of variants with various minor allele frequency (MAF) on a range of effects. Stratified analyses were also performed by sex, stage and tumor site. To further explore the potential predictive value of these variants, we applied a least absolute shrinkage and selection operator (LASSO) regression model with 10‐fold cross‐validation in 70% randomly selected patients (training set) to select predictors.^18^ Harrell's concordance indices (C statistic) were calculated to measure the discriminative ability of selected predictors in the remaining 30% patients (test set) and a U‐statistic test was adopted to determine the probability that added genetic variants could increase the model concordance.^19^

We also compared hazard ratio (HR) for OS and CSS with the risk results from a recently published meta‐analysis of genome‐wide association studies of CRC risk.^9^ We excluded ambiguous AT and CG variants (*n* = 6, rs10161980, rs2186607, rs2696839, rs2732875, rs61336918, rs7398375) from the analysis to avoid bias due to strand differences between studies. For the variants included in the analysis (*n* = 124), we aligned alleles and effect estimates between GWAS on risk and SOCCS survival analysis. We hypothesized that in case of no effects, the CRC risk variants will cause improvement or impairment of OS and CSS in equal proportion (62 variants with effects on risk and survival going in the same direction *vs*. 62 variants with effects on risk and survival going in opposite directions). We further counted all instances of results with similar directions of effects such a HR and OR above 1 or below 1 and compared it to the expected distribution using exact binomial test. Proportions of risk variants associated with worse OS and CSS and corresponding 95% confidence intervals (CIs) were calculated.

All genetic variants were annotated using (*i*) association with the *cis* gene expression in colon transverse tissue (*n* = 246) from the Genotype‐Tissue Expression (GTEx) database,^20^ (*ii*) presence of known and predicted regulatory elements in RegulomeDB database^21^ and (*iii*) predicted effect on the structure and function of a protein as implemented in SIFT^22^ and PolyPhen‐2.^23^

## Results and Discussion

**Figure 1:**
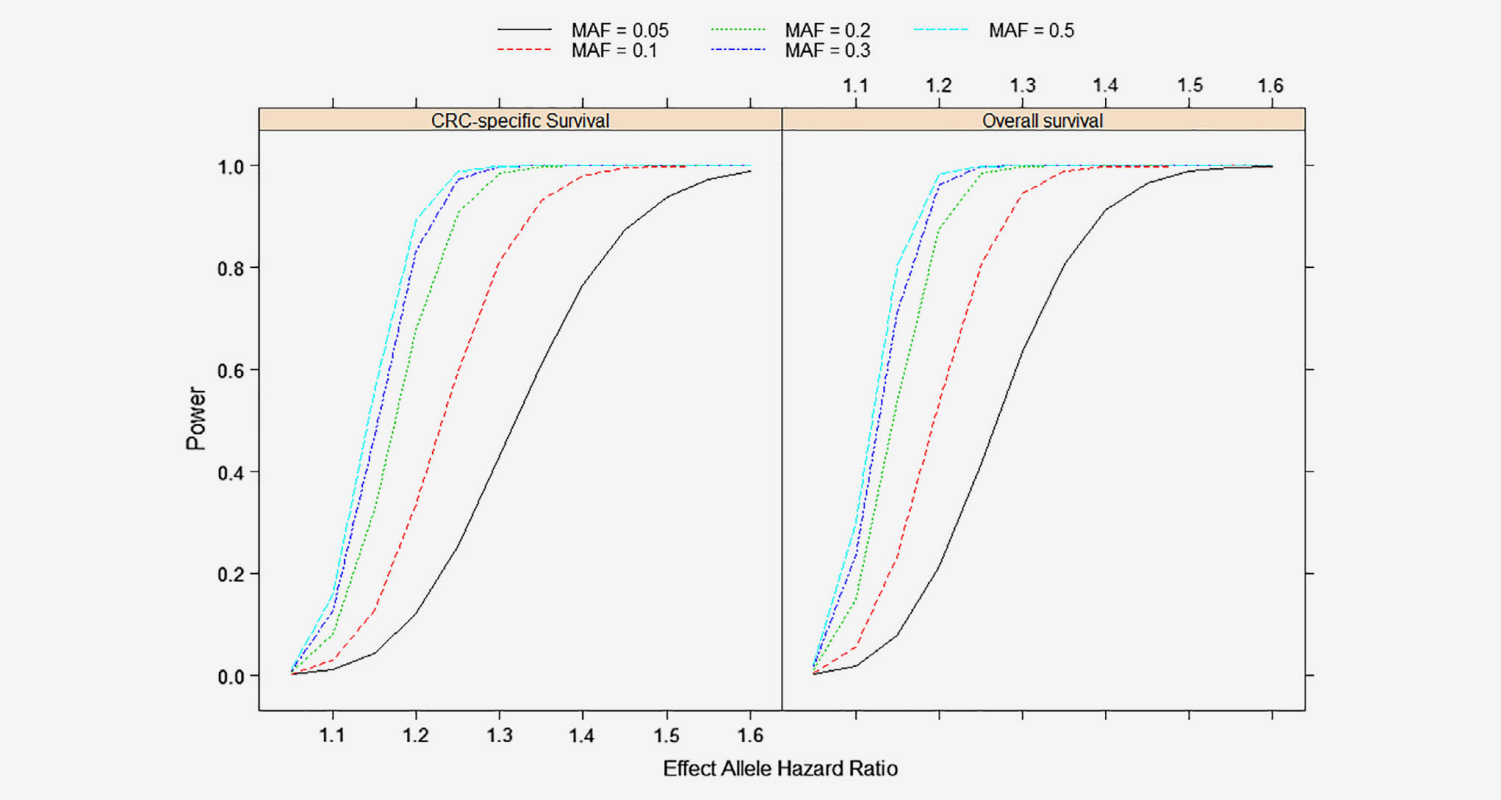
Power curve for overall and CRC‐specific survival using additive model. Abbreviations: CRC, colorectal cancer; MAF, minor allele frequency. [Color figure can be viewed at wileyonlinelibrary.com]

The basic characteristics of included CRC patients are summarized in Table 1. In total, 1,918 patients (34%) died during follow‐up. With 5,675 CRC cases, our study had 88% power to detect a hazard ratio of 1.20 on OS for 97/130 (74%) variants (MAF > 0.15) at the significance level of 0.0005 (a power curve for other effects is shown in Fig. 1). Overall, we observed 14 genetic variants associated with OS and 10 with CSS at nominal statistical significance (*p* < 0.05) with six variants (rs10994860, rs12143541, rs3217810, rs34405347, rs6065668, rs847208) being associated with both OS and CSS. However, none of the variants remained statistically significant after Bonferroni or FDR correction. The summary results for variants with nominal significance are presented in Table 2. Stratified analyses by stage, sex and tumor site did not identify any statistically significant associations after multiple‐testing correction either (Supporting Information Tables S2–S4). With regard to overall genetic susceptibility to CRC, no statistically significant association was observed between the PRS and OS or CSS (Table 2). The LASSO regression model selected six variants for OS in addition to age at diagnosis, sex and AJCC stage to minimize prediction error in the train set. However, nearly no incremental predictive improvement was observed compared to the model without genetic variants in the test set (C‐statistic for OS: 0.73282 *vs*. 0.73277, U‐statistic test: *p* = 0.322). Similar results were found for CSS (Supporting information Table S5).

**Table 1:** Basic characteristics of included CRC cases

Characteristics	SOCCS CRC cases (*n* = 5,675)
Age at diagnosis (years)^1^	64.5 (54.6–71.6)
Sex	
Male	3,235 (57.0%)
Female	2,440 (43.0%)
AJCC stage	
I	1,005 (17.7%)
II	1,891 (33.3%)
III	1,995 (35.2%)
IV	784 (13.8%)
Site	
Colon	3,392 (59.8%)
Rectum	2,201 (38.8%)
Colon and rectum	16 (0.3%)
Unknown	66 (1.2%)
Follow‐up time (years)^1^	5.09 (2.43–11.42)
No. of all‐cause deaths	1,918 (33.8%)
No. of CRC‐related deaths	1,358 (23.9%)

1 Median and quartiles in parenthesis.

Abbreviations: AJCC, American Joint Committee on Cancer; CRC, colorectal cancer; SOCCS, Study of Colorectal Cancer in Scotland.

**Table 2:** Summarized results of association between variants at nominal significance (*p* < 0.05) and PRS with CRC survival

Genetic marker				Overall survival			CRC‐specific survival		
Variant	Gene	EA	MAF^2^	HR^1^ (95% CI)	*p* value	Pfdr	HR^1^ (95% CI)	*p* value	Pfdr
rs10161980	AL139383.1	G	0.40	1.08 (1.01–1.15)	0.019	0.251	1.06 (0.98–1.14)	0.160	0.832
rs10994860	A1CF	T	0.17	1.11 (1.02–1.20)	0.016	0.251	1.13 (1.02–1.24)	0.018	0.603
rs11196171	TCF7L2	G	0.22	0.91 (0.83–0.98)	0.017	0.251	0.93 (0.85–1.03)	0.161	0.832
rs12143541	TTC22	G	0.15	1.13 (1.04–1.24)	0.006	0.251	1.13 (1.02–1.25)	0.023	0.603
rs16959063	FMN1	A	0.01	0.72 (0.53–0.97)	0.034	0.380	0.83 (0.59–1.16)	0.273	0.832
rs174537	MYRF	T	0.34	1.08 (1.01–1.16)	0.019	0.251	1.05 (0.97–1.14)	0.218	0.832
rs2696839	Intergenic	C	0.48	0.95 (0.90–1.02)	0.143	0.642	0.93 (0.86–1.00)	0.048	0.603
rs3087967	C11orf53	C	0.33	0.92 (0.86–0.99)	0.017	0.251	0.93 (0.86–1.00)	0.062	0.616
rs3217810	CCND2	T	0.14	1.13 (1.02–1.25)	0.016	0.251	1.13 (1.00–1.27)	0.044	0.603
rs3217874	CCND2	T	0.42	1.07 (1.00–1.14)	0.050	0.438	1.06 (0.98–1.14)	0.139	0.823
rs34405347	Intergenic	G	0.08	0.84 (0.74–0.95)	0.007	0.251	0.85 (0.73–0.99)	0.042	0.603
rs35509282	Intergenic	A	0.12	0.94 (0.86–1.04)	0.256	0.772	0.88 (0.78–0.99)	0.033	0.603
rs4759277	LRP1	A	0.38	1.07 (1.00–1.14)	0.051	0.438	1.09 (1.01–1.18)	0.028	0.603
rs6065668	Intergenic	T	0.28	0.90 (0.83–0.96)	0.003	0.251	0.89 (0.82–0.97)	0.011	0.603
rs7495132	CRTC3	T	0.12	1.03 (0.93–1.13)	0.602	0.888	1.13 (1.01–1.26)	0.032	0.603
rs847208	LINC01081	A	0.37	0.93 (0.88–1.00)	0.042	0.416	0.92 (0.85–0.99)	0.027	0.603
rs9537521	AL139383.1	A	0.38	1.10 (1.02–1.17)	0.008	0.251	1.06 (0.98–1.15)	0.153	0.832
rs9929218	CDH1	A	0.28	0.93 (0.86–0.99)	0.035	0.380	0.96 (0.88–1.04)	0.294	0.836
PRS		NA		1.00 (0.95–1.04)	0.864	NA	1.03 (0.97–1.08)	0.340	NA

1 Hazard ratios are derived from Cox regression models adjusted for age at diagnosis, sex and AJCC stage.

2 Minor allele frequency is the minor allele prevalence in SOCCS.

Abbreviations: CI, confidence interval; CRC, colorectal cancer; EA, effect alleles; HR, hazard ratio; MAF, minor allele frequency; NA, not available; Pfdr, *p* values after correction for false positive rates; PRS, polygenic risk score.

We used a concept of statistical significance as decision criteria to define if the risk variants have an effect on survival. This concept has been criticized in the literature as subjective and commonly misused.^24^, ^25^ We additionally looked into direction of effects to overcome limitations of statistical significance. We hypothesized that in case of no effects, the CRC risk variants will cause improvement or impairment of OS and CSS in equal proportion. Ambiguous AT and CG genetic variants were excluded and out of 124 tested variants only 52 risk variants were also associated with worse OS (proportion of risk variants associated with worse OS 41%, 95% CI = 33–51%) and 58 were associated with decrease in CSS (proportion of risk variants associated with worse CSS 47%, 95% CI = 38–56%). Though not reaching suggested significance level (*p* ≤ 0.05), these results are consistent with directions of effects observed in previous studies.^3^, ^4^

None of the nominally significant genetic variants have known detrimental clinically relevant effects on gene function (Supporting Information Table S6). Rs3087967, which is located 3'UTP of C11orf53, is known to be associated with higher *COLCA2* and *C11orf53* expression in colon transverse tissue for C allele.^9^, ^20^ However, little is known about *COLCA2* and C11orf53 functions. rs4759277, which is associated with worse OS and located within intron region of *LRP1*, is likely to affect binding of transcription factors and associated with LRP1 gene expression in tibial artery and sun‐exposed skin.^20^ Another variant with nominally significant effects on both OS and CSS is rs10994860 variant. It is located 5'UTP of APOBEC1 complementation factor (*A1CF*) and likely to affect binding (regulomeDB score 2b). The same variant has been previously associated with estimated glomerular filtration rate (eGFR), a measure of the kidney's filtration ability in serum.^26^

This is the first study capturing all currently identified CRC risk genetic loci (*n* = 92) and investigating their associations with survival outcomes in a population‐based study. Our results indicate that overall genetic CRC susceptibility measured by GWAS‐identified variants is not statistically significantly associated with survival after CRC diagnosis. With regard to each variant, our study, which had acceptable power for HR > 1.2, found multiple variants (14 for OS and 10 for CSS) associated with survival outcomes at *p* < 0.05, although the significance fails to survive correction for multiple testing.

Similarly, previous studies identified some CRC‐risk variants^27^, ^28^ that might be associated with survival after CRC diagnosis, these findings were not immune to false‐positive results from multiple testing. A widely studied variant, rs9929218 lies in the intron of *CDH1* gene encoding E‐cadherin, the loss of function of which can cause tumor progression and metastasis.^29^ Previous studies reported that the CRC‐risk decreasing allele (A) is statistically significantly associated with poor survival outcomes.^27^, ^28^ However, in contrast, we observed a potentially favorable effect (though not statistically significant after multiple‐testing correction) of the A allele on OS in our study (i.e., the direction of CRC risk and prognosis are consistent in our study). Smith *et al*. reported that the A allele is significantly associated with poor response to chemotherapy, implying a possible gene × therapy interaction for this variant.^28^ However, our study is limited by data on response to chemotherapy being unavailable so that this could not be explored further. Notably, there has been other evidence showing that rs9929218 may modify CRC susceptibility by interacting with other factors such as height and alcohol consumption.^30^ Investigation of possible gene–environment interactions should be considered in future efforts with large sample sizes to further dissect the prognostic effect of this variant in CRC patients. Consistently, when looking for overall direction of effects among 124 tested variants we noted little evidence of potentially detrimental effects of CRC risk on survival with only 41 and 47% of risk variants showing association with poor (HR > 1) OS and CSS in our study.

Previously we have shown that SOCCS study is representative of British and Scottish populations and cases from SOCCS cluster tightly with population‐based controls from SOCCS and Generation Scotland.^31^, ^32^ The allele frequencies of studied genetic variants are in range expected for European populations (Supporting Information Table S1). However, the results may be not generalizable to populations of cancer patients where substantial differences in allele frequencies and/or treatment and disease management are anticipated.

In conclusion, our study finds that overall genetic susceptibility to CRC captured by known CRC risk variants is not statistically significantly associated with survival outcomes of CRC. However, possible roles of each variant in CRC progression remain to be explored. Our study indicated that the heritable variation of patient survival may have distinct genetic determinants from CRC susceptibility. The previous GWAS on CRC survival with 3,494 cases identified no variants at genome‐wide significance (*p* < 5E−8),^33^ meriting future collaborative efforts of aggregating larger CRC cohorts to illuminate genetic structure of survival outcomes for CRC patients.

## Supporting information


**Figure S1** Diagram of patient selection
**Table S1** Summary of 130 GWAS‐identified variants associated with CRC susceptibility.
**Table S2** Summarized results of association between variants at nominal significance (p < 0.05) and CRC survival stratified by stage
**Table S3** summarized results of association between variants at nominal significance (p < 0.05) and CRC survival stratified by sex
**Table S4** summarized results of association between variants at nominal significance (p < 0.05) and CRC survival stratified by tumor site
**Table S5** summarized results of model performance using LASSO regression to predict survival outcomes

**Table S6** Functional annotation of analyzed genetic variants

## Data Availability

The data that support the findings of our study are available upon reasonable request from the corresponding authors. The data are not publicly available due to privacy or ethical restrictions.
