# Supplementary material for: Effects of common genetic variants associated with colorectal cancer risk on survival outcomes after diagnosis: A large population‐based cohort study
Source: Int J Cancer. 2019 Jul 27;145(9):2427–32. doi: 10.1002/ijc.32550 (PMC6771941; doi:10.1002/ijc.32550)
Supplement: Supplementary file 1 — Figure S1 Diagram of patient selection Table S1 Summary of 130 GWAS‐identified variants associated with CRC susceptibility. Table S2 Summarized results of association between variants at nominal significance (p < 0.05) and CRC survival stratified by stage Table S3 summarized results of association between variants at nominal significance (p < 0.05) and CRC survival stratified by sex Table S4 summarized results of association between variants at nominal significance (p < 0.05) and CRC survival stratified by tumor site Table S5 summarized results of model performance using LASSO regression to predict survival outcomes [file IJC-145-2427-s001.docx]

**Supplementary materials**

Effects of common genetic variants associated with colorectal cancer risk on survival outcomes after diagnosis: a large population-based cohort study

Yazhou He, Evropi Theodoratou, Xue Li, Farhat VN. Din, Peter Vaughan-Shaw, Victoria Svinti, Susan M. Farrington, Harry Campbell, Malcolm G. Dunlop^*^, Maria Timofeeva^*^

Figure S1 diagram of patient selection

SOCCS CRC cases with GWAS data

(n=6,366)

CRC cases excluded:

Failed to pass quality control (n=154)

Missing data regarding age at diagnosis, date of definitive treatment, or AJCC stage (n=537)

CRC cases included (n=5,675)*

*for rs75954926, the number of eventually included cases is 4,817 due to missing genotype data.

CRC, colorectal cancer; SOCCS, Study of Colorectal Cancer in Scotland.

Table S1 Summary of 130 GWAS-identified variants associated with CRC susceptibility.

|  |  |  |  |  |  | **Minor Allele Frequency** | | | | |  |  |
| --- | --- | --- | --- | --- | --- | --- | --- | --- | --- | --- | --- | --- |
| **Variant** | **Locus** | **Position*** | **MAF**** | **CRC-risk allele** | **Minor allele** | **CEU** | **TSI** | **FIN** | **GRB** | **IBS** | **Gene** | **Reference** |
| rs10049390 | 3q22.2 | 133701119 | 0.24 | A | G | 0.24 | 0.28 | 0.29 | 0.26 | 0.36 | SLCO2A1 | Huyghe,Nat Genet,2019^1^ |
| rs10152518 | 15q23 | 68177162 | 0.19 | G | G | 0.14 | 0.18 | 0.23 | 0.18 | 0.39 | Intergenic | Law P, Nat Com, 2018^2^ |
| rs10161980 | 13q13.2 | 34093518 | 0.40 | C | G | 0.37 | 0.39 | 0.33 | 0.34 | 0.29 | AL139383.1 | Schmit SL, JNCI, 2018^3^ |
| rs1035209 | 10q24.2 | 101345366 | 0.21 | T | T | 0.22 | 0.20 | 0.17 | 0.19 | 0.29 | Intergenic | Whiffin N, Hum Mol Genet, 2014^4^ |
| rs10411210 | 19q13.11 | 33532300 | 0.08 | C | T | 0.07 | 0.10 | 0.09 | 0.12 | 0.14 | RHPN2 | COGENT, Nat Genet, 2008^5^ |
| rs10774214 | 12p13.32 | 4368352 | 0.35 | T | T | 0.39 | 0.35 | 0.41 | 0.35 | 0.43 | CCND2-AS1 | Jia WH, Nat Genet, 2013^6^ |
| rs1078643 | 17p12 | 10707241 | 0.24 | A | G | 0.17 | 0.28 | 0.26 | 0.25 | 0.32 | TMEM238L | Law P, Nat Com, 2018^2^ |
| rs10795668 | 10p14 | 8701219 | 0.32 | G | A | 0.32 | 0.31 | 0.30 | 0.37 | 0.39 | RNA5SP299 | Tomlinson IP, Nat Genet, 2008^7^ |
| rs10849432 | 12p13.31 | 6385727 | 0.11 | T | C | 0.07 | 0.09 | 0.09 | 0.10 | 0.14 | Intergenic | Zhang B, Nat Genet, 2014^8^ |
| rs10849438 | 12p13.31 | 6412036 | 0.13 | G | G | 0.09 | 0.17 | 0.18 | 0.13 | 0.11 | Intergenic | Law P, Nat Com, 2018^2^ |
| rs10911251 | 1q25.3 | 183081194 | 0.43 | A | C | 0.47 | 0.41 | 0.52 | 0.46 | 0.32 | LAMC1 | Whiffin N, Hum Mol Genet, 2014^4^ |
| rs10936599 | 3q26.2 | 169492101 | 0.24 | C | T | 0.26 | 0.20 | 0.28 | 0.29 | 0.14 | MYNN | Houlston RS, Nat Genet, 2010^9^ |
| rs10951878 | 7p12.3 | 46926695 | 0.50 | C | T | 0.46 | 0.55 | 0.58 | 0.46 | 0.61 | AC004870.4 | Law P, Nat Com, 2018^2^ |
| rs10980628 | 9q31.3 | 113671403 | 0.22 | C | C | 0.23 | 0.20 | 0.12 | 0.22 | 0.18 | LPAR1 | Huyghe,Nat Genet,2019^1^ |
| rs10994860 | 10q11.23 | 52645424 | 0.17 | C | T | 0.19 | 0.18 | 0.33 | 0.14 | 0.07 | A1CF | Schmit SL, JNCI, 2018^3^ |
| rs11064437 | 12p13.31 | 6982162 | 0.00 | C | T | NA | 0.01 | NA | 0.01 | NA | TPI1/RPL13P5 | Zeng C, Gastroenterology, 2016^10^ |
| rs11169552 | 12q13.13 | 51155663 | 0.26 | C | T | 0.19 | 0.18 | 0.37 | 0.26 | 0.14 | ATF1 | Houlston RS, Nat Genet, 2010^9^ |
| rs11196171 | 10q25.2 | 114724473 | 0.22 | G | G | 0.16 | 0.22 | 0.23 | 0.24 | 0.21 | TCF7L2 | Law P, Nat Com, 2018^2^ |
| rs11610543 | 12q12 | 43134191 | 0.48 | G | A | 0.50 | 0.36 | 0.60 | 0.53 | 0.39 | Intergenic | Huyghe,Nat Genet,2019^1^ |
| rs11632715 | 15q13.3 | 33004247 | 0.48 | A | A | 0.48 | 0.43 | 0.52 | 0.45 | 0.50 | Intergenic | Tomlinson IP, PLoS Genet, 2011^11^ |
| rs11692435 | 2q11.2 | 98275354 | 0.07 | G | A | 0.08 | 0.04 | 0.22 | 0.03 | 0.07 | ACTR1B | Law P, Nat Com, 2018^2^ |
| rs11893063 | 2q33.1 | 199601925 | 0.46 | A | A | 0.36 | 0.49 | 0.54 | 0.42 | 0.61 | AC019330.1 | Law P, Nat Com, 2018^2^ |
| rs11903757 | 2q32.3 | 192587204 | 0.16 | C | C | 0.17 | 0.13 | 0.26 | 0.11 | 0.11 | Intergenic | Peters U, Gastroenterology, 2012^12^ |
| rs12143541 | 1p32.3 | 55247852 | 0.15 | G | G | 0.22 | 0.13 | 0.11 | 0.15 | 0.18 | TTC22 | Law P, Nat Com, 2018^2^ |
| rs12241008 | 10q25.2 | 114280702 | 0.11 | C | C | 0.07 | 0.09 | 0.12 | 0.09 | 0.11 | VTI1A | Wang H, Nat Comm, 2014^13^ |
| rs12427600 | 13q13.3 | 37460648 | 0.25 | C | C | 0.21 | 0.15 | 0.33 | 0.25 | 0.18 | SMAD9 | Law P, Nat Com, 2018^2^ |
| rs12522693 | 5q23.3 | 130195731 | 0.13 | G | A | 0.19 | 0.14 | 0.17 | 0.14 | 0.04 | Intergenic | Jiang K, Oncotarget, 2015^14^ |
| rs12603526 | 17p13.3 | 800593 | 0.02 | C | C | 0.02 | 0.01 | 0.03 | 0.01 | NA | NXN | Zhang B, Nat Genet, 2014^8^ |
| rs12635946 | 3q13.2 | 112916918 | 0.36 | C | T | 0.42 | 0.45 | 0.29 | 0.40 | 0.46 | Intergenic | Law P, Nat Com, 2018^2^ |
| rs12672022 | 7p13 | 45136423 | 0.17 | T | C | 0.16 | 0.14 | 0.18 | 0.19 | 0.29 | TBRG4 | Huyghe,Nat Genet,2019^1^ |
| rs12979278 | 19q13.33 | 49218602 | 0.40 | T | C | 0.46 | 0.49 | 0.68 | 0.45 | 0.46 | MAMSTR | Law P, Nat Com, 2018^2^ |
| rs13020391 | 2q35 | 219184436 | 0.39 | C | T | 0.36 | 0.33 | 0.31 | 0.37 | 0.46 | PNKD | Law P, Nat Com, 2018^2^ |
| rs1321310 | 6p21.2 | 36623124 | 0.25 | C | C | 0.31 | 0.18 | 0.14 | 0.22 | 0.18 | Intergenic | Law P, Nat Com, 2018^2^ |
| rs1330889 | 13q22.3 | 78609615 | 0.12 | C | T | 0.12 | 0.13 | 0.12 | 0.24 | 0.54 | LINC00446 | Law P, Nat Com, 2018^2^ |
| rs1370821 | 4q22.2 | 94943383 | 0.45 | T | T | 0.45 | 0.40 | 0.31 | 0.46 | 0.39 | Intergenic | Schmit SL, JNCI, 2018^3^ |
| rs1391441 | 4q24 | 106128760 | 0.29 | A | G | 0.32 | 0.35 | 0.30 | 0.31 | 0.25 | TET2 | Huyghe,Nat Genet,2019^1^ |
| rs1412834 | 9p21.3 | 22110131 | 0.50 | T | C | 0.48 | 0.58 | 0.42 | 0.52 | 0.54 | CDKN2B-AS1 | Law P, Nat Com, 2018^2^ |
| rs1445011 | 5p13.1 | 40280202 | 0.31 | C | C | 0.32 | 0.24 | 0.20 | 0.29 | 0.25 | Intergenic | Law P, Nat Com, 2018^2^ |
| rs1570405 | 14q22.2 | 54554234 | 0.33 | G | G | 0.34 | 0.30 | 0.24 | 0.35 | 0.32 | Intergenic | Tomlinson IP, Nat Genet, 2008^7^ |
| rs16878812 | 6p21.31 | 35569562 | 0.12 | A | G | 0.09 | 0.07 | 0.06 | 0.14 | 0.14 | FKBP5 | Law P, Nat Com, 2018^2^ |
| rs16892766 | 8q23.3 | 117630683 | 0.10 | C | C | 0.09 | 0.08 | 0.12 | 0.08 | 0.07 | Intergenic | Tomlinson IP, Nat Genet, 2008^7^ |
| rs16959063 | 15q13.3 | 33105730 | 0.01 | A | A | NA | 0.02 | 0.01 | NA | NA | FMN1 | Law P, Nat Com, 2018^2^ |
| rs16969681 | 15q13.3 | 32993111 | 0.10 | T | T | 0.06 | 0.10 | 0.06 | 0.05 | 0.11 | SCG5 | Tomlinson IP, Nat Genet, 2008^7^ |
| rs17035289 | 4q24 | 106048291 | 0.15 | T | C | 0.14 | 0.19 | 0.27 | 0.16 | 0.14 | Intergenic | Law P, Nat Com, 2018^2^ |
| rs17094983 | 14q23.1 | 59189361 | 0.11 | G | A | 0.07 | 0.14 | 0.14 | 0.13 | 0.18 | LINC01500 | Huyghe,Nat Genet,2019^1^ |
| rs1741640 | 20q13.33 | 60932414 | 0.22 | C | T | 0.28 | 0.23 | 0.15 | 0.25 | 0.36 | LAMA5 | Law P, Nat Com, 2018^2^ |
| rs174537 | 11q12.2 | 61552680 | 0.34 | G | T | 0.35 | 0.25 | 0.47 | 0.36 | 0.46 | MYRF | Zhang B, Nat Genet, 2014^8^ |
| rs17816465 | 15q13.3 | 33156386 | 0.22 | A | A | 0.14 | 0.15 | 0.17 | 0.17 | 0.25 | FMN1 | Law P, Nat Com, 2018^2^ |
| rs17836917 | 17q12 | 32047282 | 0.02 | G | A | 0.04 | 0.02 | 0.01 | 0.04 | 0.04 | ASIC2 | Jiang K, Oncotarget, 2015^14^ |
| rs1800469 | 19q13.2 | 41860296 | 0.28 | G | A | 0.30 | 0.43 | 0.21 | 0.27 | 0.36 | TMEM91 | Zhang B, Nat Genet, 2014^8^ |
| rs1810502 | 20q13.13 | 49057488 | 0.42 | C | T | 0.39 | 0.43 | 0.44 | 0.44 | 0.54 | Intergenic | Schmit SL, JNCI, 2018^3^ |
| rs186722897 | 4q32.2 | 163374639 | 0.06 | T | T | 0.02 | 0.02 | 0.03 | 0.04 | 0.07 | Intergenic | Law P, Nat Com, 2018^2^ |
| rs2070699 | 6p24.1 | 12292772 | 0.48 | T | T | 0.47 | 0.41 | 0.47 | 0.53 | 0.61 | EDN1 | Law P, Nat Com, 2018^2^ |
| rs2179593 | 20q13.12 | 42660286 | 0.27 | A | C | 0.26 | 0.27 | 0.33 | 0.22 | 0.25 | TOX2 | Law P, Nat Com, 2018^2^ |
| rs2186607 | 11q22.1 | 101656397 | 0.50 | T | A | 0.46 | 0.48 | 0.50 | 0.54 | 0.46 | TRPC6 | Huyghe,Nat Genet,2019^1^ |
| rs2238126 | 12p13.2 | 12009741 | 0.16 | G | G | 0.21 | 0.13 | 0.16 | 0.16 | 0.11 | ETV6 | Wang M, Nat Comm, 2016^15^ |
| rs2279290 | 3p14.1 | 66430811 | 0.20 | G | G | 0.23 | 0.18 | 0.19 | 0.20 | 0.29 | LRIG1 | Law P, Nat Com, 2018^2^ |
| rs2295444 | 20q11.22 | 33173883 | 0.48 | C | T | 0.46 | 0.53 | 0.59 | 0.48 | 0.46 | PIGU | Schmit SL, JNCI, 2018^3^ |
| rs2423279 | 20p12.3 | 7812350 | 0.24 | C | C | 0.22 | 0.35 | 0.25 | 0.21 | 0.25 | AL031679.1 | Zhang B, Nat Genet, 2014^8^ |
| rs2516420 | 6p21.32 | 31449620 | 0.07 | C | T | 0.10 | 0.08 | 0.04 | 0.10 | 0.14 | HCP5 | Huyghe,Nat Genet,2019^1^ |
| rs2696839 | 16q24.1 | 86340448 | 0.48 | G | G | 0.48 | 0.45 | 0.63 | 0.47 | 0.68 | Intergenic | Schmit SL, JNCI, 2018^3^ |
| rs2732875 | Xp22.2 | 9763898 | 0.23 | C | C | 0.19 | 0.25 | 0.20 | 0.21 | 0.21 | SHROOM2 | Law P, Nat Com, 2018^2^ |
| rs2735940 | 5p15.33 | 1296486 | 0.50 | A | G | 0.49 | 0.51 | 0.50 | 0.53 | 0.39 | TERT | Schmit SL, JNCI, 2018^3^ |
| rs28488 | 20p12.3 | 6762221 | 0.37 | T | C | 0.34 | 0.30 | 0.42 | 0.37 | 0.18 | BMP2 | Huyghe,Nat Genet,2019^1^ |
| rs285245 | 19p13.11 | 16420817 | 0.12 | T | T | 0.11 | 0.14 | 0.10 | 0.17 | 0.21 | AC020911.2 | Law P, Nat Com, 2018^2^ |
| rs3087967 | 11q23.1 | 111156836 | 0.33 | T | T | 0.22 | 0.29 | 0.24 | 0.31 | 0.25 | C11orf53 | Law P, Nat Com, 2018^2^ |
| rs3131043 | 6p21.33 | 30758466 | 0.45 | G | G | 0.379 | 0.551 | 0.359 | 0.434 | 0.425 | HCG20 | Law P, Nat Com, 2018^2^ |
| rs3184504 | 12q24.12 | 111884608 | 0.48 | C | T | 0.45 | 0.53 | 0.39 | 0.47 | 0.61 | SH2B3 | Schumacher FR, Nat Commun, 2015^16^ |
| rs3217810 | 12p13.32 | 4388271 | 0.14 | T | T | 0.16 | 0.10 | 0.12 | 0.12 | 0.07 | CCND2 | Whiffin N, Hum Mol Genet, 2014^4^ |
| rs3217874 | 12p13.32 | 4400808 | 0.47 | T | T | 0.41 | 0.35 | 0.45 | 0.50 | 0.36 | CCND2 | Huyghe,Nat Genet,2019^1^ |
| rs34405347 | 9q22.33 | 101679752 | 0.08 | T | G | 0.08 | 0.03 | 0.10 | 0.08 | 0.04 | Intergenic | Huyghe,Nat Genet,2019^1^ |
| rs35107139 | 14q22.2 | 54419106 | 0.40 | C | C | 0.38 | 0.45 | 0.37 | 0.37 | 0.57 | BMP4 | Law P, Nat Com, 2018^2^ |
| rs35360328 | 3p22.1 | 40924962 | 0.16 | A | A | 0.18 | 0.15 | 0.21 | 0.12 | 0.07 | Intergenic | Schumacher FR, Nat Commun, 2015^16^ |
| rs35509282 | 4q32.2 | 163333405 | 0.12 | A | A | 0.11 | 0.08 | 0.18 | 0.11 | 0.11 | Intergenic | Schmit SL, Carcinogenesis, 2014^17^ |
| rs3787089 | 20q13.33 | 62316630 | 0.35 | C | C | 0.38 | 0.27 | 0.28 | 0.34 | 0.29 | RTEL1 | Law P, Nat Com, 2018^2^ |
| rs3801081 | 7p12.3 | 47511161 | 0.33 | G | A | 0.35 | 0.27 | 0.31 | 0.36 | 0.39 | TNS3 | Law P, Nat Com, 2018^2^ |
| rs3824999 | 11q13.4 | 74345550 | 0.49 | G | G | 0.48 | 0.55 | 0.57 | 0.51 | 0.54 | POLD3 | Dunlop MG, Nat Genet, 2012^18^ |
| rs3987 | 4q26 | 118759055 | 0.36 | G | G | 0.38 | 0.47 | 0.41 | 0.36 | 0.61 | LINC02264 | Real LM, PLoS One, 2014^19^ |
| rs4313119 | 8q24.21 | 128571855 | 0.22 | G | T | 0.19 | 0.25 | 0.32 | 0.25 | 0.18 | Intergenic | Huyghe,Nat Genet,2019^1^ |
| rs4450168 | 11p15.4 | 10286755 | 0.18 | C | C | 0.21 | 0.16 | 0.15 | 0.15 | 0.29 | SBF2 | Law P, Nat Com, 2018^2^ |
| rs448513 | 2q24.2 | 159964552 | 0.32 | C | C | 0.31 | 0.33 | 0.49 | 0.33 | 0.43 | TANC1 | Huyghe,Nat Genet,2019^1^ |
| rs45597035 | 13q22.1 | 73649152 | 0.33 | A | G | 0.36 | 0.34 | 0.54 | 0.39 | 0.32 | KLF5 | Law P, Nat Com, 2018^2^ |
| rs4711689 | 6p21.1 | 41692812 | 0.41 | A | G | 0.48 | 0.48 | 0.46 | 0.43 | 0.39 | TFEB | Zeng C, Gastroenterology, 2016^10^ |
| rs4759277 | 12q13.3 | 57533690 | 0.38 | A | A | 0.30 | 0.31 | 0.34 | 0.35 | 0.46 | LRP1 | Huyghe,Nat Genet,2019^1^ |
| rs4776316 | 15q22.31 | 67007813 | 0.25 | A | G | 0.27 | 0.28 | 0.25 | 0.31 | 0.29 | SMAD6 | Law P, Nat Com, 2018^2^ |
| rs4811050 | 20q13.13 | 48980670 | 0.19 | A | A | 0.23 | 0.14 | 0.28 | 0.22 | 0.18 | Intergenic | Law P, Nat Com, 2018^2^ |
| rs4919687 | 10q24.32 | 104595248 | 0.28 | G | A | 0.32 | 0.31 | 0.31 | 0.25 | 0.39 | CYP17A1 | Zeng C, Gastroenterology, 2016^10^ |
| rs4939827 | 18q21.1 | 46453463 | 0.44 | T | T | 0.45 | 0.58 | 0.54 | 0.52 | 0.64 | SMAD7 | Tenesa A, Nat Genet, 2008^20^ |
| rs4944940 | 11q13.4 | 74415252 | 0.04 | G | A | 0.03 | 0.03 | 0.05 | 0.07 | NA | CHRDL2 | Law P, Nat Com, 2018^2^ |
| rs56324967 | 15q22.33 | 67402824 | 0.32 | C | T | 0.26 | 0.34 | 0.37 | 0.26 | 0.39 | SMAD3 | Huyghe,Nat Genet,2019^1^ |
| rs5934683 | Xp22.2 | 9751474 | 0.37 | T | T | 0.36 | 0.44 | 0.35 | 0.31 | 0.49 | GPR143 | Dunlop MG, Nat Genet, 2012^18^ |
| rs6065668 | 20q13.12 | 42532821 | 0.28 | C | T | 0.26 | 0.24 | 0.23 | 0.30 | 0.11 | Intergenic | Tanikawa C, Carcinogenesis, 2018^21^ |
| rs6066825 | 20q13.13 | 47340117 | 0.34 | A | G | 0.36 | 0.41 | 0.28 | 0.39 | 0.32 | PREX1 | Law P, Nat Com, 2018^2^ |
| rs6085661 | 20p12.3 | 6693128 | 0.42 | T | T | 0.38 | 0.35 | 0.38 | 0.40 | 0.36 | Intergenic | Tomlinson IP, Nat Genet, 2008^7^ |
| rs6091213 | 20q13.13 | 49384745 | 0.27 | C | C | 0.26 | 0.21 | 0.22 | 0.28 | 0.14 | Intergenic | Law P, Nat Com, 2018^2^ |
| rs61336918 | 16q23.2 | 80007266 | 0.33 | A | A | 0.28 | 0.36 | 0.22 | 0.29 | 0.43 | Intergenic | Law P, Nat Com, 2018^2^ |
| rs61776719 | 1p34.3 | 38461319 | 0.46 | C | C | 0.38 | 0.47 | 0.43 | 0.47 | 0.57 | FHL3 | Law P, Nat Com, 2018^2^ |
| rs62404966 | 6p12.1 | 55712124 | 0.24 | C | T | 0.22 | 0.24 | 0.23 | 0.28 | 0.36 | BMP5 | Law P, Nat Com, 2018^2^ |
| rs639933 | 5q31.1 | 134467751 | 0.39 | C | C | 0.37 | 0.29 | 0.40 | 0.39 | 0.50 | C5orf66 | Law P, Nat Com, 2018^2^ |
| rs647161 | 5q31.1 | 134499092 | 0.34 | A | C | 0.32 | 0.30 | 0.37 | 0.37 | 0.36 | C5orf66 | Jia WH, Nat Genet, 2013^6^ |
| rs6658977 | 1q41 | 222049820 | 0.36 | T | T | 0.34 | 0.44 | 0.46 | 0.36 | 0.36 | LINC02257 | Law P, Nat Com, 2018^2^ |
| rs6928864 | 6q21 | 105966894 | 0.08 | C | A | 0.09 | 0.05 | 0.10 | 0.12 | 0.07 | Intergenic | Law P, Nat Com, 2018^2^ |
| rs6933790 | 6p21.1 | 41672769 | 0.16 | T | C | 0.22 | 0.20 | 0.13 | 0.25 | 0.14 | TFEB | Law P, Nat Com, 2018^2^ |
| rs6983267 | 8q24.21 | 128413305 | 0.45 | G | G | 0.48 | 0.38 | 0.52 | 0.54 | 0.54 | CASC8 | Tomlinson I, Nat Genet, 2007^22^ |
| rs704017 | 10q22.3 | 80819132 | 0.40 | G | A | 0.46 | 0.47 | 0.35 | 0.39 | 0.57 | ZMIZ1-AS1 | Zhang B, Nat Genet, 2014^8^ |
| rs72013726 | 12q24.21 | 115890835 | 0.46 | C | CACAA | 0.47 | 0.62 | 0.37 | 0.51 | 0.71 | Intergenic | Schmit SL, JNCI, 2018^3^ |
| rs72647484 | 1p36.12 | 22587728 | 0.09 | T | C | 0.11 | 0.08 | 0.09 | 0.08 | 0.07 | Intergenic | Al-Tassan NA, Sci Rep, 2015^23^ |
| rs72942485 | 3q13.2 | 112999560 | 0.01 | G | A | 0.02 | 0.03 | 0.01 | 0.02 | NA | BOC | Huyghe,Nat Genet,2019^1^ |
| rs73068325 | 19q13.43 | 59079096 | 0.19 | T | T | 0.21 | 0.24 | 0.17 | 0.19 | 0.25 | MZF1-AS1 | Huyghe,Nat Genet,2019^1^ |
| rs73208120 | 12q24.22 | 117747590 | 0.11 | G | G | 0.12 | 0.08 | 0.11 | 0.11 | 0.14 | NOS1 | Schumacher FR, Nat Commun, 2015^16^ |
| rs73376930 | 15q13.3 | 33012502 | 0.22 | G | G | 0.14 | 0.28 | 0.24 | 0.17 | 0.14 | GREM1 | Law P, Nat Com, 2018^2^ |
| rs73975588 | 17p13.3 | 816741 | 0.12 | A | C | 0.11 | 0.14 | 0.11 | 0.13 | 0.32 | NXN | Law P, Nat Com, 2018^2^ |
| rs7398375 | 12q13.3 | 57540848 | 0.25 | C | G | 0.30 | 0.24 | 0.28 | 0.26 | 0.14 | LRP1 | Law P, Nat Com, 2018^2^ |
| rs7495132 | 15q26.1 | 91172901 | 0.12 | T | T | 0.11 | 0.07 | 0.12 | 0.06 | 0.11 | CRTC3 | Law P, Nat Com, 2018^2^ |
| rs75686861 | 4q31.21 | 145621328 | 0.10 | A | A | 0.06 | 0.10 | 0.09 | 0.09 | 0.04 | HHIP | Law P, Nat Com, 2018^2^ |
| rs7593422 | 2q33.1 | 200131695 | 0.45 | T | A | 0.42 | 0.54 | 0.45 | 0.48 | 0.46 | SATB2 | Law P, Nat Com, 2018^2^ |
| rs75954926 | 17q25.3 | 81061048 | 0.33 | G | A | 0.30 | 0.32 | 0.35 | 0.39 | 0.43 | AC144831.1 | Huyghe,Nat Genet,2019^1^ |
| rs7708610 | 5p13.1 | 40102443 | 0.36 | A | A | 0.35 | 0.36 | 0.35 | 0.39 | 0.39 | Intergenic | Huyghe,Nat Genet,2019^1^ |
| rs77776598 | 5p15.33 | 1240998 | 0.06 | C | C | 0.06 | 0.06 | 0.07 | 0.03 | NA | SLC6A18 | Law P, Nat Com, 2018^2^ |
| rs78341008 | 13q22.1 | 73791554 | 0.08 | C | C | 0.06 | 0.09 | 0.04 | 0.06 | 0.04 | Intergenic | Huyghe,Nat Genet,2019^1^ |
| rs7993934 | 13q34 | 111074915 | 0.35 | T | C | 0.35 | 0.43 | 0.34 | 0.31 | 0.36 | COL4A2 | Law P, Nat Com, 2018^2^ |
| rs847208 | 16q24.1 | 86254051 | 0.37 | A | C | 0.32 | 0.45 | 0.38 | 0.30 | 0.50 | LINC01081 | Tanikawa C, Carcinogenesis, 2018^21^ |
| rs899244 | 16q24.1 | 86700030 | 0.22 | T | T | 0.22 | 0.18 | 0.26 | 0.28 | 0.07 | AC009154.1 | Law P, Nat Com, 2018^2^ |
| rs9271770 | 6p21.32 | 32594248 | 0.19 | A | G | 0.17 | 0.18 | 0.31 | 0.20 | 0.25 | HLA-DQA1 | Law P, Nat Com, 2018^2^ |
| rs9537521 | 13q13.2 | 34038180 | 0.38 | G | A | 0.21 | 0.24 | 0.24 | 0.30 | 0.18 | AL139383.1 | Law P, Nat Com, 2018^2^ |
| rs961253 | 20p12.3 | 6404281 | 0.38 | A | A | 0.44 | 0.37 | 0.32 | 0.35 | 0.32 | Intergenic | COGENT, Nat Genet, 2008^5^ |
| rs9831861 | 3p21.1 | 53088285 | 0.41 | G | T | 0.48 | 0.38 | 0.34 | 0.39 | 0.43 | AC096887.1 | Law P, Nat Com, 2018^2^ |
| rs983318 | 17q24.3 | 70413253 | 0.24 | A | A | 0.19 | 0.20 | 0.23 | 0.24 | 0.25 | LINC00511 | Huyghe,Nat Genet,2019^1^ |
| rs9929218 | 16q22.1 | 68820946 | 0.28 | G | A | 0.29 | 0.31 | 0.20 | 0.34 | 0.36 | CDH1 | COGENT, Nat Genet, 2008^5^ |
| rs994308 | 20p12.3 | 6603622 | 0.39 | C | T | 0.35 | 0.35 | 0.48 | 0.41 | 0.61 | Intergenic | Huyghe,Nat Genet,2019^1^ |

*Position for each variant is based on Genome Reference Consortium Human Build 37 (GRCh37);
**MAF is the minor allele frequency in the SOCCS
CRC, colorectal cancer; MAF, minor allele frequency; SOCCS, Study Of Colorectal Cancer in Scotland; Utah Residents (CEPH) with Northern and Western European Ancestry (CEU); Toscani in Italia (TSI); Finnish in Finland (GBR); Iberian Population in Spain (YRI).

Table S2 Summarized results of association between variants at nominal significance (p<0.05) and CRC survival stratified by stage

|  |  | Overall survival |  |  |  | CRC-specific survival | |  |
| --- | --- | --- | --- | --- | --- | --- | --- | --- |
| Variant | Effect allele | HR*(95% CI) | P-value | Pfdr |  | HR*(95% CI) | P-value | Pfdr |
| *Stage II-III* |  |  |  |  |  |  |  |  |
| rs10161980 | G | 1.13(1.03-1.22) | 0.006 | 0.236 |  | 1.10(0.99-1.22) | 0.075 | 0.786 |
| rs10951878 | T | 0.92(0.85-1.00) | 0.047 | 0.516 |  | 0.91(0.82-1.01) | 0.068 | 0.786 |
| rs11196171 | G | 0.90(0.81-1.00) | 0.048 | 0.516 |  | 0.93(0.82-1.05) | 0.247 | 0.805 |
| rs1570405 | A | 1.09(1.00-1.19) | 0.040 | 0.516 |  | 1.14(1.02-1.27) | 0.020 | 0.520 |
| rs17816465 | A | 1.11(1.00-1.22) | 0.048 | 0.516 |  | 1.10(0.97-1.25) | 0.125 | 0.786 |
| rs1800469 | G | 1.13(1.03-1.24) | 0.009 | 0.236 |  | 1.15(1.02-1.29) | 0.019 | 0.520 |
| rs2696839 | C | 0.94(0.87-1.02) | 0.160 | 0.798 |  | 0.90(0.81-0.99) | 0.036 | 0.782 |
| rs3087967 | C | 0.90(0.83-0.98) | 0.021 | 0.452 |  | 0.86(0.78-0.96) | 0.005 | 0.520 |
| rs3131043 | G | 1.09(1.00-1.19) | 0.047 | 0.516 |  | 1.09(0.99-1.22) | 0.091 | 0.786 |
| rs4759277 | A | 1.08(0.99-1.17) | 0.084 | 0.725 |  | 1.11(1.00-1.23) | 0.044 | 0.786 |
| rs6065668 | T | 0.88(0.80-0.96) | 0.006 | 0.236 |  | 0.86(0.77-0.97) | 0.013 | 0.520 |
| rs847208 | A | 0.89(0.82-0.97) | 0.008 | 0.236 |  | 0.87(0.79-0.97) | 0.010 | 0.520 |
| rs9537521 | A | 1.13(1.04-1.23) | 0.005 | 0.236 |  | 1.09(0.98-1.22) | 0.100 | 0.786 |
| rs983318 | A | 1.11(1.01-1.22) | 0.036 | 0.516 |  | 1.11(0.99-1.25) | 0.083 | 0.786 |
|  |  |  |  |  |  |  |  |  |
| *Stage IV* |  |  |  |  |  |  |  |  |
| rs10994860 | T | 1.22(1.04-1.43) | 0.015 | 0.639 |  | 1.21(1.03-1.43) | 0.019 | 0.703 |
| rs12143541 | G | 1.18(1.01-1.37) | 0.042 | 0.639 |  | 1.18(1.01-1.38) | 0.040 | 0.703 |
| rs2238126 | G | 0.85(0.72-1.00) | 0.045 | 0.639 |  | 0.85(0.72-1.00) | 0.057 | 0.703 |
| rs3217810 | T | 1.20(0.99-1.46) | 0.057 | 0.639 |  | 1.23(1.01-1.50) | 0.035 | 0.703 |
| rs3801081 | G | 0.87(0.77-0.99) | 0.028 | 0.639 |  | 0.88(0.77-0.99) | 0.039 | 0.703 |
| rs3987 | G | 1.12(0.99-1.27) | 0.067 | 0.639 |  | 1.14(1.00-1.30) | 0.042 | 0.703 |
| rs72647484 | C | 0.78(0.63-0.96) | 0.021 | 0.639 |  | 0.76(0.61-0.95) | 0.016 | 0.703 |
| rs73208120 | G | 1.23(1.01-1.50) | 0.036 | 0.639 |  | 1.22(1.00-1.49) | 0.053 | 0.703 |
| rs847208 | A | 0.88(0.77-1.00) | 0.045 | 0.639 |  | 0.88(0.78-1.01) | 0.066 | 0.703 |

*hazard ratios are derived from Cox regression models adjusted for age at diagnosis and sex.
HR, hazard ratio; CI, confidence interval; Pfdr, p values after correction for false positive rates; CRC, colorectal cancer

Table S3 summarized results of association between variants at nominal significance (p<0.05) and CRC survival stratified by sex

|  |  | Overall survival |  |  |  | CRC-specific survival | |  |
| --- | --- | --- | --- | --- | --- | --- | --- | --- |
| Variant | Effect allele | HR*(95% CI) | P-value | Pfdr |  | HR*(95% CI) | P-value | Pfdr |
|  |  |  |  |  |  |  |  |  |
| *Male* |  |  |  |  |  |  |  |  |
| rs10994860 | T | 1.11(0.99-1.23) | 0.063 | 0.643 |  | 1.14(1.00-1.30) | 0.044 | 0.791 |
| rs11196171 | G | 0.88(0.79-0.98) | 0.018 | 0.592 |  | 0.90(0.80-1.03) | 0.117 | 0.826 |
| rs12143541 | G | 1.17(1.04-1.31) | 0.009 | 0.592 |  | 1.14(1.00-1.31) | 0.057 | 0.791 |
| rs17836917 | A | 1.35(1.01-1.80) | 0.045 | 0.629 |  | 1.38(0.98-1.95) | 0.069 | 0.791 |
| rs34405347 | G | 0.81(0.69-0.96) | 0.015 | 0.592 |  | 0.85(0.69-1.03) | 0.094 | 0.813 |
| rs35509282 | A | 0.94(0.82-1.07) | 0.315 | 0.740 |  | 0.85(0.73-1.00) | 0.047 | 0.791 |
| rs4776316 | G | 0.90(0.82-1.00) | 0.043 | 0.629 |  | 0.92(0.82-1.04) | 0.170 | 0.826 |
| rs4811050 | A | 1.08(0.98-1.20) | 0.128 | 0.653 |  | 1.13(1.00-1.28) | 0.049 | 0.791 |
| rs5934683 | C | 0.93(0.87-1.00) | 0.045 | 0.629 |  | 0.94(0.87-1.02) | 0.149 | 0.826 |
| rs647161 | A | 1.11(1.01-1.21) | 0.023 | 0.592 |  | 1.13(1.02-1.26) | 0.019 | 0.791 |
| rs7495132 | T | 1.08(0.95-1.22) | 0.227 | 0.740 |  | 1.21(1.05-1.40) | 0.008 | 0.791 |
| rs7993934 | T | 0.96(0.88-1.04) | 0.304 | 0.740 |  | 0.89(0.80-0.99) | 0.033 | 0.791 |
| rs961253 | A | 0.90(0.83-0.98) | 0.021 | 0.592 |  | 0.92(0.83-1.02) | 0.125 | 0.826 |
| rs9831861 | A | 1.09(1.00-1.19) | 0.046 | 0.629 |  | 1.07(0.97-1.18) | 0.183 | 0.826 |
|  |  |  |  |  |  |  |  |  |
| *Female* |  |  |  |  |  |  |  |  |
| rs10161980 | G | 1.15(1.03-1.27) | 0.010 | 0.417 |  | 1.16(1.02-1.31) | 0.019 | 0.397 |
| rs10980628 | C | 1.11(0.99-1.25) | 0.069 | 0.569 |  | 1.16(1.01-1.33) | 0.037 | 0.519 |
| rs13020391 | T | 1.09(0.98-1.21) | 0.116 | 0.660 |  | 1.17(1.04-1.33) | 0.012 | 0.397 |
| rs28488 | T | 0.89(0.80-1.00) | 0.047 | 0.569 |  | 0.86(0.76-0.98) | 0.021 | 0.397 |
| rs3087967 | C | 0.86(0.77-0.95) | 0.005 | 0.417 |  | 0.86(0.76-0.97) | 0.018 | 0.397 |
| rs3131043 | G | 1.13(1.02-1.26) | 0.018 | 0.459 |  | 1.18(1.04-1.33) | 0.008 | 0.397 |
| rs3217810 | T | 1.21(1.04-1.40) | 0.016 | 0.459 |  | 1.18(0.98-1.41) | 0.078 | 0.612 |
| rs3217874 | T | 1.12(1.01-1.24) | 0.036 | 0.569 |  | 1.10(0.97-1.24) | 0.130 | 0.612 |
| rs35107139 | C | 1.10(0.98-1.22) | 0.095 | 0.649 |  | 1.14(1.00-1.30) | 0.044 | 0.519 |
| rs4759277 | A | 1.06(0.96-1.18) | 0.272 | 0.754 |  | 1.13(1.00-1.28) | 0.048 | 0.520 |
| rs6065668 | T | 0.88(0.78-0.98) | 0.024 | 0.519 |  | 0.84(0.73-0.96) | 0.011 | 0.397 |
| rs72647484 | C | 0.84(0.70-1.00) | 0.055 | 0.569 |  | 0.80(0.64-0.99) | 0.041 | 0.519 |
| rs847208 | A | 0.91(0.82-1.01) | 0.079 | 0.569 |  | 0.86(0.77-0.98) | 0.018 | 0.397 |
| rs9537521 | A | 1.16(1.04-1.29) | 0.007 | 0.417 |  | 1.16(1.02-1.31) | 0.026 | 0.423 |
| rs9929218 | A | 0.89(0.79-0.99) | 0.038 | 0.569 |  | 0.93(0.81-1.06) | 0.253 | 0.734 |

*hazard ratios are derived from Cox regression models adjusted for age at diagnosis and AJCC stage.
HR, hazard ratio; CI, confidence interval; Pfdr, p values after correction for false positive rates; CRC, colorectal cancer

Table S4 summarized results of association between variants at nominal significance (p<0.05) and CRC survival stratified by tumor site

|  |  | Overall survival |  |  |  | CRC-specific survival | |  |
| --- | --- | --- | --- | --- | --- | --- | --- | --- |
| Variant | Effect allele | HR*(95% CI) | P-value | Pfdr |  | HR*(95% CI) | P-value | Pfdr |
|  |  |  |  |  |  |  |  |  |
| *Colon* |  |  |  |  |  |  |  |  |
| rs10849438 | G | 0.88(0.78-1.01) | 0.065 | 0.322 |  | 0.82(0.70-0.97) | 0.018 | 0.301 |
| rs10994860 | T | 1.16(1.05-1.29) | 0.005 | 0.120 |  | 1.20(1.06-1.37) | 0.004 | 0.225 |
| rs11196171 | G | 0.86(0.77-0.96) | 0.006 | 0.120 |  | 0.88(0.77-1.00) | 0.048 | 0.371 |
| rs11610543 | G | 1.09(1.00-1.18) | 0.048 | 0.310 |  | 1.08(0.98-1.19) | 0.132 | 0.465 |
| rs11893063 | A | 0.92(0.85-1.00) | 0.051 | 0.314 |  | 0.90(0.81-0.99) | 0.030 | 0.348 |
| rs12143541 | G | 1.14(1.02-1.28) | 0.027 | 0.231 |  | 1.15(1.00-1.31) | 0.045 | 0.371 |
| rs16959063 | A | 0.55(0.35-0.87) | 0.010 | 0.125 |  | 0.54(0.32-0.93) | 0.025 | 0.328 |
| rs174537 | T | 1.13(1.04-1.24) | 0.006 | 0.120 |  | 1.10(0.99-1.23) | 0.069 | 0.417 |
| rs2696839 | C | 0.89(0.82-0.96) | 0.005 | 0.120 |  | 0.87(0.79-0.96) | 0.006 | 0.225 |
| rs3087967 | C | 0.88(0.81-0.97) | 0.006 | 0.120 |  | 0.86(0.78-0.96) | 0.006 | 0.225 |
| rs3184504 | C | 0.92(0.84-1.00) | 0.045 | 0.306 |  | 0.94(0.85-1.04) | 0.238 | 0.640 |
| rs3217810 | T | 1.18(1.04-1.35) | 0.012 | 0.125 |  | 1.20(1.03-1.41) | 0.020 | 0.301 |
| rs3217874 | T | 1.12(1.03-1.22) | 0.008 | 0.125 |  | 1.11(1.01-1.23) | 0.034 | 0.348 |
| rs34405347 | G | 0.77(0.65-0.92) | 0.005 | 0.120 |  | 0.83(0.67-1.02) | 0.071 | 0.417 |
| rs35107139 | C | 1.12(1.02-1.23) | 0.015 | 0.153 |  | 1.10(0.99-1.23) | 0.077 | 0.417 |
| rs35509282 | A | 0.93(0.82-1.07) | 0.310 | 0.758 |  | 0.84(0.72-0.99) | 0.038 | 0.348 |
| rs3801081 | G | 0.89(0.81-0.97) | 0.011 | 0.125 |  | 0.88(0.79-0.97) | 0.015 | 0.301 |
| rs4759277 | A | 1.10(1.01-1.19) | 0.034 | 0.259 |  | 1.15(1.04-1.27) | 0.007 | 0.225 |
| rs4919687 | A | 1.10(1.00-1.21) | 0.040 | 0.288 |  | 1.07(0.96-1.19) | 0.238 | 0.640 |
| rs6065668 | T | 0.87(0.79-0.96) | 0.005 | 0.120 |  | 0.88(0.78-0.98) | 0.021 | 0.301 |
| rs6066825 | G | 1.07(0.98-1.17) | 0.135 | 0.551 |  | 1.12(1.01-1.24) | 0.036 | 0.348 |
| rs7495132 | T | 1.03(0.91-1.17) | 0.651 | 0.999 |  | 1.16(1.00-1.33) | 0.048 | 0.371 |
| rs847208 | A | 0.89(0.82-0.97) | 0.011 | 0.125 |  | 0.88(0.79-0.97) | 0.011 | 0.285 |
| rs9537521 | A | 1.10(1.01-1.21) | 0.031 | 0.252 |  | 1.06(0.95-1.18) | 0.299 | 0.712 |
| rs9929218 | A | 0.90(0.82-0.99) | 0.024 | 0.222 |  | 0.91(0.82-1.02) | 0.107 | 0.417 |
|  |  |  |  |  |  |  |  |  |
| *Rectum* |  |  |  |  |  |  |  |  |
| rs16969681 | T | 1.12(0.95-1.32) | 0.167 | 0.868 |  | 1.22(1.02-1.47) | 0.034 | 0.994 |
| rs2238126 | G | 0.85(0.74-0.98) | 0.026 | 0.868 |  | 0.82(0.69-0.97) | 0.020 | 0.994 |
| rs2279290 | G | 0.84(0.74-0.96) | 0.008 | 0.834 |  | 0.81(0.69-0.95) | 0.009 | 0.994 |
| rs7593422 | T | 0.88(0.80-0.97) | 0.013 | 0.834 |  | 0.89(0.79-1.00) | 0.052 | 0.994 |
| rs7993934 | T | 0.90(0.81-1.00) | 0.062 | 0.868 |  | 0.87(0.77-0.99) | 0.031 | 0.994 |

*hazard ratios are derived from Cox regression models adjusted for age at diagnosis, sex and AJCC stage.
HR, hazard ratio; CI, confidence interval; Pfdr, p values after correction for false positive rates; CRC, colorectal cancer

Table S5 summarized results of model performance using LASSO regression to predict survival outcomes

| Predictors |  | Model Performance |  |
| --- | --- | --- | --- |
|  |  | C-statistic (95% CI) | p-value* |
| *Overall survival* |  |  |  |
| Model 1^&^ |  |  |  |
| Age, sex, AJCC stage |  | 0.73277(0.69159-0.77396) | 0.678 |
| Model 2^&^ |  |  |  |
| Age, sex, AJCC stage, 6 variants ** |  | 0.73282(0.69161-0.77404) |  |
|  |  |  |  |
| *CRC-specific survival* |  |  |  |
| Model 1^&^ |  |  |  |
| Sex, AJCC stage |  | 0.77302(0.72908-0.81695) | 0.287 |
| Model 2^&^ |  |  |  |
| Sex, AJCC stage, rs35107139 |  | 0.76278(0.71885-0.80672) |  |

*p-values of U-statistic test denote the probability that model 1 is more concordant than model 2(p<0.05 is considered as significant incremental discriminative ability added by genetic variants)
& model 1 is the simplest model from cross-validations within 1 standard deviation of the penalty coefficient with the least prediction error; and model 2 is the model with the least prediction error
** rs10994860, rs34405347, rs35107139, rs6065668, rs9537521, rs9831861
 CI, confidence interval; CRC, colorectal cancer, AJCC, the American Joint Committee on Cancer.

**Reference**

1. Huyghe JR, Bien SA, Harrison TA, Kang HM, Chen S, Schmit SL, Conti DV, Qu C, Jeon J, Edlund CK, Greenside P, Wainberg M, et al. Discovery of common and rare genetic risk variants for colorectal cancer. *Nat Genet* 2019;**51**: 76-87.

2. Law PJ, Timofeeva M, Fernandez-Rozadilla C, Broderick P, Studd J, Fernandez-Tajes J, Farrington S, Svinti V, Palles C, Orlando G, Sud A, Holroyd A, et al. Association analyses identify 31 new risk loci for colorectal cancer susceptibility. *Nat Commun* 2019;**10**: 2154.

3. Schmit SL, Edlund CK, Schumacher FR, Gong J, Harrison TA, Huyghe JR, Qu C, Melas M, Van Den Berg DJ, Wang H, Tring S, Plummer SJ, et al. Novel Common Genetic Susceptibility Loci for Colorectal Cancer. *J Natl Cancer Inst* 2018.

4. Whiffin N, Hosking FJ, Farrington SM, Palles C, Dobbins SE, Zgaga L, Lloyd A, Kinnersley B, Gorman M, Tenesa A, Broderick P, Wang Y, et al. Identification of susceptibility loci for colorectal cancer in a genome-wide meta-analysis. *Hum Mol Genet* 2014;**23**: 4729-37.

5. Study C, Houlston RS, Webb E, Broderick P, Pittman AM, Di Bernardo MC, Lubbe S, Chandler I, Vijayakrishnan J, Sullivan K, Penegar S, Colorectal Cancer Association Study C, et al. Meta-analysis of genome-wide association data identifies four new susceptibility loci for colorectal cancer. *Nat Genet* 2008;**40**: 1426-35.

6. Jia WH, Zhang B, Matsuo K, Shin A, Xiang YB, Jee SH, Kim DH, Ren Z, Cai Q, Long J, Shi J, Wen W, et al. Genome-wide association analyses in East Asians identify new susceptibility loci for colorectal cancer. *Nat Genet* 2013;**45**: 191-6.

7. Tomlinson IP, Webb E, Carvajal-Carmona L, Broderick P, Howarth K, Pittman AM, Spain S, Lubbe S, Walther A, Sullivan K, Jaeger E, Fielding S, et al. A genome-wide association study identifies colorectal cancer susceptibility loci on chromosomes 10p14 and 8q23.3. *Nat Genet* 2008;**40**: 623-30.

8. Zhang B, Jia WH, Matsuda K, Kweon SS, Matsuo K, Xiang YB, Shin A, Jee SH, Kim DH, Cai Q, Long J, Shi J, et al. Large-scale genetic study in East Asians identifies six new loci associated with colorectal cancer risk. *Nat Genet* 2014;**46**: 533-42.

9. Houlston RS, Cheadle J, Dobbins SE, Tenesa A, Jones AM, Howarth K, Spain SL, Broderick P, Domingo E, Farrington S, Prendergast JG, Pittman AM, et al. Meta-analysis of three genome-wide association studies identifies susceptibility loci for colorectal cancer at 1q41, 3q26.2, 12q13.13 and 20q13.33. *Nat Genet* 2010;**42**: 973-7.

10. Zeng C, Matsuda K, Jia WH, Chang J, Kweon SS, Xiang YB, Shin A, Jee SH, Kim DH, Zhang B, Cai Q, Guo X, et al. Identification of Susceptibility Loci and Genes for Colorectal Cancer Risk. *Gastroenterology* 2016;**150**: 1633-45.

11. Tomlinson IP, Carvajal-Carmona LG, Dobbins SE, Tenesa A, Jones AM, Howarth K, Palles C, Broderick P, Jaeger EE, Farrington S, Lewis A, Prendergast JG, et al. Multiple common susceptibility variants near BMP pathway loci GREM1, BMP4, and BMP2 explain part of the missing heritability of colorectal cancer. *PLoS Genet* 2011;**7**: e1002105.

12. Peters U, Jiao S, Schumacher FR, Hutter CM, Aragaki AK, Baron JA, Berndt SI, Bezieau S, Brenner H, Butterbach K, Caan BJ, Campbell PT, et al. Identification of Genetic Susceptibility Loci for Colorectal Tumors in a Genome-Wide Meta-analysis. *Gastroenterology* 2013;**144**: 799-807 e24.

13. Wang H, Burnett T, Kono S, Haiman CA, Iwasaki M, Wilkens LR, Loo LW, Van Den Berg D, Kolonel LN, Henderson BE, Keku TO, Sandler RS, et al. Trans-ethnic genome-wide association study of colorectal cancer identifies a new susceptibility locus in VTI1A. *Nat Commun* 2014;**5**: 4613.

14. Jiang K, Sun Y, Wang C, Ji J, Li Y, Ye Y, Lv L, Guo Y, Guo S, Li H, Zhang L, Zhou Y, et al. Genome-wide association study identifies two new susceptibility loci for colorectal cancer at 5q23.3 and 17q12 in Han Chinese. *Oncotarget* 2015;**6**: 40327-36.

15. Wang M, Gu D, Du M, Xu Z, Zhang S, Zhu L, Lu J, Zhang R, Xing J, Miao X, Chu H, Hu Z, et al. Common genetic variation in ETV6 is associated with colorectal cancer susceptibility. *Nat Commun* 2016;**7**: 11478.

16. Schumacher FR, Schmit SL, Jiao S, Edlund CK, Wang H, Zhang B, Hsu L, Huang SC, Fischer CP, Harju JF, Idos GE, Lejbkowicz F, et al. Genome-wide association study of colorectal cancer identifies six new susceptibility loci. *Nat Commun* 2015;**6**: 7138.

17. Schmit SL, Schumacher FR, Edlund CK, Conti DV, Raskin L, Lejbkowicz F, Pinchev M, Rennert HS, Jenkins MA, Hopper JL, Buchanan DD, Lindor NM, et al. A novel colorectal cancer risk locus at 4q32.2 identified from an international genome-wide association study. *Carcinogenesis* 2014;**35**: 2512-9.

18. Dunlop MG, Dobbins SE, Farrington SM, Jones AM, Palles C, Whiffin N, Tenesa A, Spain S, Broderick P, Ooi LY, Domingo E, Smillie C, et al. Common variation near CDKN1A, POLD3 and SHROOM2 influences colorectal cancer risk. *Nat Genet* 2012;**44**: 770-6.

19. Real LM, Ruiz A, Gayan J, Gonzalez-Perez A, Saez ME, Ramirez-Lorca R, Moron FJ, Velasco J, Marginet-Flinch R, Musulen E, Carrasco JM, Moreno-Rey C, et al. A colorectal cancer susceptibility new variant at 4q26 in the Spanish population identified by genome-wide association analysis. *PLoS One* 2014;**9**: e101178.

20. Tenesa A, Farrington SM, Prendergast JG, Porteous ME, Walker M, Haq N, Barnetson RA, Theodoratou E, Cetnarskyj R, Cartwright N, Semple C, Clark AJ, et al. Genome-wide association scan identifies a colorectal cancer susceptibility locus on 11q23 and replicates risk loci at 8q24 and 18q21. *Nat Genet* 2008;**40**: 631-7.

21. Tanikawa C, Kamatani Y, Takahashi A, Momozawa Y, Leveque K, Nagayama S, Mimori K, Mori M, Ishii H, Inazawa J, Yasuda J, Tsuboi A, et al. GWAS identifies two novel colorectal cancer loci at 16q24.1 and 20q13.12. *Carcinogenesis* 2018;**39**: 652-60.

22. Tomlinson I, Webb E, Carvajal-Carmona L, Broderick P, Kemp Z, Spain S, Penegar S, Chandler I, Gorman M, Wood W, Barclay E, Lubbe S, et al. A genome-wide association scan of tag SNPs identifies a susceptibility variant for colorectal cancer at 8q24.21. *Nat Genet* 2007;**39**: 984-8.

23. Al-Tassan NA, Whiffin N, Hosking FJ, Palles C, Farrington SM, Dobbins SE, Harris R, Gorman M, Tenesa A, Meyer BF, Wakil SM, Kinnersley B, et al. A new GWAS and meta-analysis with 1000Genomes imputation identifies novel risk variants for colorectal cancer. *Sci Rep* 2015;**5**: 10442.
