# Supplementary material for: Effects of common genetic variants associated with colorectal cancer risk on survival outcomes after diagnosis: A large population‐based cohort study
Source: Int J Cancer. 2019 Jul 27;145(9):2427–32. doi: 10.1002/ijc.32550 (PMC6771941; doi:10.1002/ijc.32550)
Supplement: Supplementary file 2 — Table S6 Functional annotation of analyzed genetic variants [file IJC-145-2427-s002.docx]

Table S6 Functional annotation of analyzed genetic variants

| _Variant_ | _Allele_ | _Chr_ | _Position (bp,GRCh37)_ | _Consequence type_ | _Cis expression Quantitative Loci (cis-eQTL) analysis results from the GTEx transevrse colon dataset_ | | | | _Regulome DB annotation and score_ | | _Pathogenicity predictions for amino acids substitutions_ | | | |
| --- | --- | --- | --- | --- | --- | --- | --- | --- | --- | --- | --- | --- | --- | --- |
|  |  |  |  |  | _eQTL Transcript Ensembl ID_ | _eQTL gene name_ | _P value_ | _Normilized effect size_ | _Hits (given for scores above 1f only)_ | _Score_ | _Polyphen_prediction_ | _Polyphen_score_ | _Sift_prediction_ | _Sift_score_ |
| _rs72647484_ | _T/C_ | _1_ | _22587728_ |  |  |  | _NA_ | _NA_ | _Motifs\|PWM\|\|Pit-1, Motifs\|PWM\|\|SOX9, Motifs\|Footprinting\|H1hesc\|Pit-1, Motifs\|Footprinting\|NHEK\|_ | _6_ |  | _NA_ |  | _NA_ |
| _rs61776719_ | _C/A_ | _1_ | _38461319_ |  | _ENSG00000183431.7_ | _SF3A3_ | _8.02E-07_ | _-0.19_ | _Chromatin_Structure\|\|DNase-seq\|Tregwb83319432, Chromatin_Structure\|\|DNase-seq\|Cd4naivewb11970640, Chromatin_Structure\|Ifng4h\|FAIRE\|Helas3, Chromatin_Structure\|\|DNase-seq\|Rptec, Chromatin_Structure\|\|DNase-seq\|Th17, Chromatin_Structure\|\|DNase-seq\|Th2, Chromatin_Structure\|\|DNase-seq\|Tregwb78495824, Chromatin_Structure\|\|DNase-seq\|Th2wb54553204, Chromatin_Structure\|Ifna4h\|FAIRE\|Helas3, Chromatin_Structure\|\|FAIRE\|Gm19239, Chromatin_Structure\|\|DNase-seq\|Hrce, Chromatin_Structure\|\|DNase-seq\|Hl60, Chromatin_Structure\|\|DNase-seq\|Nhek, Chromatin_Structure\|\|DNase-seq\|Th1_ | _5_ |  | _NA_ |  | _NA_ |
| **_rs12143541*_** | **_A/G_** | **_1_** | **_55247852_** | **_intron_variant_** | **_ENSG00000116209_** | **_TMEM59_** | **_0.0269_** | **_-0.09_** | **_Chromatin_Structure\|Est10nm30m\|DNase-seq\|Ishikawa, Chromatin_Structure\|\|DNase-seq\|Heartoc, Chromatin_Structure\|\|DNase-seq\|Lncap, Chromatin_Structure\|\|DNase-seq\|Cerebrumfrontaloc, Chromatin_Structure\|\|DNase-seq\|8988t, Chromatin_Structure\|\|DNase-seq\|Hsmm, Chromatin_Structure\|\|DNase-seq\|Panislets, Chromatin_Structure\|\|DNase-seq\|H1hesc, Chromatin_Structure\|Tam10030\|DNase-seq\|Ishikawa, Chromatin_Structure\|Andro\|DNase-seq\|Lncap, Chromatin_Structure\|\|DNase-seq\|Mcf7, Chromatin_Structure\|\|DNase-seq\|Hepatocytes, Chromatin_Structure\|\|DNase-seq\|Gm10266, Chromatin_Structure\|\|DNase-seq\|Frontalcortexoc, Chromatin_Structure\|\|DNase-seq\|T47d, Chromatin_Structure\|\|DNase-seq\|Gliobla, Chromatin_Structure\|\|DNase-seq\|Hsmmt, Chromatin_Structure\|\|DNase-seq\|Gm13977, Chromatin_Structure\|\|DNase-seq\|Medullo, Chromatin_Structure\|\|DNase-seq\|Chorion, Chromatin_Structure\|\|DNase-seq\|Myometr, Chromatin_Structure\|Dm002p1h\|DNase-seq\|Ecc1, Chromatin_Structure\|\|DNase-seq\|Psoasmuscleoc_** | **_5_** |  | **_NA_** |  | **_NA_** |
| _rs10911251_ | _A/C_ | _1_ | _183081194_ | _intron_variant_ | _NA_ | _NA_ | _NA_ | _NA_ | _Motifs\|PWM\|\|HNF3, Motifs\|PWM\|\|HFH4(FOXJ1), Motifs\|PWM\|\|FOXC1, Motifs\|PWM\|\|FOXfactors, Motifs\|PWM\|\|FOXJ3, Motifs\|PWM\|\|FOXP1, Motifs\|PWM\|\|Zfp105, Motifs\|PWM\|\|FOXD3, Motifs\|PWM\|\|FOXI1, Motifs\|PWM\|\|Foxd3, Motifs\|PWM\|\|FOXJ2, Motifs\|PWM\|\|HFH3(FOXI1)_ | _6_ |  | _NA_ |  | _NA_ |
| _rs6658977_ | _G/T_ | _1_ | _222049820_ |  |  |  | _NA_ | _NA_ | _No data_ | _7_ |  | _NA_ |  | _NA_ |
| _rs11692435_ | _G/A_ | _2_ | _98275354_ | _missense_variant_ | _ENSG00000115073.6_ | _ACTR1B_ | _1.40E-18_ | _0.8_ | _Chromatin_Structure\|\|DNase-seq\|Lncap, Chromatin_Structure\|\|DNase-seq\|Adultcd4th0, Chromatin_Structure\|\|DNase-seq\|Hmec, Chromatin_Structure\|\|DNase-seq\|Th1, Chromatin_Structure\|\|DNase-seq\|Medullo, Chromatin_Structure\|\|DNase-seq\|Gm12892, Protein_Binding\|\|ChIP-seq\|HEK293-T-REx\|ZNF263, Protein_Binding\|forskolin\|ChIP-seq\|HepG2\|POLR2A_ | _4_ | _probably damaging_ | _0.999_ | _deleterious_ | _0_ |
| _rs448513_ | _T/C_ | _2_ | _159964552_ | _intron_variant_ | _NA_ | _NA_ | _NA_ | _NA_ | _Motifs\|PWM\|\|MYBL2_ | _6_ |  | _NA_ |  | _NA_ |
| _rs11903757_ | _T/C_ | _2_ | _192587204_ |  | _NA_ | _NA_ | _NA_ | _NA_ | _Motifs\|PWM\|\|Zfp740_ | _6_ |  | _NA_ |  | _NA_ |
| _rs11893063_ | _G/A_ | _2_ | _199601925_ | _intron_variant_ |  |  | _NA_ | _NA_ | _No data_ | _7_ |  | _NA_ |  | _NA_ |
| _rs7593422_ | _A/T_ | _2_ | _200131695_ |  |  |  | _NA_ | _NA_ | _Motifs\|PWM\|\|FAC1, Chromatin_Structure\|\|DNase-seq\|Hpdlf, Chromatin_Structure\|\|DNase-seq\|Hbvp_ | _5_ |  | _NA_ |  | _NA_ |
| _rs13020391_ | _C/T_ | _2_ | _219184436_ | _intron_variant_ | _ENSG00000261338_ | _RP11-378A13.1_ | _1.15E-26_ | _-0.74_ | _No data_ | _7_ |  | _NA_ |  | _NA_ |
| _rs35360328_ | _T/A_ | _3_ | _40924962_ |  | _NA_ | _NA_ | _NA_ | _NA_ | _Motifs\|PWM\|\|Cphx, Motifs\|PWM\|\|Arid3a, Motifs\|PWM\|\|DUXA_ | _6_ |  | _NA_ |  | _NA_ |
| _rs9831861_ | _T/G_ | _3_ | _53088285_ | _intron_variant_ | _ENSG00000163935_ | _SFMBT1_ | _2.36E-13_ | _0.38_ | _Chromatin_Structure\|\|DNase-seq\|Melano_ | _5_ |  | _NA_ |  | _NA_ |
| _rs2279290_ | _T/A/C/G_ | _3_ | _66430811_ | _missense_variant_ | _NA_ | _NA_ | _NA_ | _NA_ | _Chromatin_Structure\|\|DNase-seq\|Medullo_ | _5_ | _benign_ | _0.01_ | _deleterious - low confidence_ | _0.01_ |
| _rs12635946_ | _C/T_ | _3_ | _112916918_ |  |  |  | _NA_ | _NA_ | _No data_ | _7_ |  | _NA_ |  | _NA_ |
| _rs72942485_ | _G/A_ | _3_ | _112999560_ | _intron_variant_ | _NA_ | _NA_ | _NA_ | _NA_ | _Chromatin_Structure\|\|DNase-seq\|Chorion_ | _5_ |  | _NA_ |  | _NA_ |
| _rs10049390_ | _G/A_ | _3_ | _133701119_ | _intron_variant_ | _NA_ | _NA_ | _NA_ | _NA_ | _No data_ | _7_ |  | _NA_ |  | _NA_ |
| _rs10936599_ | _C/T_ | _3_ | _169492101_ | _synonymous_variant_ | _NA_ | _NA_ | _NA_ | _NA_ | _Motifs\|PWM\|\|NRSF, Motifs\|PWM\|\|NRSE, Chromatin_Structure\|\|DNase-seq\|Hcpe, Chromatin_Structure\|\|DNase-seq\|Rptec, Chromatin_Structure\|\|DNase-seq\|Hmvecdad, Chromatin_Structure\|\|DNase-seq\|Th17, Chromatin_Structure\|\|DNase-seq\|Hmvecdlyneo, Chromatin_Structure\|\|FAIRE\|Gm18507, Chromatin_Structure\|\|DNase-seq\|Hmvecdblneo, Chromatin_Structure\|\|DNase-seq\|H7es, Chromatin_Structure\|\|DNase-seq\|Saec, Chromatin_Structure\|\|DNase-seq\|Hmvecdblad, Chromatin_Structure\|\|FAIRE\|Gm19239, Chromatin_Structure\|\|DNase-seq\|Hrce, Chromatin_Structure\|\|DNase-seq\|Mcf7, Chromatin_Structure\|\|DNase-seq\|Hee, Chromatin_Structure\|Hypoxlac\|DNase-seq\|Mcf7, Chromatin_Structure\|Hypoxlac\|FAIRE\|Mcf7, Chromatin_Structure\|\|DNase-seq\|Th1_ | _5_ |  | _NA_ |  | _NA_ |
| _rs1370821_ | _C/T_ | _4_ | _94943383_ |  | _NA_ | _NA_ | _NA_ | _NA_ | _Protein_Binding\|\|ChIP-seq\|K562\|RFX3_ | _5_ |  | _NA_ |  | _NA_ |
| _rs17035289_ | _T/C_ | _4_ | _106048291_ |  | _ENSG00000168743_ | _NPNT_ | _0.016_ | _0.15_ | _Motifs\|PWM\|\|BARHL1, Motifs\|PWM\|\|EN1, Motifs\|PWM\|\|BARX1, Motifs\|PWM\|\|LHX9, Motifs\|PWM\|\|BARHL2, Motifs\|PWM\|\|MSX1, Chromatin_Structure\|\|FAIRE\|Panislets, Chromatin_Structure\|\|FAIRE\|Gm12891, Chromatin_Structure\|\|DNase-seq\|Gm12878_ | _5_ |  | _NA_ |  | _NA_ |
| _rs1391441_ | _G/A_ | _4_ | _106128760_ | _intron_variant_ | _NA_ | _NA_ | _NA_ | _NA_ | _Chromatin_Structure\|Est100nm1h\|DNase-seq\|Mcf7, Chromatin_Structure\|\|DNase-seq\|Lncap, Chromatin_Structure\|\|FAIRE\|Gm12892, Chromatin_Structure\|Estctrl0h\|DNase-seq\|Mcf7, Chromatin_Structure\|\|DNase-seq\|Mcf7, Chromatin_Structure\|\|DNase-seq\|T47d, Chromatin_Structure\|\|DNase-seq\|Hee, Protein_Binding\|\|ChIP-seq\|K562\|RFX3_ | _4_ |  | _NA_ |  | _NA_ |
| _rs3987_ | _A/G_ | _4_ | _118759055_ | _intron_variant_ | _NA_ | _NA_ | _NA_ | _NA_ | _Motifs\|PWM\|\|Sox11_ | _6_ |  | _NA_ |  | _NA_ |
| _rs75686861_ | _G/A_ | _4_ | _145621328_ | _intron_variant_ |  |  | _NA_ | _NA_ | _Motifs\|PWM\|\|BLIMP1, Chromatin_Structure\|\|FAIRE\|Medullo, Chromatin_Structure\|\|DNase-seq\|Hcm_ | _5_ |  | _NA_ |  | _NA_ |
| **_rs35509282*_** | **_T/A_** | **_4_** | **_163333405_** |  | **_NA_** | **_NA_** | **_NA_** | **_NA_** | **_No data_** | **_7_** |  | **_NA_** |  | **_NA_** |
| _rs186722897_ | _A/T_ | _4_ | _163374639_ |  | _NA_ | _NA_ | _NA_ | _NA_ | _Motifs\|PWM\|\|Zfp128, Motifs\|PWM\|\|Six6_ | _6_ |  | _NA_ |  | _NA_ |
| _rs77776598_ | _T/C_ | _5_ | _1240998_ | _intron_variant_ | _ENSG00000188818_ | _ZDHHC11_ | _0.0135_ | _0.39_ | _Chromatin_Structure\|\|DNase-seq\|UrotsaUt189, Chromatin_Structure\|\|DNase-seq\|Lncap, Chromatin_Structure\|\|DNase-seq\|8988t, Chromatin_Structure\|\|DNase-seq\|H7es, Chromatin_Structure\|\|DNase-seq\|Hsmm, Chromatin_Structure\|\|DNase-seq\|Monocd14, Chromatin_Structure\|\|DNase-seq\|K562, Chromatin_Structure\|Saha1u72hr\|DNase-seq\|K562, Chromatin_Structure\|\|DNase-seq\|Hepatocytes, Chromatin_Structure\|\|DNase-seq\|T47d, Chromatin_Structure\|\|DNase-seq\|Helas3, Chromatin_Structure\|Ifna4h\|DNase-seq\|Helas3, Chromatin_Structure\|\|DNase-seq\|Urotsa, Chromatin_Structure\|\|DNase-seq\|Gm13977, Chromatin_Structure\|\|DNase-seq\|Chorion, Chromatin_Structure\|\|DNase-seq\|Gm12892, Chromatin_Structure\|\|DNase-seq\|Psoasmuscleoc_ | _5_ |  | _NA_ |  | _NA_ |
| _rs2735940_ | _A/G_ | _5_ | _1296486_ |  |  |  | _NA_ | _NA_ | _Chromatin_Structure\|\|DNase-seq\|Medullod341, Chromatin_Structure\|\|DNase-seq\|H7es, Chromatin_Structure\|\|DNase-seq\|Hsmm, Chromatin_Structure\|\|DNase-seq\|Cd4naivewb78495824, Chromatin_Structure\|\|DNase-seq\|H1hesc, Chromatin_Structure\|Ifna4h\|FAIRE\|Helas3, Chromatin_Structure\|\|DNase-seq\|Werirb1, Chromatin_Structure\|\|DNase-seq\|Mcf7, Chromatin_Structure\|\|DNase-seq\|T47d, Chromatin_Structure\|\|DNase-seq\|Huh7, Chromatin_Structure\|\|DNase-seq\|Helas3, Chromatin_Structure\|Ifna4h\|DNase-seq\|Helas3, Chromatin_Structure\|Est10nm30m\|DNase-seq\|T47d, Chromatin_Structure\|\|DNase-seq\|Huh75, Chromatin_Structure\|\|DNase-seq\|Medullo_ | _5_ |  | _NA_ |  | _NA_ |
| _rs7708610_ | _G/A/C_ | _5_ | _40102443_ |  | _NA_ | _NA_ | _NA_ | _NA_ | _Motifs\|PWM\|\|Zic1, Motifs\|PWM\|\|Zic2, Chromatin_Structure\|Ifng4h\|FAIRE\|Helas3, Chromatin_Structure\|\|FAIRE\|Htr8, Chromatin_Structure\|Ifna4h\|FAIRE\|Helas3_ | _6_ |  | _NA_ |  | _NA_ |
| _rs1445011_ | _T/C/G_ | _5_ | _40280202_ |  |  |  | _NA_ | _NA_ | _Chromatin_Structure\|\|DNase-seq\|Th17, Chromatin_Structure\|\|DNase-seq\|Th2, Chromatin_Structure\|\|DNase-seq\|Th2wb54553204, Chromatin_Structure\|\|DNase-seq\|Th1, Chromatin_Structure\|\|DNase-seq\|Nhdfad_ | _5_ |  | _NA_ |  | _NA_ |
| _rs12522693_ | _G/A_ | _5_ | _130195731_ |  | _NA_ | _NA_ | _NA_ | _NA_ | _No data_ | _7_ |  | _NA_ |  | _NA_ |
| _rs639933_ | _C/A_ | _5_ | _134467751_ | _intron_variant_ | _ENSG00000113621_ | _TXNDC15_ | _0.0463_ | _-0.06_ | _Chromatin_Structure\|\|DNase-seq\|Wi38, Chromatin_Structure\|\|DNase-seq\|Ag04450, Chromatin_Structure\|\|DNase-seq\|Fibropag08396, Chromatin_Structure\|\|DNase-seq\|Fibrobl, Chromatin_Structure\|\|DNase-seq\|Fibrop, Chromatin_Structure\|Ohtam\|DNase-seq\|Wi38, Chromatin_Structure\|Diff4d\|DNase-seq\|Lhcnm2, Chromatin_Structure\|\|DNase-seq\|Lhcnm2_ | _5_ |  | _NA_ |  | _NA_ |
| _rs647161_ | _C/A_ | _5_ | _134499092_ | _intron_variant_ | _NA_ | _NA_ | _NA_ | _NA_ | _Motifs\|PWM\|\|Zbtb7b, Chromatin_Structure\|\|DNase-seq\|Medullod341, Chromatin_Structure\|Ifna4h\|FAIRE\|Helas3, Chromatin_Structure\|\|DNase-seq\|Medullo_ | _5_ |  | _NA_ |  | _NA_ |
| _rs2070699_ | _G/C/T_ | _6_ | _12292772_ | _intron_variant_ | _ENSG00000112137_ | _PHACTR1_ | _0.0321_ | _0.17_ | _Motifs\|PWM\|\|Pax-2, Motifs\|PWM\|\|Emx2, Motifs\|Footprinting\|Gm19239\|Pax-2, Motifs\|Footprinting\|A549\|Pax-2, Motifs\|Footprinting\|Hsmmt\|Pax-2, Motifs\|Footprinting\|Hepatocytes\|Pax-2, Motifs\|Footprinting\|H9es\|Pax-2, Motifs\|Footprinting\|Htr8\|Pax-2, Motifs\|Footprinting\|Gm12878\|Pax-2, Motifs\|Footprinting\|Hpde6e6e7\|Pax-2, Motifs\|Footprinting\|Phte\|Pax-2, Motifs\|Footprinting\|Mcf7Hypoxlac\|Pax-2, Motifs\|Footprinting\|Hepg2\|Pax-2, Motifs\|Footprinting\|AosmcSerumfree\|Pax-2, Motifs\|Footprinting\|Mcf7\|Pax-2, Motifs\|Footprinting\|Huh75\|Pax-2, Motifs\|Footprinting\|Fibrop\|Pax-2, Motifs\|Footprinting\|Panisd\|Pax-2, Motifs\|Footprinting\|H1hesc\|Pax-2, Motifs\|Footprinting\|8988t\|Pax-2, Motifs\|Footprinting\|Myometr\|Pax-2, Motifs\|Footprinting\|Huh7\|Pax-2, Chromatin_Structure\|\|DNase-seq\|Progfib, Chromatin_Structure\|Diffa14d\|DNase-seq\|H7es, Chromatin_Structure\|\|DNase-seq\|Ipsnihi7, Chromatin_Structure\|\|DNase-seq\|Htr8, Chromatin_Structure\|\|DNase-seq\|Hpdlf, Chromatin_Structure\|\|DNase-seq\|Hmvecdneo, Chromatin_Structure\|\|DNase-seq\|Fibropag08395, Chromatin_Structure\|\|DNase-seq\|Hrpe, Chromatin_Structure\|Znfe103c6\|DNase-seq\|K562, Chromatin_Structure\|\|DNase-seq\|Heartoc, Chromatin_Structure\|\|DNase-seq\|Hcf, Chromatin_Structure\|\|DNase-seq\|Ag09319, Chromatin_Structure\|Est100nm1h\|DNase-seq\|Mcf7, Chromatin_Structure\|\|DNase-seq\|Hcpe, Chromatin_Structure\|\|DNase-seq\|Ag10803, Chromatin_Structure\|Hypoxlaccon\|DNase-seq\|Mcf7, Chromatin_Structure\|\|DNase-seq\|Rptec, Chromatin_Structure\|\|DNase-seq\|Gm12864, Chromatin_Structure\|\|DNase-seq\|M059j, Chromatin_Structure\|\|DNase-seq\|Lncap, Chromatin_Structure\|\|DNase-seq\|Nt2d1, Chromatin_Structure\|\|DNase-seq\|Cd34mobilized, Chromatin_Structure\|\|DNase-seq\|Gm04504, Chromatin_Structure\|\|FAIRE\|Nhek, Chromatin_Structure\|\|DNase-seq\|Hcfaa, Chromatin_Structure\|\|DNase-seq\|8988t, Chromatin_Structure\|\|DNase-seq\|Hmveclbl, Chromatin_Structure\|\|DNase-seq\|Hah, Chromatin_Structure\|\|DNase-seq\|Gm19240, Chromatin_Structure\|\|DNase-seq\|Melano, Chromatin_Structure\|\|DNase-seq\|Wi38, Chromatin_Structure\|\|DNase-seq\|Hvmf, Chromatin_Structure\|\|DNase-seq\|Hrgec, Chromatin_Structure\|\|DNase-seq\|Hffmyc, Chromatin_Structure\|Ctcfshrna\|DNase-seq\|Mcf7, Chromatin_Structure\|\|DNase-seq\|Gm10248, Chromatin_Structure\|\|DNase-seq\|Hmvecdlyneo, Chromatin_Structure\|\|DNase-seq\|Hae, Chromatin_Structure\|\|DNase-seq\|Ipscwru1, Chromatin_Structure\|Estctrl0h\|DNase-seq\|Mcf7, Chromatin_Structure\|\|DNase-seq\|Gm20000, Chromatin_Structure\|\|DNase-seq\|H7es, Chromatin_Structure\|\|DNase-seq\|Hct116, Chromatin_Structure\|\|DNase-seq\|Hsmm, Chromatin_Structure\|\|DNase-seq\|Hac, Chromatin_Structure\|\|DNase-seq\|Hmf, Chromatin_Structure\|\|DNase-seq\|Hpaf, Chromatin_Structure\|\|DNase-seq\|Skmc, Chromatin_Structure\|\|DNase-seq\|Hasp, Chromatin_Structure\|\|DNase-seq\|Gm13976, Chromatin_Structure\|\|DNase-seq\|Panislets, Chromatin_Structure\|Znfa41c6\|DNase-seq\|K562, Chromatin_Structure\|\|DNase-seq\|H1hesc, Chromatin_Structure\|\|DNase-seq\|Fibrobl, Chromatin_Structure\|\|DNase-seq\|Hmvecdblad, Chromatin_Structure\|\|DNase-seq\|Nhbera, Chromatin_Structure\|\|DNase-seq\|Hipe, Chromatin_Structure\|Diffa5d\|DNase-seq\|H7es, Chromatin_Structure\|\|DNase-seq\|Hff, Chromatin_Structure\|\|DNase-seq\|K562, Chromatin_Structure\|Diffa9d\|DNase-seq\|H7es, Chromatin_Structure\|\|DNase-seq\|Werirb1, Chromatin_Structure\|\|DNase-seq\|Hnpce, Chromatin_Structure\|\|DNase-seq\|Hrce, Chromatin_Structure\|\|DNase-seq\|Ips, Chromatin_Structure\|Diffa2d\|DNase-seq\|H7es, Chromatin_Structure\|\|DNase-seq\|Mcf7, Chromatin_Structure\|\|DNase-seq\|Fibrop, Chromatin_Structure\|\|DNase-seq\|Hepatocytes, Chromatin_Structure\|\|DNase-seq\|T47d, Chromatin_Structure\|\|DNase-seq\|A549, Chromatin_Structure\|\|DNase-seq\|Huh7, Chromatin_Structure\|\|DNase-seq\|Gm12865, Chromatin_Structure\|\|DNase-seq\|Hgf, Chromatin_Structure\|\|DNase-seq\|Hsmmt, Chromatin_Structure\|\|DNase-seq\|Hbvsmc, Chromatin_Structure\|Znfp5\|DNase-seq\|K562, Chromatin_Structure\|\|DNase-seq\|Nha, Chromatin_Structure\|Est10nm30m\|DNase-seq\|T47d, Chromatin_Structure\|\|DNase-seq\|Huh75, Chromatin_Structure\|\|DNase-seq\|Gm19239, Chromatin_Structure\|\|DNase-seq\|Gm04503, Chromatin_Structure\|\|DNase-seq\|Hre, Chromatin_Structure\|\|DNase-seq\|Hmec, Chromatin_Structure\|\|DNase-seq\|Nhdfneo, Chromatin_Structure\|\|DNase-seq\|Phte, Chromatin_Structure\|\|DNase-seq\|Hcm, Chromatin_Structure\|\|DNase-seq\|Hmvecdlyad, Chromatin_Structure\|\|DNase-seq\|H9es, Chromatin_Structure\|\|DNase-seq\|Gm13977, Chromatin_Structure\|Diff4d\|DNase-seq\|Lhcnm2, Chromatin_Structure\|Ohtam\|DNase-seq\|Wi38, Chromatin_Structure\|\|DNase-seq\|Hepg2, Chromatin_Structure\|\|DNase-seq\|Hmveclly, Chromatin_Structure\|Hypoxlac\|DNase-seq\|Mcf7, Chromatin_Structure\|\|DNase-seq\|Bj, Chromatin_Structure\|Hypoxlac\|FAIRE\|Mcf7, Chromatin_Structure\|\|DNase-seq\|Lhcnm2, Chromatin_Structure\|\|DNase-seq\|Ag04449, Chromatin_Structure\|\|DNase-seq\|Nhlf, Chromatin_Structure\|\|DNase-seq\|Medullo, Chromatin_Structure\|\|DNase-seq\|Panc1, Chromatin_Structure\|\|DNase-seq\|Hbmec, Chromatin_Structure\|\|DNase-seq\|Gm18507, Chromatin_Structure\|\|DNase-seq\|Hs27a, Chromatin_Structure\|\|DNase-seq\|Gm12878, Chromatin_Structure\|Randshrna\|DNase-seq\|Mcf7, Chromatin_Structure\|\|DNase-seq\|Gm06990, Chromatin_Structure\|\|DNase-seq\|Nhdfad, Chromatin_Structure\|\|FAIRE\|Hepg2, Chromatin_Structure\|\|DNase-seq\|Aoaf, Chromatin_Structure\|\|DNase-seq\|Rpmi7951, Protein_Binding\|\|ChIP-seq\|SK-N-SH\|RAD21, Protein_Binding\|\|ChIP-seq\|WI-38\|CTCF, Protein_Binding\|\|ChIP-seq\|GM12864\|CTCF, Protein_Binding\|\|ChIP-seq\|HCT-116\|CTCF, Protein_Binding\|02pct\|ChIP-seq\|A549\|BCL3, Protein_Binding\|\|ChIP-seq\|AoAF\|CTCF, Protein_Binding\|\|ChIP-seq\|AG04449\|CTCF, Protein_Binding\|\|ChIP-seq\|A549\|POLR2A, Protein_Binding\|\|ChIP-seq\|SAEC\|CTCF, Protein_Binding\|\|ChIP-seq\|Fibrobl\|CTCF, Protein_Binding\|\|ChIP-seq\|HeLa-S3\|SMC3, Protein_Binding\|\|ChIP-seq\|GM06990\|CTCF, Protein_Binding\|\|ChIP-seq\|IMR90\|RAD21, Protein_Binding\|dex_100nm\|ChIP-seq\|A549\|POLR2A, Protein_Binding\|\|ChIP-seq\|HCM\|CTCF, Protein_Binding\|\|ChIP-seq\|AG10803\|CTCF, Protein_Binding\|\|ChIP-seq\|GM12873\|CTCF, Protein_Binding\|\|ChIP-seq\|GM12878\|RAD21, Protein_Binding\|\|ChIP-seq\|H1-hESC\|RAD21, Protein_Binding\|\|ChIP-seq\|HepG2\|RAD21, Protein_Binding\|\|ChIP-seq\|AG04450\|CTCF, Protein_Binding\|\|ChIP-seq\|HCPEpiC\|CTCF, Protein_Binding\|02pct\|ChIP-seq\|A549\|POLR2A, Protein_Binding\|\|ChIP-seq\|HeLa-S3\|RAD21, Protein_Binding\|\|ChIP-seq\|Gliobla\|CTCF, Protein_Binding\|\|ChIP-seq\|Caco-2\|CTCF, Protein_Binding\|\|ChIP-seq\|GM12874\|CTCF, Protein_Binding\|\|ChIP-seq\|MCF-7\|CTCF, Protein_Binding\|\|ChIP-seq\|GM12872\|CTCF, Protein_Binding\|serum_stimulated_media\|ChIP-seq\|MCF-7\|CTCF, Protein_Binding\|\|ChIP-seq\|H1-hESC\|CTCF, Protein_Binding\|\|ChIP-seq\|GM12891\|CTCF, Protein_Binding\|\|ChIP-seq\|K562\|RAD21, Protein_Binding\|\|ChIP-seq\|HA-sp\|CTCF, Protein_Binding\|\|ChIP-seq\|HMEC\|CTCF, Protein_Binding\|\|ChIP-seq\|BE2\|CTCF, Protein_Binding\|\|ChIP-seq\|HMF\|CTCF, Protein_Binding\|\|ChIP-seq\|HeLa-S3\|CTCF, Protein_Binding\|\|ChIP-seq\|GM19240\|CTCF, Protein_Binding\|\|ChIP-seq\|HRE\|CTCF, Protein_Binding\|\|ChIP-seq\|GM12878\|CTCF, Protein_Binding\|\|ChIP-seq\|HBMEC\|CTCF, Protein_Binding\|\|ChIP-seq\|SK-N-SH\|CTCF, Protein_Binding\|\|ChIP-seq\|HepG2\|CTCF, Protein_Binding\|\|ChIP-seq\|RPTEC\|CTCF, Protein_Binding\|\|ChIP-seq\|WERI-Rb-1\|CTCF, Protein_Binding\|\|ChIP-seq\|AG09319\|CTCF, Protein_Binding\|02pct\|ChIP-seq\|A549\|CTCF, Protein_Binding\|\|ChIP-seq\|GM12878\|MXI1, Protein_Binding\|\|ChIP-seq\|HFF\|CTCF, Protein_Binding\|\|ChIP-seq\|GM12865\|CTCF, Protein_Binding\|\|ChIP-seq\|HPAF\|CTCF, Protein_Binding\|\|ChIP-seq\|GM12878\|WRNIP1, Protein_Binding\|dex_100nm\|ChIP-seq\|A549\|CTCF, Protein_Binding\|\|ChIP-seq\|GM19239\|CTCF, Protein_Binding\|\|ChIP-seq\|HVMF\|CTCF, Protein_Binding\|\|ChIP-seq\|NHDF-neo\|CTCF, Protein_Binding\|02pct\|ChIP-seq\|T-47D\|CTCF, Protein_Binding\|\|ChIP-seq\|HFF-Myc\|CTCF, Protein_Binding\|\|ChIP-seq\|NH-A\|CTCF, Protein_Binding\|\|ChIP-seq\|HAc\|CTCF, Protein_Binding\|\|ChIP-seq\|GM19238\|CTCF, Protein_Binding\|\|ChIP-seq\|GM12892\|CTCF, Protein_Binding\|\|ChIP-seq\|HEEpiC\|CTCF, Protein_Binding\|\|ChIP-seq\|BJ\|CTCF, Protein_Binding\|\|ChIP-seq\|GM12878\|SMC3, Protein_Binding\|\|ChIP-seq\|A549\|RAD21, Protein_Binding\|estrogen\|ChIP-seq\|MCF-7\|CTCF, Protein_Binding\|serum_stimulated_media\|ChIP-seq\|MCF-7\|POLR2A, Protein_Binding\|\|ChIP-seq\|GM12875\|CTCF, Protein_Binding\|\|ChIP-seq\|Osteobl\|CTCF, Protein_Binding\|\|ChIP-seq\|ProgFib\|CTCF, Protein_Binding\|\|ChIP-seq\|HCFaa\|CTCF, Protein_Binding\|\|ChIP-seq\|HepG2\|SMC3, Protein_Binding\|\|ChIP-seq\|NHEK\|CTCF, Protein_Binding\|\|ChIP-seq\|HSMMtube\|CTCF, Protein_Binding\|\|ChIP-seq\|HSMM\|CTCF, Protein_Binding\|\|ChIP-seq\|NHLF\|CTCF, Protein_Binding\|\|ChIP-seq\|A549\|CTCF, Protein_Binding\|\|ChIP-seq\|HEK293\|CTCF, Protein_Binding\|\|ChIP-seq\|HUVEC\|POLR2A, Protein_Binding\|\|ChIP-seq\|NHDF-Ad\|CTCF, Protein_Binding\|\|ChIP-seq\|AG09309\|CTCF, Protein_Binding\|\|ChIP-seq\|K562\|CTCF, Protein_Binding\|vehicle\|ChIP-seq\|MCF-7\|CTCF, Protein_Binding\|\|ChIP-seq\|IMR90\|CTCF, Protein_Binding\|\|ChIP-seq\|HRPEpiC\|CTCF_ | _2b_ |  | _NA_ |  | _NA_ |
| _rs3131043_ | _A/G_ | _6_ | _30758466_ | _intron_variant_ | _ENSG00000228022_ | _HCG20_ | _5.42E-12_ | _-0.57_ | _Single_Nucleotides\|HCG27\|eQTL\|Monocytes, Chromatin_Structure\|\|DNase-seq\|Ipscwru1, Chromatin_Structure\|\|DNase-seq\|Th2, Chromatin_Structure\|\|DNase-seq\|H7es, Chromatin_Structure\|\|DNase-seq\|H1hesc, Chromatin_Structure\|\|DNase-seq\|Fibrobl, Chromatin_Structure\|\|DNase-seq\|Ips, Chromatin_Structure\|\|DNase-seq\|H9es, Chromatin_Structure\|\|DNase-seq\|Th1, Protein_Binding\|\|ChIP-seq\|H1-hESC\|TCF12, Protein_Binding\|\|ChIP-seq\|H1-hESC\|POLR2A_ | _1f_ |  | _NA_ |  | _NA_ |
| _rs2516420_ | _C/T_ | _6_ | _31449620_ |  | _NA_ | _NA_ | _NA_ | _NA_ | _No data_ | _7_ |  | _NA_ |  | _NA_ |
| _rs9271770_ | _G/A_ | _6_ | _32594248_ |  | _ENSG00000196126_ | _HLA-DRB1_ | _NA_ | _NA_ | _Chromatin_Structure\|\|DNase-seq\|Th1wb33676984, Chromatin_Structure\|\|DNase-seq\|Th1, Chromatin_Structure\|\|FAIRE\|Gm12891, Chromatin_Structure\|\|DNase-seq\|Gm06990_ | _5_ |  | _NA_ |  | _NA_ |
| _rs16878812_ | _A/G_ | _6_ | _35569562_ | _intron_variant_ | _ENSG00000198755_ | _RPL10A_ | _0.00167_ | _-0.16_ | _Chromatin_Structure\|\|DNase-seq\|Progfib, Chromatin_Structure\|\|DNase-seq\|Htr8, Chromatin_Structure\|\|DNase-seq\|Hpaec, Chromatin_Structure\|\|DNase-seq\|Hmvecdneo, Chromatin_Structure\|\|DNase-seq\|Fibropag08395, Chromatin_Structure\|\|DNase-seq\|Monocd14ro1746, Chromatin_Structure\|\|DNase-seq\|Hpde6e6e7, Chromatin_Structure\|\|DNase-seq\|Hrpe, Chromatin_Structure\|\|DNase-seq\|Huvec, Chromatin_Structure\|\|DNase-seq\|Heartoc, Chromatin_Structure\|\|DNase-seq\|Hcf, Chromatin_Structure\|\|DNase-seq\|UrotsaUt189, Chromatin_Structure\|\|DNase-seq\|Panisd, Chromatin_Structure\|\|DNase-seq\|Prec, Chromatin_Structure\|\|DNase-seq\|Fibroblgm03348, Chromatin_Structure\|Est100nm1h\|DNase-seq\|Mcf7, Chromatin_Structure\|\|DNase-seq\|Ag10803, Chromatin_Structure\|\|DNase-seq\|Rptec, Chromatin_Structure\|Hypoxlaccon\|DNase-seq\|Mcf7, Chromatin_Structure\|\|DNase-seq\|M059j, Chromatin_Structure\|\|DNase-seq\|Lncap, Chromatin_Structure\|\|DNase-seq\|Nt2d1, Chromatin_Structure\|\|DNase-seq\|Hmvecdad, Chromatin_Structure\|\|DNase-seq\|Hcfaa, Chromatin_Structure\|\|DNase-seq\|Hmveclbl, Chromatin_Structure\|\|DNase-seq\|Melano, Chromatin_Structure\|\|DNase-seq\|Wi38, Chromatin_Structure\|\|DNase-seq\|Hrgec, Chromatin_Structure\|\|DNase-seq\|Olfneurosphere, Chromatin_Structure\|\|DNase-seq\|Hffmyc, Chromatin_Structure\|\|DNase-seq\|Stellate, Chromatin_Structure\|Ctcfshrna\|DNase-seq\|Mcf7, Chromatin_Structure\|\|DNase-seq\|Hae, Chromatin_Structure\|Estctrl0h\|DNase-seq\|Mcf7, Chromatin_Structure\|\|DNase-seq\|Medullod341, Chromatin_Structure\|Serumfree\|DNase-seq\|Aosmc, Chromatin_Structure\|\|DNase-seq\|Ag04450, Chromatin_Structure\|\|DNase-seq\|H7es, Chromatin_Structure\|\|DNase-seq\|Hct116, Chromatin_Structure\|\|DNase-seq\|Hmvecdblneo, Chromatin_Structure\|\|DNase-seq\|Hsmm, Chromatin_Structure\|\|DNase-seq\|Fibropag08396, Chromatin_Structure\|\|DNase-seq\|Monocd14, Chromatin_Structure\|\|DNase-seq\|Hmf, Chromatin_Structure\|\|DNase-seq\|Hasp, Chromatin_Structure\|\|DNase-seq\|Skmc, Chromatin_Structure\|\|DNase-seq\|Panislets, Chromatin_Structure\|\|DNase-seq\|Saec, Chromatin_Structure\|Lenticon\|DNase-seq\|Fibroblgm03348, Chromatin_Structure\|\|DNase-seq\|Fibrobl, Chromatin_Structure\|\|DNase-seq\|Hmvecdblad, Chromatin_Structure\|\|DNase-seq\|Nhbera, Chromatin_Structure\|\|DNase-seq\|Hff, Chromatin_Structure\|Andro\|DNase-seq\|Lncap, Chromatin_Structure\|\|DNase-seq\|Werirb1, Chromatin_Structure\|\|DNase-seq\|Mel2183, Chromatin_Structure\|\|DNase-seq\|Hrce, Chromatin_Structure\|\|DNase-seq\|Hnpce, Chromatin_Structure\|Diffa2d\|DNase-seq\|H7es, Chromatin_Structure\|\|DNase-seq\|Mcf7, Chromatin_Structure\|\|DNase-seq\|Colo829, Chromatin_Structure\|\|DNase-seq\|Fibrop, Chromatin_Structure\|\|DNase-seq\|Nhek, Chromatin_Structure\|\|DNase-seq\|A549, Chromatin_Structure\|\|DNase-seq\|Helas3, Chromatin_Structure\|\|DNase-seq\|Hgf, Chromatin_Structure\|\|DNase-seq\|Hsmmt, Chromatin_Structure\|\|DNase-seq\|Urotsa, Chromatin_Structure\|\|DNase-seq\|Rwpe1, Chromatin_Structure\|\|DNase-seq\|Hconf, Chromatin_Structure\|\|DNase-seq\|Hmec, Chromatin_Structure\|\|DNase-seq\|Nhdfneo, Chromatin_Structure\|\|DNase-seq\|Phte, Chromatin_Structure\|\|DNase-seq\|Hmvecdlyad, Chromatin_Structure\|\|DNase-seq\|Hee, Chromatin_Structure\|Diff4d\|DNase-seq\|Lhcnm2, Chromatin_Structure\|Ohtam\|DNase-seq\|Wi38, Chromatin_Structure\|\|DNase-seq\|Imr90, Chromatin_Structure\|\|DNase-seq\|Hepg2, Chromatin_Structure\|\|DNase-seq\|Hmveclly, Chromatin_Structure\|Lentimyod\|DNase-seq\|Fibroblgm03348, Chromatin_Structure\|\|DNase-seq\|Bj, Chromatin_Structure\|Hypoxlac\|DNase-seq\|Mcf7, Chromatin_Structure\|Hypoxlac\|FAIRE\|Mcf7, Chromatin_Structure\|\|DNase-seq\|Lhcnm2, Chromatin_Structure\|\|DNase-seq\|Hsmmfshd, Chromatin_Structure\|\|DNase-seq\|Ag04449, Chromatin_Structure\|\|DNase-seq\|Fibropag20443, Chromatin_Structure\|\|DNase-seq\|Hsmmemb, Chromatin_Structure\|\|DNase-seq\|Medullo, Chromatin_Structure\|\|DNase-seq\|Hs27a, Chromatin_Structure\|Randshrna\|DNase-seq\|Mcf7, Chromatin_Structure\|\|DNase-seq\|Msc, Chromatin_Structure\|\|DNase-seq\|Nhdfad, Protein_Binding\|4ohtam_1um_12hr\|ChIP-seq\|MCF10A-Er-Src\|STAT3, Protein_Binding\|ifng30\|ChIP-seq\|HeLa-S3\|STAT1, Protein_Binding\|4ohtam_1um_36hr\|ChIP-seq\|MCF10A-Er-Src\|FOS, Protein_Binding\|02pct\|ChIP-seq\|A549\|TCF12, Protein_Binding\|01pct_12hr\|ChIP-seq\|MCF10A-Er-Src\|STAT3, Protein_Binding\|01pct\|ChIP-seq\|MCF10A-Er-Src\|STAT3, Protein_Binding\|02pct\|ChIP-seq\|A549\|FOSL2, Protein_Binding\|4ohtam_1um_36hr\|ChIP-seq\|MCF10A-Er-Src\|STAT3, Protein_Binding\|\|ChIP-seq\|HUVEC\|FOS, Protein_Binding\|\|ChIP-seq\|PFSK-1\|SIN3A, Protein_Binding\|4ohtam_1um_4hr\|ChIP-seq\|MCF10A-Er-Src\|FOS, Protein_Binding\|\|ChIP-seq\|MCF-7\|TCF7L2, Protein_Binding\|01pct_4hr\|ChIP-seq\|MCF10A-Er-Src\|STAT3, Protein_Binding\|\|ChIP-seq\|SK-N-SH\|USF1_ | _4_ |  | _NA_ |  | _NA_ |
| _rs1321310_ | _T/C_ | _6_ | _36623124_ |  | _ENSG00000164530_ | _PI16_ | _0.0314_ | _0.15_ | _Single_Nucleotides\|CDKN1A\|eQTL\|Monocytes, Motifs\|PWM\|\|Pbx1, Chromatin_Structure\|Diffa14d\|DNase-seq\|H7es, Chromatin_Structure\|\|DNase-seq\|Sknsh, Chromatin_Structure\|\|DNase-seq\|Ag10803, Chromatin_Structure\|\|DNase-seq\|Hffmyc, Chromatin_Structure\|\|DNase-seq\|H7es, Chromatin_Structure\|\|DNase-seq\|Ag09309, Chromatin_Structure\|\|DNase-seq\|Skmc, Chromatin_Structure\|\|DNase-seq\|Hff, Chromatin_Structure\|\|DNase-seq\|Nhdfneo, Chromatin_Structure\|\|DNase-seq\|Bj, Chromatin_Structure\|\|DNase-seq\|Th1, Chromatin_Structure\|\|DNase-seq\|Sknmc, Protein_Binding\|\|ChIP-seq\|SK-N-SH\|EP300, Protein_Binding\|\|ChIP-seq\|SK-N-MC\|POLR2A_ | _1d_ |  | _NA_ |  | _NA_ |
| _rs6933790_ | _T/C_ | _6_ | _41672769_ | _intron_variant_ | _ENSG00000048545_ | _GUCA1A_ | _0.0111_ | _0.26_ | _Motifs\|PWM\|\|Sox17, Motifs\|PWM\|\|Sox8, Chromatin_Structure\|\|DNase-seq\|Hpaec, Chromatin_Structure\|Est100nm1h\|DNase-seq\|Mcf7, Chromatin_Structure\|Hypoxlaccon\|DNase-seq\|Mcf7, Chromatin_Structure\|\|FAIRE\|Gm12878, Chromatin_Structure\|\|DNase-seq\|Gm12864, Chromatin_Structure\|\|DNase-seq\|Cerebrumfrontaloc, Chromatin_Structure\|\|DNase-seq\|Gm12891, Chromatin_Structure\|\|DNase-seq\|8988t, Chromatin_Structure\|\|DNase-seq\|Gm19240, Chromatin_Structure\|Ctcfshrna\|DNase-seq\|Mcf7, Chromatin_Structure\|\|DNase-seq\|Gm10248, Chromatin_Structure\|\|FAIRE\|Gm18507, Chromatin_Structure\|\|FAIRE\|Gm12892, Chromatin_Structure\|Estctrl0h\|DNase-seq\|Mcf7, Chromatin_Structure\|\|DNase-seq\|Gm20000, Chromatin_Structure\|\|DNase-seq\|Gm13976, Chromatin_Structure\|\|DNase-seq\|Fibrobl, Chromatin_Structure\|\|DNase-seq\|Nhbera, Chromatin_Structure\|\|FAIRE\|Gm19239, Chromatin_Structure\|\|DNase-seq\|K562, Chromatin_Structure\|\|DNase-seq\|Gcbcell, Chromatin_Structure\|\|DNase-seq\|Cll, Chromatin_Structure\|Saha1u72hr\|DNase-seq\|K562, Chromatin_Structure\|\|DNase-seq\|Mcf7, Chromatin_Structure\|\|DNase-seq\|Gm10266, Chromatin_Structure\|\|DNase-seq\|T47d, Chromatin_Structure\|\|DNase-seq\|Gm12865, Chromatin_Structure\|Nabut\|DNase-seq\|K562, Chromatin_Structure\|\|DNase-seq\|Gm19238, Chromatin_Structure\|Est10nm30m\|DNase-seq\|T47d, Chromatin_Structure\|\|DNase-seq\|Gm19239, Chromatin_Structure\|\|DNase-seq\|Naivebcell, Chromatin_Structure\|\|DNase-seq\|Gm13977, Chromatin_Structure\|Hypoxlac\|DNase-seq\|Mcf7, Chromatin_Structure\|\|DNase-seq\|Medullo, Chromatin_Structure\|\|FAIRE\|Gm12891, Chromatin_Structure\|\|DNase-seq\|Chorion, Chromatin_Structure\|\|DNase-seq\|Gm12892, Chromatin_Structure\|\|DNase-seq\|Gm18507, Chromatin_Structure\|\|DNase-seq\|Gm12878, Chromatin_Structure\|Randshrna\|DNase-seq\|Mcf7, Protein_Binding\|\|ChIP-seq\|GM12878\|POU2F2, Protein_Binding\|\|ChIP-seq\|Raji\|POLR2A, Protein_Binding\|\|ChIP-seq\|GM19099\|POLR2A, Protein_Binding\|\|ChIP-seq\|GM12878\|POLR2A, Protein_Binding\|\|ChIP-seq\|GM18505\|POLR2A, Protein_Binding\|\|ChIP-seq\|GM18526\|POLR2A, Protein_Binding\|\|ChIP-seq\|GM12891\|POLR2A, Protein_Binding\|\|ChIP-seq\|GM18951\|POLR2A, Protein_Binding\|\|ChIP-seq\|GM12892\|POLR2A, Protein_Binding\|\|ChIP-seq\|GM19239\|CTCF, Protein_Binding\|\|ChIP-seq\|GM15510\|POLR2A, Protein_Binding\|\|ChIP-seq\|GM19193\|POLR2A_ | _3a_ |  | _NA_ |  | _NA_ |
| _rs4711689_ | _G/A/C_ | _6_ | _41692812_ | _intron_variant_ | _NA_ | _NA_ | _NA_ | _NA_ | _Motifs\|Footprinting\|Fibrobl\|IRF-7, Motifs\|Footprinting\|Mcf7\|IRF-7, Motifs\|Footprinting\|Chorion\|IRF-7, Motifs\|Footprinting\|Gm12892\|IRF-7, Motifs\|Footprinting\|Hepatocytes\|IRF-7, Motifs\|Footprinting\|Lncap\|IRF-7, Motifs\|Footprinting\|8988t\|IRF-7, Motifs\|PWM\|\|IRF-7, Motifs\|Footprinting\|H1hesc\|IRF-7, Motifs\|Footprinting\|Gm12878\|IRF-7, Motifs\|Footprinting\|Gm12891\|IRF-7, Motifs\|Footprinting\|LncapAndro\|IRF-7, Motifs\|Footprinting\|Osteobl\|IRF-7, Chromatin_Structure\|\|DNase-seq\|Hrpe, Chromatin_Structure\|\|DNase-seq\|Hcf, Chromatin_Structure\|\|DNase-seq\|Ag09319, Chromatin_Structure\|\|DNase-seq\|Ag10803, Chromatin_Structure\|\|DNase-seq\|Lncap, Chromatin_Structure\|\|DNase-seq\|K562G2mphase, Chromatin_Structure\|\|DNase-seq\|Gm04504, Chromatin_Structure\|\|DNase-seq\|Ag09309, Chromatin_Structure\|\|DNase-seq\|Skmc, Chromatin_Structure\|\|DNase-seq\|K562, Chromatin_Structure\|\|DNase-seq\|Hrce, Chromatin_Structure\|Saha1u72hr\|DNase-seq\|K562, Chromatin_Structure\|\|DNase-seq\|Mcf7, Chromatin_Structure\|Sahactrl\|DNase-seq\|K562, Chromatin_Structure\|Nabut\|DNase-seq\|K562, Chromatin_Structure\|\|DNase-seq\|Gm04503, Chromatin_Structure\|\|DNase-seq\|Ag04449, Chromatin_Structure\|\|DNase-seq\|K562G1phase, Chromatin_Structure\|\|DNase-seq\|Sknmc, Chromatin_Structure\|\|DNase-seq\|Gm12892, Chromatin_Structure\|\|DNase-seq\|Psoasmuscleoc_ | _5_ |  | _NA_ |  | _NA_ |
| _rs62404966_ | _C/T_ | _6_ | _55712124_ | _intron_variant_ |  |  | _NA_ | _NA_ | _Motifs\|PWM\|\|T3R, Chromatin_Structure\|\|FAIRE\|UrotsaUt189, Chromatin_Structure\|Ifng4h\|FAIRE\|Helas3_ | _6_ |  | _NA_ |  | _NA_ |
| _rs6928864_ | _C/A/T_ | _6_ | _105966894_ |  |  |  | _NA_ | _NA_ | _No data_ | _7_ |  | _NA_ |  | _NA_ |
| _rs12672022_ | _T/C_ | _7_ | _45136423_ |  | _NA_ | _NA_ | _NA_ | _NA_ | _Single_Nucleotides\|KIAA0363\|eQTL\|Monocytes, Single_Nucleotides\|CCM2\|eQTL\|Monocytes_ | _6_ |  | _NA_ |  | _NA_ |
| _rs10951878_ | _C/A/T_ | _7_ | _46926695_ |  |  |  | _NA_ | _NA_ | _Motifs\|PWM\|\|FOXJ2, Protein_Binding\|\|ChIP-seq\|A549\|CEBPB, Protein_Binding\|\|ChIP-seq\|BE2\|CTCF, Protein_Binding\|\|ChIP-seq\|HeLa-S3\|CEBPB, Protein_Binding\|\|ChIP-seq\|H1-hESC\|CEBPB, Protein_Binding\|\|ChIP-seq\|IMR90\|CEBPB, Protein_Binding\|\|ChIP-seq\|HepG2\|CEBPB_ | _5_ |  | _NA_ |  | _NA_ |
| _rs3801081_ | _A/G_ | _7_ | _47511161_ | _intron_variant_ | _ENSG00000136205_ | _TNS3_ | _0.0273_ | _-0.09_ | _Motifs\|Footprinting\|8988t\|SREBP, Motifs\|Footprinting\|Helas3\|SREBP, Motifs\|Footprinting\|Gm12892\|SREBP, Motifs\|PWM\|\|SREBP, Motifs\|Footprinting\|Hepatocytes\|SREBP, Motifs\|Footprinting\|Lncap\|SREBP, Motifs\|Footprinting\|Gm12891\|SREBP, Motifs\|Footprinting\|Gm12878\|SREBP, Motifs\|Footprinting\|Gm19238\|SREBP, Motifs\|PWM\|\|SREBP1, Motifs\|Footprinting\|Mcf7Hypoxlac\|SREBP, Motifs\|Footprinting\|A549\|SREBP, Motifs\|Footprinting\|Huh7\|SREBP, Motifs\|Footprinting\|Hpde6e6e7\|SREBP, Motifs\|Footprinting\|LncapAndro\|SREBP, Motifs\|Footprinting\|Hepg2\|SREBP, Motifs\|Footprinting\|Cll\|SREBP, Chromatin_Structure\|\|DNase-seq\|Lncap, Chromatin_Structure\|\|DNase-seq\|8988t, Chromatin_Structure\|\|DNase-seq\|Olfneurosphere, Chromatin_Structure\|\|DNase-seq\|Nhdfad, Protein_Binding\|proliferation\|ChIP-seq\|Caco2\|HNF4A, Protein_Binding\|\|ChIP-seq\|PANC-1\|POLR2A_ | _2b_ |  | _NA_ |  | _NA_ |
| _rs16892766_ | _A/C_ | _8_ | _117630683_ |  |  |  | _NA_ | _NA_ | _Motifs\|Footprinting\|AosmcSerumfree\|Rhox11, Motifs\|PWM\|\|Rhox11, Motifs\|Footprinting\|Htr8\|Rhox11, Chromatin_Structure\|\|DNase-seq\|Htr8, Chromatin_Structure\|\|DNase-seq\|Fibropag08395, Chromatin_Structure\|Est10nm30m\|DNase-seq\|Ishikawa, Chromatin_Structure\|\|DNase-seq\|Prec, Chromatin_Structure\|\|DNase-seq\|Fibroblgm03348, Chromatin_Structure\|\|DNase-seq\|Rptec, Chromatin_Structure\|\|DNase-seq\|Hah, Chromatin_Structure\|\|DNase-seq\|Hvmf, Chromatin_Structure\|\|FAIRE\|Medullo, Chromatin_Structure\|Serumfree\|DNase-seq\|Aosmc, Chromatin_Structure\|\|DNase-seq\|H7es, Chromatin_Structure\|\|DNase-seq\|Hmf, Chromatin_Structure\|\|DNase-seq\|Hpaf, Chromatin_Structure\|\|DNase-seq\|Fibrobl, Chromatin_Structure\|Tam10030\|DNase-seq\|Ishikawa, Chromatin_Structure\|Diffa2d\|DNase-seq\|H7es, Chromatin_Structure\|\|DNase-seq\|Fibrop, Chromatin_Structure\|\|DNase-seq\|Nha, Chromatin_Structure\|\|DNase-seq\|Gm04503, Chromatin_Structure\|\|DNase-seq\|Nhdfneo, Chromatin_Structure\|Est10nm30m\|DNase-seq\|Ecc1, Chromatin_Structure\|\|DNase-seq\|Imr90, Chromatin_Structure\|\|DNase-seq\|Fibropag20443, Chromatin_Structure\|\|DNase-seq\|Nhlf, Chromatin_Structure\|\|DNase-seq\|Th1, Chromatin_Structure\|Dm002p1h\|DNase-seq\|Ecc1, Chromatin_Structure\|\|DNase-seq\|Msc, Protein_Binding\|dex_100nm\|ChIP-seq\|ECC-1\|NR3C1, Protein_Binding\|estradiol_10nm\|ChIP-seq\|ECC-1\|ESR1, Protein_Binding\|02pct\|ChIP-seq\|ECC-1\|FOXA1_ | _2b_ |  | _NA_ |  | _NA_ |
| _rs6983267_ | _G/T_ | _8_ | _128413305_ | _intron_variant_ | _ENSG00000212993_ | _POU5F1B_ | _0.000113_ | _0.23_ | _Chromatin_Structure\|Diffa14d\|DNase-seq\|H7es, Chromatin_Structure\|\|DNase-seq\|Htr8, Chromatin_Structure\|\|DNase-seq\|Hpde6e6e7, Chromatin_Structure\|\|DNase-seq\|K562G2mphase, Chromatin_Structure\|\|FAIRE\|Nhek, Chromatin_Structure\|Ctcfshrna\|DNase-seq\|Mcf7, Chromatin_Structure\|\|DNase-seq\|Medullod341, Chromatin_Structure\|\|DNase-seq\|H7es, Chromatin_Structure\|\|DNase-seq\|K562, Chromatin_Structure\|\|DNase-seq\|Mcf7, Chromatin_Structure\|\|DNase-seq\|Huh7, Chromatin_Structure\|\|DNase-seq\|Caco2, Chromatin_Structure\|\|DNase-seq\|Helas3, Chromatin_Structure\|Ifna4h\|DNase-seq\|Helas3, Chromatin_Structure\|\|DNase-seq\|Rwpe1, Chromatin_Structure\|\|DNase-seq\|Phte, Chromatin_Structure\|\|DNase-seq\|Hepg2, Chromatin_Structure\|\|DNase-seq\|K562G1phase, Chromatin_Structure\|\|DNase-seq\|Medullo, Chromatin_Structure\|Randshrna\|DNase-seq\|Mcf7, Chromatin_Structure\|\|FAIRE\|Hepg2, Protein_Binding\|\|ChIP-seq\|HepG2\|TCF7L2, Protein_Binding\|proliferation\|ChIP-seq\|Caco2\|HNF4A, Protein_Binding\|\|ChIP-seq\|HepG2\|FOXA1, Protein_Binding\|\|ChIP-seq\|HepG2\|HDAC2, Protein_Binding\|\|ChIP-seq\|HepG2\|EP300, Protein_Binding\|\|ChIP-seq\|HepG2\|RXRA, Protein_Binding\|\|ChIP-seq\|HeLa-S3\|TFAP2A, Protein_Binding\|differential\|ChIP-seq\|Caco2\|CDX2, Protein_Binding\|\|ChIP-seq\|HepG2\|NFIC, Protein_Binding\|\|ChIP-seq\|HepG2\|SP1, Protein_Binding\|proliferation\|ChIP-seq\|Caco2\|CDX2, Protein_Binding\|\|ChIP-seq\|HepG2\|TEAD4, Protein_Binding\|\|ChIP-seq\|HepG2\|ARID3A, Protein_Binding\|\|ChIP-seq\|HCT-116\|TCF7L2, Protein_Binding\|\|ChIP-seq\|Osteobl\|CTCF, Protein_Binding\|\|ChIP-seq\|HepG2\|HNF4G, Protein_Binding\|differential\|ChIP-seq\|Caco2\|HNF4A_ | _4_ |  | _NA_ |  | _NA_ |
| _rs4313119_ | _G/T_ | _8_ | _128571855_ |  | _NA_ | _NA_ | _NA_ | _NA_ | _Motifs\|PWM\|\|NFAT5, Motifs\|PWM\|\|MAF, Chromatin_Structure\|\|FAIRE\|Medullo, Chromatin_Structure\|\|DNase-seq\|Medullod341, Chromatin_Structure\|\|FAIRE\|Gm19239, Chromatin_Structure\|\|DNase-seq\|Medullo_ | _5_ |  | _NA_ |  | _NA_ |
| _rs1412834_ | _T/C_ | _9_ | _22110131_ | _intron_variant_ |  |  | _NA_ | _NA_ | _No data_ | _7_ |  | _NA_ |  | _NA_ |
| **_rs34405347*_** | **_T/C/G_** | **_9_** | **_101679752_** |  | **_NA_** | **_NA_** | **_NA_** | **_NA_** | **_No data_** | **_7_** |  | **_NA_** |  | **_NA_** |
| _rs10980628_ | _T/C_ | _9_ | _113671403_ | _intron_variant_ | _NA_ | _NA_ | _NA_ | _NA_ | _Motifs\|PWM\|\|IRF3, Chromatin_Structure\|\|DNase-seq\|Hpdlf, Chromatin_Structure\|\|DNase-seq\|Hcf, Chromatin_Structure\|\|DNase-seq\|M059j, Chromatin_Structure\|\|DNase-seq\|Hs5, Chromatin_Structure\|\|DNase-seq\|Hcfaa, Chromatin_Structure\|\|DNase-seq\|Wi38, Chromatin_Structure\|\|DNase-seq\|Hae, Chromatin_Structure\|\|DNase-seq\|Hsmm, Chromatin_Structure\|\|DNase-seq\|Hmf, Chromatin_Structure\|\|FAIRE\|Gliobla, Chromatin_Structure\|\|DNase-seq\|Hsmmt, Chromatin_Structure\|\|DNase-seq\|Hgf, Chromatin_Structure\|Diff4d\|DNase-seq\|Lhcnm2, Chromatin_Structure\|\|DNase-seq\|Lhcnm2, Chromatin_Structure\|\|DNase-seq\|Msc, Chromatin_Structure\|\|DNase-seq\|Rpmi7951_ | _5_ |  | _NA_ |  | _NA_ |
| _rs10795668_ | _G/A_ | _10_ | _8701219_ |  | _NA_ | _NA_ | _NA_ | _NA_ | _No data_ | _7_ |  | _NA_ |  | _NA_ |
| **_rs10994860*_** | **_C/T_** | **_10_** | **_52645424_** | **_5_prime_UTR_variant_** | **_NA_** | **_NA_** | **_NA_** | **_NA_** | **_Motifs\|PWM\|\|Atf1, Motifs\|Footprinting\|HepG2\|, Chromatin_Structure\|\|DNase-seq\|Rptec, Chromatin_Structure\|\|DNase-seq\|H7es, Chromatin_Structure\|\|DNase-seq\|Panislets, Chromatin_Structure\|\|DNase-seq\|Hrce, Chromatin_Structure\|Diffa2d\|DNase-seq\|H7es, Chromatin_Structure\|\|DNase-seq\|Huh7, Chromatin_Structure\|\|DNase-seq\|Caco2, Chromatin_Structure\|\|DNase-seq\|Huh75, Chromatin_Structure\|\|DNase-seq\|Hepg2, Chromatin_Structure\|\|FAIRE\|Hepg2, Protein_Binding\|forskolin\|ChIP-seq\|HepG2\|POLR2A, Protein_Binding\|proliferation\|ChIP-seq\|Caco2\|HNF4A, Protein_Binding\|\|ChIP-seq\|HepG2\|FOXA1, Protein_Binding\|\|ChIP-seq\|HepG2\|ELF1, Protein_Binding\|\|ChIP-seq\|HepG2\|SIN3A, Protein_Binding\|\|ChIP-seq\|HepG2\|EP300, Protein_Binding\|\|ChIP-seq\|HepG2\|RXRA, Protein_Binding\|02pct\|ChIP-seq\|A549\|TAF1, Protein_Binding\|\|ChIP-seq\|HepG2\|FOXA2, Protein_Binding\|\|ChIP-seq\|HepG2\|NFIC, Protein_Binding\|\|ChIP-seq\|HepG2\|SP1, Protein_Binding\|\|ChIP-seq\|HepG2\|HNF4A, Protein_Binding\|\|ChIP-seq\|HepG2\|ARID3A, Protein_Binding\|\|ChIP-seq\|HepG2\|CEBPB, Protein_Binding\|\|ChIP-seq\|HepG2\|HNF4G, Protein_Binding\|differential\|ChIP-seq\|Caco2\|HNF4A, Protein_Binding\|\|ChIP-seq\|HepG2\|MYBL2, Protein_Binding\|\|ChIP-seq\|HepG2\|POLR2A, Protein_Binding\|\|ChIP-seq\|HepG2\|TBP_** | **_2b_** |  | **_NA_** |  | **_NA_** |
| _rs704017_ | _A/G_ | _10_ | _80819132_ | _intron_variant_ | _ENSG00000224596_ | _ZMIZ1-AS1_ | _0.00256_ | _-0.19_ | _Chromatin_Structure\|Diffa14d\|DNase-seq\|H7es, Chromatin_Structure\|Znfb34a8\|DNase-seq\|K562, Chromatin_Structure\|Znfe103c6\|DNase-seq\|K562, Chromatin_Structure\|Znf4c50c4\|DNase-seq\|K562, Chromatin_Structure\|\|DNase-seq\|K562G2mphase, Chromatin_Structure\|\|DNase-seq\|H7es, Chromatin_Structure\|\|DNase-seq\|Panislets, Chromatin_Structure\|Znff41b2\|DNase-seq\|K562, Chromatin_Structure\|Znfa41c6\|DNase-seq\|K562, Chromatin_Structure\|\|DNase-seq\|Nhbera, Chromatin_Structure\|Diffa5d\|DNase-seq\|H7es, Chromatin_Structure\|\|DNase-seq\|K562, Chromatin_Structure\|Diffa9d\|DNase-seq\|H7es, Chromatin_Structure\|Saha1u72hr\|DNase-seq\|K562, Chromatin_Structure\|\|DNase-seq\|Hepatocytes, Chromatin_Structure\|\|DNase-seq\|Huh7, Chromatin_Structure\|\|DNase-seq\|Caco2, Chromatin_Structure\|Sahactrl\|DNase-seq\|K562, Chromatin_Structure\|Nabut\|DNase-seq\|K562, Chromatin_Structure\|Znfp5\|DNase-seq\|K562, Chromatin_Structure\|\|DNase-seq\|Huh75, Chromatin_Structure\|\|DNase-seq\|Hepg2, Chromatin_Structure\|\|DNase-seq\|K562G1phase, Chromatin_Structure\|Znfg54a11\|DNase-seq\|K562, Chromatin_Structure\|Znf2c10c5\|DNase-seq\|K562, Chromatin_Structure\|Znf4g7d3\|DNase-seq\|K562, Chromatin_Structure\|\|FAIRE\|Hepg2, Protein_Binding\|\|ChIP-seq\|HepG2\|USF1, Protein_Binding\|\|ChIP-seq\|HepG2\|ZBTB7A, Protein_Binding\|\|ChIP-seq\|HepG2\|TCF7L2, Protein_Binding\|\|ChIP-seq\|HepG2\|FOSL2, Protein_Binding\|\|ChIP-seq\|HepG2\|JUND, Protein_Binding\|\|ChIP-seq\|HepG2\|BHLHE40, Protein_Binding\|\|ChIP-seq\|HepG2\|MXI1, Protein_Binding\|proliferation\|ChIP-seq\|Caco2\|HNF4A, Protein_Binding\|\|ChIP-seq\|HepG2\|MAX, Protein_Binding\|\|ChIP-seq\|HepG2\|FOXA1, Protein_Binding\|\|ChIP-seq\|K562\|MYC, Protein_Binding\|\|ChIP-seq\|K562\|CCNT2, Protein_Binding\|\|ChIP-seq\|HepG2\|HDAC2, Protein_Binding\|\|ChIP-seq\|HepG2\|SIN3A, Protein_Binding\|\|ChIP-seq\|HepG2\|EP300, Protein_Binding\|\|ChIP-seq\|HepG2\|RAD21, Protein_Binding\|\|ChIP-seq\|HepG2\|RXRA, Protein_Binding\|\|ChIP-seq\|HepG2\|FOXA2, Protein_Binding\|\|ChIP-seq\|K562\|TEAD4, Protein_Binding\|\|ChIP-seq\|K562\|GABPB1, Protein_Binding\|\|ChIP-seq\|HepG2\|NFIC, Protein_Binding\|differential\|ChIP-seq\|Caco2\|CDX2, Protein_Binding\|\|ChIP-seq\|HepG2\|SP1, Protein_Binding\|proliferation\|ChIP-seq\|Caco2\|CDX2, Protein_Binding\|\|ChIP-seq\|HepG2\|TEAD4, Protein_Binding\|\|ChIP-seq\|HepG2\|HNF4A, Protein_Binding\|\|ChIP-seq\|K562\|MAX, Protein_Binding\|\|ChIP-seq\|K562\|RCOR1, Protein_Binding\|\|ChIP-seq\|HepG2\|CEBPD, Protein_Binding\|forskolin\|ChIP-seq\|HepG2\|HNF4A, Protein_Binding\|\|ChIP-seq\|HepG2\|ARID3A, Protein_Binding\|\|ChIP-seq\|HepG2\|CEBPB, Protein_Binding\|\|ChIP-seq\|K562\|GATA2, Protein_Binding\|\|ChIP-seq\|K562\|MEF2A, Protein_Binding\|\|ChIP-seq\|K562\|MAZ, Protein_Binding\|\|ChIP-seq\|HepG2\|HNF4G, Protein_Binding\|\|ChIP-seq\|HepG2\|SMC3, Protein_Binding\|differential\|ChIP-seq\|Caco2\|HNF4A, Protein_Binding\|\|ChIP-seq\|HepG2\|TCF12, Protein_Binding\|forskolin\|ChIP-seq\|HepG2\|CEBPB, Protein_Binding\|\|ChIP-seq\|HepG2\|MYBL2, Protein_Binding\|\|ChIP-seq\|K562\|TAL1, Protein_Binding\|ifng30\|ChIP-seq\|K562\|MYC, Protein_Binding\|\|ChIP-seq\|HepG2\|MAZ, Protein_Binding\|proliferation\|ChIP-seq\|Caco2\|GATA6_ | _4_ |  | _NA_ |  | _NA_ |
| _rs1035209_ | _C/T_ | _10_ | _101345366_ |  | _NA_ | _NA_ | _NA_ | _NA_ | _No data_ | _7_ |  | _NA_ |  | _NA_ |
| _rs4919687_ | _G/A_ | _10_ | _104595248_ | _intron_variant_ | _NA_ | _NA_ | _NA_ | _NA_ | _Motifs\|PWM\|\|Nkx3-1, Chromatin_Structure\|Ifng4h\|FAIRE\|Helas3, Chromatin_Structure\|\|DNase-seq\|Ipscwru1, Chromatin_Structure\|\|DNase-seq\|Fibrobl, Chromatin_Structure\|\|DNase-seq\|Ips, Chromatin_Structure\|\|DNase-seq\|T47d, Chromatin_Structure\|\|DNase-seq\|Helas3, Chromatin_Structure\|\|DNase-seq\|Hek293t, Chromatin_Structure\|Est10nm30m\|DNase-seq\|T47d, Chromatin_Structure\|\|DNase-seq\|Osteobl, Protein_Binding\|\|ChIP-seq\|HEK293-T-REx\|ZNF263_ | _3a_ |  | _NA_ |  | _NA_ |
| _rs12241008_ | _T/C_ | _10_ | _114280702_ | _intron_variant_ | _NA_ | _NA_ | _NA_ | _NA_ | _Chromatin_Structure\|\|FAIRE\|Gm19239, Chromatin_Structure\|\|DNase-seq\|Cerebellumoc, Protein_Binding\|\|ChIP-seq\|HeLa-S3\|MAX_ | _4_ |  | _NA_ |  | _NA_ |
| **_rs11196171*_** | **_A/G_** | **_10_** | **_114724473_** | **_intron_variant_** | **_NA_** | **_NA_** | **_NA_** | **_NA_** | **_Motifs\|PWM\|\|CDP, Motifs\|PWM\|\|MEOX2_** | **_6_** |  | **_NA_** |  | **_NA_** |
| _rs4450168_ | _A/C_ | _11_ | _10286755_ | _intron_variant_ | _ENSG00000254401_ | _RP11-179A10.1_ | _0.00159_ | _-0.31_ | _No data_ | _7_ |  | _NA_ |  | _NA_ |
| **_rs174537*_** | **_G/T_** | **_11_** | **_61552680_** | **_intron_variant_** | **_NA_** | **_NA_** | **_NA_** | **_NA_** | **_Chromatin_Structure\|\|DNase-seq\|Lncap, Chromatin_Structure\|\|DNase-seq\|Gm12891, Chromatin_Structure\|\|DNase-seq\|8988t, Chromatin_Structure\|Ifna4h\|FAIRE\|Helas3, Chromatin_Structure\|\|DNase-seq\|Huh7, Chromatin_Structure\|\|DNase-seq\|Huh75, Chromatin_Structure\|\|DNase-seq\|Osteobl, Chromatin_Structure\|\|DNase-seq\|Medullo_** | **_5_** |  | **_NA_** |  | **_NA_** |
| _rs3824999_ | _T/G_ | _11_ | _74345550_ | _intron_variant_ | _NA_ | _NA_ | _NA_ | _NA_ | _Motifs\|PWM\|\|AP-3, Motifs\|Footprinting\|Phte\|AP-3, Motifs\|Footprinting\|Helas3\|AP-3, Motifs\|PWM\|\|Sfpi1, Motifs\|Footprinting\|Helas3Ifna4h\|AP-3, Motifs\|Footprinting\|A549\|AP-3, Motifs\|Footprinting\|Hmec\|AP-3, Motifs\|Footprinting\|Mcf7Hypoxlac\|AP-3, Motifs\|Footprinting\|K562\|AP-3, Motifs\|Footprinting\|Nhek\|AP-3, Motifs\|Footprinting\|Myometr\|AP-3, Motifs\|Footprinting\|Hpde6e6e7\|AP-3, Motifs\|Footprinting\|Panisd\|AP-3, Motifs\|Footprinting\|AosmcSerumfree\|AP-3, Motifs\|Footprinting\|Gliobla\|AP-3, Motifs\|Footprinting\|Mcf7\|AP-3, Chromatin_Structure\|\|DNase-seq\|Htr8, Chromatin_Structure\|\|DNase-seq\|Hpde6e6e7, Chromatin_Structure\|Est10nm30m\|DNase-seq\|Ishikawa, Chromatin_Structure\|\|DNase-seq\|UrotsaUt189, Chromatin_Structure\|Hypoxlaccon\|DNase-seq\|Mcf7, Chromatin_Structure\|Ctcfshrna\|DNase-seq\|Mcf7, Chromatin_Structure\|\|DNase-seq\|Medullod341, Chromatin_Structure\|Serumfree\|DNase-seq\|Aosmc, Chromatin_Structure\|\|DNase-seq\|Ag09309, Chromatin_Structure\|\|DNase-seq\|K562, Chromatin_Structure\|Tam10030\|DNase-seq\|Ishikawa, Chromatin_Structure\|\|DNase-seq\|Mel2183, Chromatin_Structure\|\|FAIRE\|Helas3, Chromatin_Structure\|Saha1u72hr\|DNase-seq\|K562, Chromatin_Structure\|\|DNase-seq\|Mcf7, Chromatin_Structure\|\|DNase-seq\|Colo829, Chromatin_Structure\|\|DNase-seq\|Nhek, Chromatin_Structure\|\|DNase-seq\|T47d, Chromatin_Structure\|\|DNase-seq\|A549, Chromatin_Structure\|Sahactrl\|DNase-seq\|K562, Chromatin_Structure\|\|DNase-seq\|Helas3, Chromatin_Structure\|Ifna4h\|DNase-seq\|Helas3, Chromatin_Structure\|\|DNase-seq\|Urotsa, Chromatin_Structure\|Est10nm30m\|DNase-seq\|T47d, Chromatin_Structure\|\|DNase-seq\|Rwpe1, Chromatin_Structure\|\|DNase-seq\|Osteobl, Chromatin_Structure\|Est10nm30m\|DNase-seq\|Ecc1, Chromatin_Structure\|\|DNase-seq\|Phte, Chromatin_Structure\|Hypoxlac\|DNase-seq\|Mcf7, Chromatin_Structure\|Hypoxlac\|FAIRE\|Mcf7, Chromatin_Structure\|\|DNase-seq\|Lhcnm2, Chromatin_Structure\|\|DNase-seq\|Ag04449, Chromatin_Structure\|\|DNase-seq\|Medullo, Chromatin_Structure\|Dm002p1h\|DNase-seq\|Ecc1, Chromatin_Structure\|Randshrna\|DNase-seq\|Mcf7, Protein_Binding\|\|ChIP-seq\|K562\|JUND, Protein_Binding\|\|ChIP-seq\|HeLa-S3\|JUN, Protein_Binding\|\|ChIP-seq\|K562\|EGR1, Protein_Binding\|\|ChIP-seq\|HeLa-S3\|JUND, Protein_Binding\|02pct\|ChIP-seq\|A549\|FOSL2, Protein_Binding\|\|ChIP-seq\|K562\|FOS, Protein_Binding\|\|ChIP-seq\|K562\|MAFF_ | _2b_ |  | _NA_ |  | _NA_ |
| _rs4944940_ | _G/A_ | _11_ | _74415252_ | _intron_variant_ | _ENSG00000185162_ | _AP001324.1_ | _0.00346_ | _-0.26_ | _Chromatin_Structure\|\|DNase-seq\|Mel2183, Protein_Binding\|\|ChIP-seq\|HSPC\|IKZF1_ | _4_ |  | _NA_ |  | _NA_ |
| _rs2186607_ | _T/A_ | _11_ | _101656397_ | _intron_variant_ | _NA_ | _NA_ | _NA_ | _NA_ | _No data_ | _7_ |  | _NA_ |  | _NA_ |
| **_rs3087967*_** | **_T/C_** | **_11_** | **_111156836_** | **_3_prime_UTR_variant_** | **_ENSG00000214290_** | **_COLCA2_** | **_3.14E-28_** | **_0.69_** | **_Chromatin_Structure\|\|DNase-seq\|Hepg2, Chromatin_Structure\|\|FAIRE\|Hepg2, Protein_Binding\|\|ChIP-seq\|HepG2\|POLR2A_** | **_4_** |  | **_NA_** |  | **_NA_** |
| _rs10774214_ | _T/C_ | _12_ | _4368352_ | _intron_variant_ | _NA_ | _NA_ | _NA_ | _NA_ | _Motifs\|PWM\|\|Sox7_ | _6_ |  | _NA_ |  | _NA_ |
| **_rs3217810*_** | **_C/T_** | **_12_** | **_4388271_** | **_intron_variant_** | **_NA_** | **_NA_** | **_NA_** | **_NA_** | **_Protein_Binding\|\|ChIP-seq\|GM12878\|POLR2A, Protein_Binding\|\|ChIP-seq\|GM18951\|POLR2A, Protein_Binding\|proliferation\|ChIP-seq\|Caco2\|GATA6_** | **_5_** |  | **_NA_** |  | **_NA_** |
| **_rs3217874*_** | **_C/T_** | **_12_** | **_4400808_** | **_intron_variant_** | **_NA_** | **_NA_** | **_NA_** | **_NA_** | **_Motifs\|PWM\|\|Hdx_** | **_6_** |  | **_NA_** |  | **_NA_** |
| _rs10849432_ | _C/T_ | _12_ | _6385727_ |  | _NA_ | _NA_ | _NA_ | _NA_ | _Chromatin_Structure\|\|DNase-seq\|Lncap, Chromatin_Structure\|\|DNase-seq\|Hmvecdad, Chromatin_Structure\|\|DNase-seq\|H7es, Chromatin_Structure\|\|DNase-seq\|A549, Chromatin_Structure\|\|DNase-seq\|Hgf_ | _5_ |  | _NA_ |  | _NA_ |
| _rs10849438_ | _T/G_ | _12_ | _6412036_ |  | _ENSG00000111644_ | _ACRBP_ | _0.0147_ | _0.23_ | _Chromatin_Structure\|\|FAIRE\|UrotsaUt189, Chromatin_Structure\|\|DNase-seq\|Hcf, Chromatin_Structure\|Ifng4h\|FAIRE\|Helas3, Chromatin_Structure\|\|DNase-seq\|M059j, Chromatin_Structure\|\|DNase-seq\|Gm04504, Chromatin_Structure\|\|DNase-seq\|Hcfaa, Chromatin_Structure\|\|DNase-seq\|Wi38, Chromatin_Structure\|\|DNase-seq\|Hae, Chromatin_Structure\|\|FAIRE\|Gm18507, Chromatin_Structure\|\|DNase-seq\|Hsmm, Chromatin_Structure\|\|DNase-seq\|Hac, Chromatin_Structure\|\|DNase-seq\|Hmf, Chromatin_Structure\|\|DNase-seq\|Ag09309, Chromatin_Structure\|\|DNase-seq\|Hasp, Chromatin_Structure\|\|DNase-seq\|Hipe, Chromatin_Structure\|\|DNase-seq\|Hff, Chromatin_Structure\|\|FAIRE\|Urotsa, Chromatin_Structure\|\|DNase-seq\|Fibrop, Chromatin_Structure\|\|DNase-seq\|Nha, Chromatin_Structure\|Diff4d\|DNase-seq\|Lhcnm2, Chromatin_Structure\|Ohtam\|DNase-seq\|Wi38, Chromatin_Structure\|\|DNase-seq\|Lhcnm2, Chromatin_Structure\|\|DNase-seq\|Nhlf, Chromatin_Structure\|\|DNase-seq\|Myometr, Chromatin_Structure\|\|DNase-seq\|Hbmec, Chromatin_Structure\|\|DNase-seq\|Psoasmuscleoc, Chromatin_Structure\|\|DNase-seq\|Msc, Chromatin_Structure\|\|DNase-seq\|Nhdfad, Chromatin_Structure\|\|DNase-seq\|Aoaf, Protein_Binding\|\|ChIP-seq\|HepG2\|USF1_ | _4_ |  | _NA_ |  | _NA_ |
| _rs11064437_ | _C/T_ | _12_ | _6982162_ | _intron_variant, splice_acceptor_variant_ | _NA_ | _NA_ | _NA_ | _NA_ | _Chromatin_Structure\|\|DNase-seq\|Huvec, Chromatin_Structure\|\|DNase-seq\|Cd20ro01794, Chromatin_Structure\|\|DNase-seq\|Heartoc, Chromatin_Structure\|\|DNase-seq\|Sknsh, Chromatin_Structure\|\|DNase-seq\|Lncap, Chromatin_Structure\|\|DNase-seq\|Cerebrumfrontaloc, Chromatin_Structure\|\|DNase-seq\|Gm12891, Chromatin_Structure\|\|DNase-seq\|8988t, Chromatin_Structure\|\|DNase-seq\|Olfneurosphere, Chromatin_Structure\|\|DNase-seq\|Stellate, Chromatin_Structure\|\|DNase-seq\|Medullod341, Chromatin_Structure\|\|DNase-seq\|Gm20000, Chromatin_Structure\|\|DNase-seq\|H7es, Chromatin_Structure\|\|DNase-seq\|Hsmm, Chromatin_Structure\|\|DNase-seq\|Monocd14, Chromatin_Structure\|\|DNase-seq\|Cd4naivewb78495824, Chromatin_Structure\|\|DNase-seq\|Gm13976, Chromatin_Structure\|\|DNase-seq\|H1hesc, Chromatin_Structure\|\|DNase-seq\|Adultcd4th0, Chromatin_Structure\|Andro\|DNase-seq\|Lncap, Chromatin_Structure\|\|DNase-seq\|Adultcd4th1, Chromatin_Structure\|\|DNase-seq\|Gcbcell, Chromatin_Structure\|\|DNase-seq\|Cll, Chromatin_Structure\|\|DNase-seq\|Hepatocytes, Chromatin_Structure\|\|DNase-seq\|Cerebellumoc, Chromatin_Structure\|\|DNase-seq\|Frontalcortexoc, Chromatin_Structure\|\|DNase-seq\|A549, Chromatin_Structure\|\|DNase-seq\|Huh7, Chromatin_Structure\|\|DNase-seq\|Helas3, Chromatin_Structure\|\|DNase-seq\|Gm19238, Chromatin_Structure\|\|DNase-seq\|Huh75, Chromatin_Structure\|\|DNase-seq\|Hmec, Chromatin_Structure\|Est10nm30m\|DNase-seq\|Ecc1, Chromatin_Structure\|\|DNase-seq\|Phte, Chromatin_Structure\|\|DNase-seq\|Medullo, Chromatin_Structure\|\|DNase-seq\|Chorion, Chromatin_Structure\|\|DNase-seq\|Gm12892, Chromatin_Structure\|\|DNase-seq\|Psoasmuscleoc, Chromatin_Structure\|\|DNase-seq\|Cd20ro01778, Protein_Binding\|\|ChIP-seq\|A549\|POLR2A, Protein_Binding\|\|ChIP-seq\|HepG2\|MBD4, Protein_Binding\|\|ChIP-seq\|HeLa-S3\|CHD2, Protein_Binding\|dex_100nm\|ChIP-seq\|A549\|POLR2A, Protein_Binding\|\|ChIP-seq\|GM12878\|STAT1, Protein_Binding\|shbrg1\|ChIP-seq\|CD36\|GATA1, Protein_Binding\|\|ChIP-seq\|U87\|POLR2A, Protein_Binding\|\|ChIP-seq\|GM12878\|POLR2A, Protein_Binding\|\|ChIP-seq\|K562\|POLR2A, Protein_Binding\|\|ChIP-seq\|HepG2\|NFIC, Protein_Binding\|\|ChIP-seq\|GM12891\|POLR2A, Protein_Binding\|\|ChIP-seq\|PBDE\|POLR2A, Protein_Binding\|02pct\|ChIP-seq\|A549\|REST, Protein_Binding\|\|ChIP-seq\|H1-hESC\|CHD2, Protein_Binding\|\|ChIP-seq\|HeLa-S3\|POLR2A, Protein_Binding\|\|ChIP-seq\|HepG2\|POLR2A, Protein_Binding\|\|ChIP-seq\|HUVEC\|POLR2A, Protein_Binding\|\|ChIP-seq\|Jurkat\|ETS1_ | _4_ |  | _NA_ |  | _NA_ |
| _rs2238126_ | _A/G_ | _12_ | _12009741_ | _intron_variant_ | _NA_ | _NA_ | _NA_ | _NA_ | _No data_ | _7_ |  | _NA_ |  | _NA_ |
| _rs11610543_ | _A/G/T_ | _12_ | _43134191_ |  | _NA_ | _NA_ | _NA_ | _NA_ | _No data_ | _7_ |  | _NA_ |  | _NA_ |
| _rs11169552_ | _C/T_ | _12_ | _51155663_ |  | _NA_ | _NA_ | _NA_ | _NA_ | _Motifs\|PWM\|\|Pax-5, Chromatin_Structure\|Znfa41c6\|DNase-seq\|K562, Chromatin_Structure\|\|DNase-seq\|K562, Chromatin_Structure\|\|DNase-seq\|Huh7, Chromatin_Structure\|\|DNase-seq\|Huh75, Chromatin_Structure\|\|DNase-seq\|Be2c, Protein_Binding\|02pct\|ChIP-seq\|ECC-1\|POLR2A, Protein_Binding\|\|ChIP-seq\|K562\|POLR2A_ | _3a_ |  | _NA_ |  | _NA_ |
| **_rs4759277*_** | **_C/A_** | **_12_** | **_57533690_** | **_intron_variant_** | **_NA_** | **_NA_** | **_NA_** | **_NA_** | **_Single_Nucleotides\|STAT6\|eQTL\|Monocytes, Single_Nucleotides\|MARCH9\|eQTL\|Lymphoblastoid, Single_Nucleotides\|STAT6\|eQTL\|Lymphoblastoid, Chromatin_Structure\|Znfb34a8\|DNase-seq\|K562, Chromatin_Structure\|\|DNase-seq\|Monocd14ro1746, Chromatin_Structure\|\|FAIRE\|Htr8, Chromatin_Structure\|\|DNase-seq\|Gm12891, Chromatin_Structure\|\|DNase-seq\|Gm19240, Chromatin_Structure\|\|DNase-seq\|Hae, Chromatin_Structure\|\|DNase-seq\|Monocd14, Chromatin_Structure\|\|DNase-seq\|Hpaf, Chromatin_Structure\|\|DNase-seq\|Fibrobl, Chromatin_Structure\|\|DNase-seq\|K562, Chromatin_Structure\|\|DNase-seq\|Hnpce, Chromatin_Structure\|Saha1u72hr\|DNase-seq\|K562, Chromatin_Structure\|\|DNase-seq\|Hl60, Chromatin_Structure\|\|DNase-seq\|Huh7, Chromatin_Structure\|Sahactrl\|DNase-seq\|K562, Chromatin_Structure\|Nabut\|DNase-seq\|K562, Chromatin_Structure\|\|DNase-seq\|Huh75, Chromatin_Structure\|\|DNase-seq\|Hcm, Chromatin_Structure\|Znf2c10c5\|DNase-seq\|K562, Protein_Binding\|\|ChIP-seq\|NB4\|MAX, Protein_Binding\|\|ChIP-seq\|K562\|CEBPB_** | **_1f_** |  | **_NA_** |  | **_NA_** |
| _rs7398375_ | _C/G_ | _12_ | _57540848_ | _intron_variant_ | _ENSG00000123384.9_ | _LRP1_ | _2.24E-10_ | _-0.25_ | _Motifs\|PWM\|\|Plagl1, Chromatin_Structure\|\|DNase-seq\|Nb4, Chromatin_Structure\|\|DNase-seq\|Monocd14ro1746, Chromatin_Structure\|Znfe103c6\|DNase-seq\|K562, Chromatin_Structure\|\|DNase-seq\|Hcf, Chromatin_Structure\|Ifng4h\|FAIRE\|Helas3, Chromatin_Structure\|\|DNase-seq\|Hae, Chromatin_Structure\|\|DNase-seq\|H7es, Chromatin_Structure\|\|DNase-seq\|Monocd14, Chromatin_Structure\|\|DNase-seq\|Hpaf, Chromatin_Structure\|Znfa41c6\|DNase-seq\|K562, Chromatin_Structure\|Diffa5d\|DNase-seq\|H7es, Chromatin_Structure\|\|DNase-seq\|K562, Chromatin_Structure\|\|DNase-seq\|Adultcd4th1, Chromatin_Structure\|\|DNase-seq\|Cll, Chromatin_Structure\|Saha1u72hr\|DNase-seq\|K562, Chromatin_Structure\|\|DNase-seq\|Hl60, Chromatin_Structure\|\|DNase-seq\|Cmk, Chromatin_Structure\|\|DNase-seq\|T47d, Chromatin_Structure\|\|DNase-seq\|Huh7, Chromatin_Structure\|\|DNase-seq\|Caco2, Chromatin_Structure\|Sahactrl\|DNase-seq\|K562, Chromatin_Structure\|Nabut\|DNase-seq\|K562, Chromatin_Structure\|Znfp5\|DNase-seq\|K562, Chromatin_Structure\|Est10nm30m\|DNase-seq\|T47d, Chromatin_Structure\|\|DNase-seq\|Huh75, Chromatin_Structure\|\|DNase-seq\|Hepg2, Chromatin_Structure\|\|DNase-seq\|K562G1phase, Chromatin_Structure\|\|FAIRE\|Hepg2, Chromatin_Structure\|\|DNase-seq\|Aoaf, Protein_Binding\|\|ChIP-seq\|HepG2\|JUND, Protein_Binding\|\|ChIP-seq\|K562\|SPI1, Protein_Binding\|proliferation\|ChIP-seq\|Caco2\|HNF4A, Protein_Binding\|\|ChIP-seq\|HepG2\|FOXA1, Protein_Binding\|\|ChIP-seq\|HepG2\|HDAC2, Protein_Binding\|\|ChIP-seq\|HepG2\|EP300, Protein_Binding\|\|ChIP-seq\|HepG2\|RAD21, Protein_Binding\|\|ChIP-seq\|HepG2\|RXRA, Protein_Binding\|\|ChIP-seq\|HepG2\|FOXA2, Protein_Binding\|differential\|ChIP-seq\|Caco2\|CDX2, Protein_Binding\|\|ChIP-seq\|HepG2\|SP1, Protein_Binding\|proliferation\|ChIP-seq\|Caco2\|CDX2, Protein_Binding\|\|ChIP-seq\|HepG2\|HNF4A, Protein_Binding\|forskolin\|ChIP-seq\|HepG2\|PPARGC1A, Protein_Binding\|\|ChIP-seq\|HepG2\|CEBPD, Protein_Binding\|forskolin\|ChIP-seq\|HepG2\|HNF4A, Protein_Binding\|\|ChIP-seq\|HepG2\|HNF4G, Protein_Binding\|differential\|ChIP-seq\|Caco2\|HNF4A, Protein_Binding\|\|ChIP-seq\|HepG2\|MYBL2, Protein_Binding\|proliferation\|ChIP-seq\|Caco2\|GATA6_ | _3a_ |  | _NA_ |  | _NA_ |
| _rs3184504_ | _T/A/C/G_ | _12_ | _111884608_ | _missense_variant_ | _NA_ | _NA_ | _NA_ | _NA_ | _Motifs\|PWM\|\|Mtf1, Chromatin_Structure\|\|DNase-seq\|Wi38, Chromatin_Structure\|\|DNase-seq\|Th2, Chromatin_Structure\|\|DNase-seq\|Nhek, Chromatin_Structure\|Ohtam\|DNase-seq\|Wi38, Chromatin_Structure\|\|DNase-seq\|Th1, Chromatin_Structure\|\|FAIRE\|Huvec, Protein_Binding\|\|ChIP-seq\|U87\|POLR2A, Protein_Binding\|ifna30\|ChIP-seq\|K562\|POLR2A, Protein_Binding\|\|ChIP-seq\|GM12891\|POLR2A_ | _3a_ | _benign_ | _0_ | _deleterious_ | _0_ |
| _rs72013726_ | _ACAA/-_ | _12_ | _115890836_ |  | _NA_ | _NA_ | _NA_ | _NA_ | _Chromatin_Structure\|\|DNase-seq\|Sknsh, Chromatin_Structure\|\|DNase-seq\|Ipscwru1, Chromatin_Structure\|\|DNase-seq\|H7es, Chromatin_Structure\|\|DNase-seq\|H1hesc, Chromatin_Structure\|\|DNase-seq\|Ips, Chromatin_Structure\|\|DNase-seq\|Osteobl, Chromatin_Structure\|\|DNase-seq\|Hepg2, Protein_Binding\|\|ChIP-seq\|SH-SY5Y\|GATA2, Protein_Binding\|\|ChIP-seq\|BE2\|CTCF, Protein_Binding\|\|ChIP-seq\|SK-N-SH\|CTCF, Protein_Binding\|\|ChIP-seq\|VCaP\|AR, Protein_Binding\|\|ChIP-seq\|SH-SY5Y\|GATA3, Protein_Binding\|\|ChIP-seq\|HEK293\|TCF7L2, Protein_Binding\|\|ChIP-seq\|VCaP\|ERG_ | _4_ |  | _NA_ |  | _NA_ |
| _rs73208120_ | _T/G_ | _12_ | _117747590_ | _intron_variant_ | _NA_ | _NA_ | _NA_ | _NA_ | _Chromatin_Structure\|\|DNase-seq\|Lncap_ | _5_ |  | _NA_ |  | _NA_ |
| **_rs9537521*_** | **_G/A_** | **_13_** | **_34038180_** | **_intron_variant_** |  |  | **_NA_** | **_NA_** | **_Motifs\|PWM\|\|Oct-1, Motifs\|PWM\|\|FOXL1, Motifs\|PWM\|\|Zfp128, Motifs\|PWM\|\|POU2F1, Motifs\|PWM\|\|Irx6, Motifs\|PWM\|\|Tbp, Motifs\|PWM\|\|POU2F2, Chromatin_Structure\|\|DNase-seq\|Osteobl, Protein_Binding\|\|ChIP-seq\|HMF\|CTCF_** | **_3a_** |  | **_NA_** |  | **_NA_** |
| **_rs10161980*_** | **_C/G/T_** | **_13_** | **_34093518_** | **_intron_variant_** | **_NA_** | **_NA_** | **_NA_** | **_NA_** | **_Motifs\|Footprinting\|Myometr\|GATA-1, Motifs\|Footprinting\|A549\|GATA-1, Motifs\|Footprinting\|LncapAndro\|GATA-1, Motifs\|Footprinting\|Helas3Ifna4h\|GATA-1, Motifs\|Footprinting\|Helas3\|GATA-1, Motifs\|Footprinting\|Htr8\|GATA-1, Motifs\|Footprinting\|Huh7\|GATA-1, Motifs\|Footprinting\|Cll\|GATA-1, Motifs\|PWM\|\|GATA-1, Motifs\|Footprinting\|Gm19239\|GATA-1_** | **_6_** |  | **_NA_** |  | **_NA_** |
| _rs12427600_ | _T/C_ | _13_ | _37460648_ | _intron_variant_ | _ENSG00000120693.9_ | _SMAD9_ | _2.53E-05_ | _-0.19_ | _Chromatin_Structure\|\|DNase-seq\|Medullod341, Chromatin_Structure\|\|DNase-seq\|Medullo_ | _5_ |  | _NA_ |  | _NA_ |
| _rs45597035_ | _A/G/T_ | _13_ | _73649152_ | _intron_variant_ | _ENSG00000136122_ | _BORA_ | _0.00159_ | _0.1_ | _Chromatin_Structure\|\|FAIRE\|Gm18507, Protein_Binding\|\|ChIP-seq\|HeLa-S3\|POLR2A, Protein_Binding\|\|ChIP-seq\|NHEK\|POLR2A_ | _5_ |  | _NA_ |  | _NA_ |
| _rs78341008_ | _T/C_ | _13_ | _73791554_ |  | _NA_ | _NA_ | _NA_ | _NA_ | _Motifs\|PWM\|\|HNF4A_ | _6_ |  | _NA_ |  | _NA_ |
| _rs1330889_ | _T/A/C_ | _13_ | _78609615_ | _intron_variant_ |  |  | _NA_ | _NA_ | _No data_ | _7_ |  | _NA_ |  | _NA_ |
| _rs7993934_ | _C/T_ | _13_ | _111074915_ | _intron_variant_ |  |  | _NA_ | _NA_ | _Motifs\|PWM\|\|Bhlhb2, Motifs\|PWM\|\|CLOCK:BMAL, Motifs\|PWM\|\|Ebox, Motifs\|PWM\|\|N-Myc, Chromatin_Structure\|\|FAIRE\|Gliobla_ | _6_ |  | _NA_ |  | _NA_ |
| _rs35107139_ | _A/C/T_ | _14_ | _54419106_ | _intron_variant_ | _ENSG00000224004_ | _ATP5C1P1_ | _0.0403_ | _-0.2_ | _Motifs\|PWM\|\|EGR3, Motifs\|PWM\|\|SREBP1, Motifs\|PWM\|\|RREB1, Motifs\|PWM\|\|EGR2, Motifs\|Footprinting\|Hsmmt\|RREB1, Motifs\|PWM\|\|EGR4, Motifs\|Footprinting\|H1hesc\|RREB1, Chromatin_Structure\|\|DNase-seq\|Huvec, Chromatin_Structure\|\|DNase-seq\|Heartoc, Chromatin_Structure\|\|DNase-seq\|Lncap, Chromatin_Structure\|\|FAIRE\|Htr8, Chromatin_Structure\|\|DNase-seq\|8988t, Chromatin_Structure\|\|DNase-seq\|Hah, Chromatin_Structure\|\|DNase-seq\|Gm19240, Chromatin_Structure\|\|DNase-seq\|Gm20000, Chromatin_Structure\|\|DNase-seq\|H7es, Chromatin_Structure\|\|DNase-seq\|Hsmm, Chromatin_Structure\|\|DNase-seq\|Gm13976, Chromatin_Structure\|\|DNase-seq\|Panislets, Chromatin_Structure\|\|DNase-seq\|H1hesc, Chromatin_Structure\|Andro\|DNase-seq\|Lncap, Chromatin_Structure\|\|FAIRE\|Urotsa, Chromatin_Structure\|\|DNase-seq\|Hepatocytes, Chromatin_Structure\|\|DNase-seq\|Gm10266, Chromatin_Structure\|\|DNase-seq\|Frontalcortexoc, Chromatin_Structure\|\|DNase-seq\|T47d, Chromatin_Structure\|\|DNase-seq\|Osteobl, Chromatin_Structure\|\|DNase-seq\|Gm13977, Chromatin_Structure\|\|DNase-seq\|Hsmmfshd, Chromatin_Structure\|\|DNase-seq\|Chorion, Chromatin_Structure\|\|DNase-seq\|Myometr, Chromatin_Structure\|\|DNase-seq\|Psoasmuscleoc, Protein_Binding\|\|ChIP-seq\|HUVEC\|GATA2, Protein_Binding\|02pct\|ChIP-seq\|T-47D\|GATA3, Protein_Binding\|02pct\|ChIP-seq\|T-47D\|FOXA1_ | _2b_ |  | _NA_ |  | _NA_ |
| _rs1570405_ | _G/A_ | _14_ | _54554234_ |  |  |  | _NA_ | _NA_ | _Motifs\|PWM\|\|DEAF1, Motifs\|PWM\|\|Myf6, Chromatin_Structure\|\|DNase-seq\|Lhcnm2_ | _5_ |  | _NA_ |  | _NA_ |
| _rs17094983_ | _G/A_ | _14_ | _59189361_ |  | _NA_ | _NA_ | _NA_ | _NA_ | _Chromatin_Structure\|\|DNase-seq\|Htr8, Chromatin_Structure\|\|DNase-seq\|Hmvecdneo, Chromatin_Structure\|\|DNase-seq\|Huvec, Chromatin_Structure\|\|DNase-seq\|Hcf, Chromatin_Structure\|\|DNase-seq\|Ag10803, Chromatin_Structure\|\|DNase-seq\|Hcpe, Chromatin_Structure\|\|DNase-seq\|M059j, Chromatin_Structure\|\|FAIRE\|Htr8, Chromatin_Structure\|\|DNase-seq\|Hmvecdad, Chromatin_Structure\|\|DNase-seq\|Hcfaa, Chromatin_Structure\|\|DNase-seq\|Hmveclbl, Chromatin_Structure\|\|DNase-seq\|Hpf, Chromatin_Structure\|\|DNase-seq\|Melano, Chromatin_Structure\|\|DNase-seq\|Wi38, Chromatin_Structure\|\|DNase-seq\|Hvmf, Chromatin_Structure\|\|DNase-seq\|Hrgec, Chromatin_Structure\|\|DNase-seq\|Hffmyc, Chromatin_Structure\|\|DNase-seq\|H7es, Chromatin_Structure\|\|DNase-seq\|Hsmm, Chromatin_Structure\|\|DNase-seq\|Hmf, Chromatin_Structure\|\|DNase-seq\|Hpaf, Chromatin_Structure\|\|DNase-seq\|Skmc, Chromatin_Structure\|\|DNase-seq\|Hasp, Chromatin_Structure\|\|DNase-seq\|Saec, Chromatin_Structure\|\|DNase-seq\|Hbvp, Chromatin_Structure\|\|DNase-seq\|Hmvecdblad, Chromatin_Structure\|\|DNase-seq\|Hipe, Chromatin_Structure\|\|DNase-seq\|Hff, Chromatin_Structure\|\|DNase-seq\|Hnpce, Chromatin_Structure\|Diffa2d\|DNase-seq\|H7es, Chromatin_Structure\|\|DNase-seq\|Fibrop, Chromatin_Structure\|\|DNase-seq\|Hsmmt, Chromatin_Structure\|\|DNase-seq\|Hcm, Chromatin_Structure\|Ohtam\|DNase-seq\|Wi38, Chromatin_Structure\|Diff4d\|DNase-seq\|Lhcnm2, Chromatin_Structure\|\|DNase-seq\|Imr90, Chromatin_Structure\|\|DNase-seq\|Bj, Chromatin_Structure\|\|DNase-seq\|Lhcnm2, Chromatin_Structure\|\|FAIRE\|Huvec, Chromatin_Structure\|\|DNase-seq\|Rpmi7951_ | _5_ |  | _NA_ |  | _NA_ |
| _rs16969681_ | _C/T_ | _15_ | _32993111_ |  | _ENSG00000198838_ | _RYR3_ | _0.0215_ | _0.12_ | _Chromatin_Structure\|\|DNase-seq\|Prec, Chromatin_Structure\|Hypoxlaccon\|DNase-seq\|Mcf7, Chromatin_Structure\|\|DNase-seq\|Lncap, Chromatin_Structure\|Ctcfshrna\|DNase-seq\|Mcf7, Chromatin_Structure\|Estctrl0h\|DNase-seq\|Mcf7, Chromatin_Structure\|\|DNase-seq\|Saec, Chromatin_Structure\|Andro\|DNase-seq\|Lncap, Chromatin_Structure\|\|DNase-seq\|Mcf7, Chromatin_Structure\|\|DNase-seq\|T47d, Chromatin_Structure\|\|DNase-seq\|Helas3, Chromatin_Structure\|Est10nm30m\|DNase-seq\|T47d, Chromatin_Structure\|Hypoxlac\|DNase-seq\|Mcf7, Chromatin_Structure\|\|DNase-seq\|Nhlf, Chromatin_Structure\|Randshrna\|DNase-seq\|Mcf7, Protein_Binding\|02pct\|ChIP-seq\|T-47D\|GATA3, Protein_Binding\|\|ChIP-seq\|MCF-7\|GATA3, Protein_Binding\|differential\|ChIP-seq\|Caco2\|HNF4A, Protein_Binding\|\|ChIP-seq\|HepG2\|MYBL2, Protein_Binding\|\|ChIP-seq\|MCF-7\|TCF7L2_ | _4_ |  | _NA_ |  | _NA_ |
| _rs11632715_ | _G/A_ | _15_ | _33004247_ |  | _NA_ | _NA_ | _NA_ | _NA_ | _Motifs\|PWM\|\|STAT1_ | _6_ |  | _NA_ |  | _NA_ |
| _rs73376930_ | _A/G_ | _15_ | _33012502_ | _intron_variant_ |  |  | _NA_ | _NA_ | _Chromatin_Structure\|\|DNase-seq\|Hpdlf, Chromatin_Structure\|\|DNase-seq\|Hcpe, Chromatin_Structure\|\|DNase-seq\|Wi38, Chromatin_Structure\|\|DNase-seq\|Hvmf, Chromatin_Structure\|\|DNase-seq\|Stellate, Chromatin_Structure\|\|DNase-seq\|Hac, Chromatin_Structure\|\|DNase-seq\|Ag09309, Chromatin_Structure\|\|DNase-seq\|Hasp, Chromatin_Structure\|\|DNase-seq\|Skmc, Chromatin_Structure\|\|DNase-seq\|Hbvp, Chromatin_Structure\|\|DNase-seq\|Hipe, Chromatin_Structure\|\|DNase-seq\|Hff, Chromatin_Structure\|\|DNase-seq\|Hepatocytes, Chromatin_Structure\|\|DNase-seq\|T47d, Chromatin_Structure\|\|DNase-seq\|Huh7, Chromatin_Structure\|Est10nm30m\|DNase-seq\|T47d, Chromatin_Structure\|\|DNase-seq\|Huh75, Chromatin_Structure\|\|DNase-seq\|Gm04503, Chromatin_Structure\|Ohtam\|DNase-seq\|Wi38, Chromatin_Structure\|\|FAIRE\|Astrocy, Chromatin_Structure\|\|DNase-seq\|Medullo, Chromatin_Structure\|\|DNase-seq\|Hbmec, Chromatin_Structure\|\|DNase-seq\|Aoaf_ | _5_ |  | _NA_ |  | _NA_ |
| **_rs16959063*_** | **_G/A/C_** | **_15_** | **_33105730_** | **_intron_variant_** |  |  | **_NA_** | **_NA_** | **_No data_** | **_7_** |  | **_NA_** |  | **_NA_** |
| _rs17816465_ | _G/A_ | _15_ | _33156386_ | _intron_variant_ |  |  | _NA_ | _NA_ | _Motifs\|PWM\|\|Tcf3, Chromatin_Structure\|\|DNase-seq\|Hrpe, Chromatin_Structure\|\|DNase-seq\|Ag09319, Chromatin_Structure\|\|DNase-seq\|Hcpe, Chromatin_Structure\|\|DNase-seq\|M059j, Chromatin_Structure\|\|DNase-seq\|Lncap, Chromatin_Structure\|\|DNase-seq\|Hpf, Chromatin_Structure\|\|DNase-seq\|Hac, Chromatin_Structure\|\|DNase-seq\|Hasp, Chromatin_Structure\|Tam10030\|DNase-seq\|Ishikawa, Chromatin_Structure\|\|DNase-seq\|Hrce, Chromatin_Structure\|\|DNase-seq\|A549, Chromatin_Structure\|\|DNase-seq\|Hgf, Chromatin_Structure\|\|DNase-seq\|Hbvsmc, Chromatin_Structure\|\|DNase-seq\|Hconf, Chromatin_Structure\|\|DNase-seq\|Hcm, Chromatin_Structure\|Diff4d\|DNase-seq\|Lhcnm2, Chromatin_Structure\|\|FAIRE\|Astrocy, Chromatin_Structure\|\|DNase-seq\|Lhcnm2, Chromatin_Structure\|\|DNase-seq\|Aoaf, Protein_Binding\|4ohtam_1um_12hr\|ChIP-seq\|MCF10A-Er-Src\|STAT3, Protein_Binding\|01pct_12hr\|ChIP-seq\|MCF10A-Er-Src\|STAT3, Protein_Binding\|02pct\|ChIP-seq\|A549\|EP300, Protein_Binding\|dex_100nm\|ChIP-seq\|A549\|USF1, Protein_Binding\|01pct_4hr\|ChIP-seq\|MCF10A-Er-Src\|STAT3, Protein_Binding\|02pct\|ChIP-seq\|A549\|USF1_ | _3a_ |  | _NA_ |  | _NA_ |
| _rs4776316_ | _A/G_ | _15_ | _67007813_ | _intron_variant_ | _ENSG00000270964_ | _RP11-502I4.3_ | _0.0345_ | _0.09_ | _Motifs\|Footprinting\|Lncap\|Lmo2complex, Motifs\|PWM\|\|Lmo2complex, Motifs\|Footprinting\|H1hesc\|Lmo2complex, Motifs\|Footprinting\|Huh7\|Lmo2complex, Motifs\|Footprinting\|Hepatocytes\|Lmo2complex, Chromatin_Structure\|Diffa14d\|DNase-seq\|H7es, Chromatin_Structure\|\|DNase-seq\|Sknsh, Chromatin_Structure\|\|DNase-seq\|Hah, Chromatin_Structure\|\|DNase-seq\|Medullod341, Chromatin_Structure\|\|DNase-seq\|H7es, Chromatin_Structure\|\|DNase-seq\|H1hesc, Chromatin_Structure\|\|DNase-seq\|Fibrobl, Chromatin_Structure\|\|DNase-seq\|Werirb1, Chromatin_Structure\|\|DNase-seq\|Ips, Chromatin_Structure\|\|DNase-seq\|Hepatocytes, Chromatin_Structure\|\|DNase-seq\|Caco2, Chromatin_Structure\|\|DNase-seq\|Hepg2, Chromatin_Structure\|\|DNase-seq\|Medullo_ | _5_ |  | _NA_ |  | _NA_ |
| _rs56324967_ | _T/C_ | _15_ | _67402824_ | _intron_variant_ | _NA_ | _NA_ | _NA_ | _NA_ | _Chromatin_Structure\|\|DNase-seq\|Monocd14ro1746, Chromatin_Structure\|\|DNase-seq\|Cd20ro01794, Chromatin_Structure\|\|DNase-seq\|Gm12891, Chromatin_Structure\|\|FAIRE\|Gm18507, Chromatin_Structure\|\|FAIRE\|Gm12892, Chromatin_Structure\|\|DNase-seq\|Monocd14, Chromatin_Structure\|\|DNase-seq\|Gm13976, Chromatin_Structure\|\|FAIRE\|Gm19239, Chromatin_Structure\|\|DNase-seq\|Gcbcell, Chromatin_Structure\|\|DNase-seq\|Cll, Chromatin_Structure\|\|DNase-seq\|Hl60, Chromatin_Structure\|\|DNase-seq\|A549, Chromatin_Structure\|\|DNase-seq\|Naivebcell, Chromatin_Structure\|\|DNase-seq\|Gm13977, Chromatin_Structure\|\|DNase-seq\|Th1, Chromatin_Structure\|\|FAIRE\|Gm12891, Chromatin_Structure\|\|DNase-seq\|Gm12892, Chromatin_Structure\|\|DNase-seq\|Gm18507, Chromatin_Structure\|\|DNase-seq\|Gm12878, Protein_Binding\|\|ChIP-seq\|GM12878\|MTA3, Protein_Binding\|\|ChIP-seq\|GM12878\|SP1, Protein_Binding\|\|ChIP-seq\|GM12878\|EP300, Protein_Binding\|\|ChIP-seq\|GM12878\|POLR2A, Protein_Binding\|tnfa\|ChIP-seq\|GM19099\|NFKB1, Protein_Binding\|\|ChIP-seq\|GM12891\|POU2F2, Protein_Binding\|\|ChIP-seq\|GM12891\|POLR2A, Protein_Binding\|\|ChIP-seq\|GM12892\|POLR2A, Protein_Binding\|\|ChIP-seq\|IMR90\|POLR2A, Protein_Binding\|\|ChIP-seq\|GM12878\|BCLAF1, Protein_Binding\|\|ChIP-seq\|GM12878\|BCL3, Protein_Binding\|\|ChIP-seq\|GM12878\|CHD2, Protein_Binding\|\|ChIP-seq\|GM12878\|TCF3, Protein_Binding\|\|ChIP-seq\|GM12891\|TAF1_ | _4_ |  | _NA_ |  | _NA_ |
| _rs10152518_ | _G/A/T_ | _15_ | _68177162_ |  | _ENSG00000258484_ | _SPESP1_ | _0.0101_ | _0.23_ | _Chromatin_Structure\|\|DNase-seq\|Progfib, Chromatin_Structure\|Diffa14d\|DNase-seq\|H7es, Chromatin_Structure\|\|DNase-seq\|Ipsnihi7, Chromatin_Structure\|\|DNase-seq\|Htr8, Chromatin_Structure\|\|DNase-seq\|Hpdlf, Chromatin_Structure\|\|DNase-seq\|Fibropag08395, Chromatin_Structure\|\|DNase-seq\|Monocd14ro1746, Chromatin_Structure\|\|DNase-seq\|Hpde6e6e7, Chromatin_Structure\|\|DNase-seq\|Hrpe, Chromatin_Structure\|\|DNase-seq\|Huvec, Chromatin_Structure\|Est10nm30m\|DNase-seq\|Ishikawa, Chromatin_Structure\|\|DNase-seq\|Ipsnihi11, Chromatin_Structure\|\|DNase-seq\|Cd20ro01794, Chromatin_Structure\|\|DNase-seq\|Heartoc, Chromatin_Structure\|\|DNase-seq\|Hcf, Chromatin_Structure\|Ifng4h\|FAIRE\|Helas3, Chromatin_Structure\|\|DNase-seq\|Ag09319, Chromatin_Structure\|\|DNase-seq\|UrotsaUt189, Chromatin_Structure\|\|DNase-seq\|Panisd, Chromatin_Structure\|\|DNase-seq\|Prec, Chromatin_Structure\|\|DNase-seq\|Fibroblgm03348, Chromatin_Structure\|\|DNase-seq\|Sknsh, Chromatin_Structure\|\|DNase-seq\|Hcpe, Chromatin_Structure\|\|DNase-seq\|Ag10803, Chromatin_Structure\|Hypoxlaccon\|DNase-seq\|Mcf7, Chromatin_Structure\|\|FAIRE\|Htr8, Chromatin_Structure\|\|DNase-seq\|K562G2mphase, Chromatin_Structure\|\|DNase-seq\|Cerebrumfrontaloc, Chromatin_Structure\|\|DNase-seq\|Gm04504, Chromatin_Structure\|\|DNase-seq\|Gm12891, Chromatin_Structure\|\|DNase-seq\|8988t, Chromatin_Structure\|\|DNase-seq\|Hpf, Chromatin_Structure\|\|DNase-seq\|Gm19240, Chromatin_Structure\|\|DNase-seq\|Melano, Chromatin_Structure\|\|DNase-seq\|Wi38, Chromatin_Structure\|ra\|DNase-seq\|Sknsh, Chromatin_Structure\|\|DNase-seq\|Hvmf, Chromatin_Structure\|\|DNase-seq\|Olfneurosphere, Chromatin_Structure\|\|DNase-seq\|Stellate, Chromatin_Structure\|Ctcfshrna\|DNase-seq\|Mcf7, Chromatin_Structure\|\|DNase-seq\|Gm10248, Chromatin_Structure\|\|DNase-seq\|Hae, Chromatin_Structure\|\|DNase-seq\|Ipscwru1, Chromatin_Structure\|\|DNase-seq\|Th2, Chromatin_Structure\|\|DNase-seq\|Medullod341, Chromatin_Structure\|Serumfree\|DNase-seq\|Aosmc, Chromatin_Structure\|\|DNase-seq\|Gm20000, Chromatin_Structure\|\|DNase-seq\|H7es, Chromatin_Structure\|\|DNase-seq\|Hsmm, Chromatin_Structure\|\|DNase-seq\|Fibropag08396, Chromatin_Structure\|\|DNase-seq\|Hac, Chromatin_Structure\|\|DNase-seq\|Monocd14, Chromatin_Structure\|\|DNase-seq\|Hmf, Chromatin_Structure\|\|DNase-seq\|Ag09309, Chromatin_Structure\|\|DNase-seq\|Tregwb78495824, Chromatin_Structure\|\|DNase-seq\|Hpaf, Chromatin_Structure\|\|DNase-seq\|Skmc, Chromatin_Structure\|\|DNase-seq\|Hasp, Chromatin_Structure\|\|DNase-seq\|Panislets, Chromatin_Structure\|\|DNase-seq\|Gm13976, Chromatin_Structure\|\|DNase-seq\|Saec, Chromatin_Structure\|\|DNase-seq\|H1hesc, Chromatin_Structure\|\|DNase-seq\|Hbvp, Chromatin_Structure\|Lenticon\|DNase-seq\|Fibroblgm03348, Chromatin_Structure\|\|DNase-seq\|Adultcd4th0, Chromatin_Structure\|Ifna4h\|FAIRE\|Helas3, Chromatin_Structure\|\|DNase-seq\|Fibrobl, Chromatin_Structure\|\|DNase-seq\|Nhbera, Chromatin_Structure\|\|DNase-seq\|Th1wb33676984, Chromatin_Structure\|\|DNase-seq\|Hipe, Chromatin_Structure\|Diffa5d\|DNase-seq\|H7es, Chromatin_Structure\|\|DNase-seq\|Hff, Chromatin_Structure\|\|DNase-seq\|K562, Chromatin_Structure\|Diffa9d\|DNase-seq\|H7es, Chromatin_Structure\|Tam10030\|DNase-seq\|Ishikawa, Chromatin_Structure\|\|DNase-seq\|Adultcd4th1, Chromatin_Structure\|\|DNase-seq\|Gcbcell, Chromatin_Structure\|\|DNase-seq\|Cll, Chromatin_Structure\|\|DNase-seq\|Mel2183, Chromatin_Structure\|\|FAIRE\|Urotsa, Chromatin_Structure\|\|DNase-seq\|Hrce, Chromatin_Structure\|Saha1u72hr\|DNase-seq\|K562, Chromatin_Structure\|\|DNase-seq\|Hl60, Chromatin_Structure\|\|DNase-seq\|Ips, Chromatin_Structure\|\|DNase-seq\|Mcf7, Chromatin_Structure\|\|DNase-seq\|Colo829, Chromatin_Structure\|\|DNase-seq\|Fibrop, Chromatin_Structure\|\|DNase-seq\|Hepatocytes, Chromatin_Structure\|\|DNase-seq\|Cerebellumoc, Chromatin_Structure\|\|DNase-seq\|Gm10266, Chromatin_Structure\|\|DNase-seq\|Nhek, Chromatin_Structure\|\|DNase-seq\|Frontalcortexoc, Chromatin_Structure\|\|DNase-seq\|Gliobla, Chromatin_Structure\|\|DNase-seq\|T47d, Chromatin_Structure\|\|DNase-seq\|A549, Chromatin_Structure\|\|DNase-seq\|Huh7, Chromatin_Structure\|\|DNase-seq\|Caco2, Chromatin_Structure\|Sahactrl\|DNase-seq\|K562, Chromatin_Structure\|Nabut\|DNase-seq\|K562, Chromatin_Structure\|\|DNase-seq\|Helas3, Chromatin_Structure\|\|DNase-seq\|Hgf, Chromatin_Structure\|\|DNase-seq\|Hsmmt, Chromatin_Structure\|\|DNase-seq\|Gm19238, Chromatin_Structure\|Ifna4h\|DNase-seq\|Helas3, Chromatin_Structure\|\|DNase-seq\|Hek293t, Chromatin_Structure\|\|DNase-seq\|Nha, Chromatin_Structure\|\|DNase-seq\|Urotsa, Chromatin_Structure\|Est10nm30m\|DNase-seq\|T47d, Chromatin_Structure\|\|DNase-seq\|Gm19239, Chromatin_Structure\|\|DNase-seq\|Rwpe1, Chromatin_Structure\|\|DNase-seq\|Huh75, Chromatin_Structure\|\|DNase-seq\|Hre, Chromatin_Structure\|\|DNase-seq\|Hconf, Chromatin_Structure\|\|DNase-seq\|Hmec, Chromatin_Structure\|\|DNase-seq\|Nhdfneo, Chromatin_Structure\|\|FAIRE\|K562, Chromatin_Structure\|\|DNase-seq\|Osteobl, Chromatin_Structure\|Est10nm30m\|DNase-seq\|Ecc1, Chromatin_Structure\|\|DNase-seq\|Phte, Chromatin_Structure\|\|DNase-seq\|Naivebcell, Chromatin_Structure\|\|DNase-seq\|Hcm, Chromatin_Structure\|\|DNase-seq\|H9es, Chromatin_Structure\|\|DNase-seq\|Hee, Chromatin_Structure\|Diff4d\|DNase-seq\|Lhcnm2, Chromatin_Structure\|Ohtam\|DNase-seq\|Wi38, Chromatin_Structure\|\|DNase-seq\|Gm13977, Chromatin_Structure\|\|DNase-seq\|Imr90, Chromatin_Structure\|Lentimyod\|DNase-seq\|Fibroblgm03348, Chromatin_Structure\|\|DNase-seq\|Hepg2, Chromatin_Structure\|Hypoxlac\|DNase-seq\|Mcf7, Chromatin_Structure\|\|DNase-seq\|Bj, Chromatin_Structure\|\|DNase-seq\|Lhcnm2, Chromatin_Structure\|\|DNase-seq\|Hsmmfshd, Chromatin_Structure\|\|DNase-seq\|Ag04449, Chromatin_Structure\|\|DNase-seq\|Fibropag20443, Chromatin_Structure\|\|DNase-seq\|Hsmmemb, Chromatin_Structure\|\|DNase-seq\|Nhlf, Chromatin_Structure\|\|DNase-seq\|Th1, Chromatin_Structure\|\|DNase-seq\|Medullo, Chromatin_Structure\|\|DNase-seq\|K562G1phase, Chromatin_Structure\|\|DNase-seq\|Chorion, Chromatin_Structure\|\|DNase-seq\|Myometr, Chromatin_Structure\|Dm002p1h\|DNase-seq\|Ecc1, Chromatin_Structure\|\|DNase-seq\|Gm12892, Chromatin_Structure\|\|DNase-seq\|Gm18507, Chromatin_Structure\|\|DNase-seq\|Gm12878, Chromatin_Structure\|\|DNase-seq\|Psoasmuscleoc, Chromatin_Structure\|Randshrna\|DNase-seq\|Mcf7, Chromatin_Structure\|\|DNase-seq\|Gm06990, Chromatin_Structure\|\|DNase-seq\|Cd20ro01778, Chromatin_Structure\|\|DNase-seq\|Nhdfad, Chromatin_Structure\|\|FAIRE\|Hepg2, Chromatin_Structure\|\|DNase-seq\|Rpmi7951, Protein_Binding\|\|ChIP-seq\|SK-N-SH\|EP300, Protein_Binding\|\|ChIP-seq\|HepG2\|FOSL2, Protein_Binding\|02pct\|ChIP-seq\|A549\|BCL3, Protein_Binding\|forskolin\|ChIP-seq\|HepG2\|HSF1, Protein_Binding\|\|ChIP-seq\|Adipocytes\|HSF1, Protein_Binding\|\|ChIP-seq\|A549\|POLR2A, Protein_Binding\|\|ChIP-seq\|HEK293-T-REx\|ZNF263, Protein_Binding\|\|ChIP-seq\|HepG2\|JUND, Protein_Binding\|\|ChIP-seq\|K562\|TAF1, Protein_Binding\|\|ChIP-seq\|PANC-1\|TCF7L2, Protein_Binding\|\|ChIP-seq\|K562\|ZBTB7A, Protein_Binding\|dex_100nm\|ChIP-seq\|A549\|POLR2A, Protein_Binding\|\|ChIP-seq\|SK-N-MC\|POLR2A, Protein_Binding\|proliferation\|ChIP-seq\|Caco2\|HNF4A, Protein_Binding\|\|ChIP-seq\|H1-hESC\|TAF1, Protein_Binding\|\|ChIP-seq\|PANC-1\|SIN3A, Protein_Binding\|\|ChIP-seq\|HepG2\|MAX, Protein_Binding\|\|ChIP-seq\|HepG2\|FOXA1, Protein_Binding\|\|ChIP-seq\|K562\|KDM5B, Protein_Binding\|\|ChIP-seq\|K562\|JUND, Protein_Binding\|01pct\|ChIP-seq\|MCF10A-Er-Src\|MYC, Protein_Binding\|\|ChIP-seq\|K562\|MYC, Protein_Binding\|dex_100nm\|ChIP-seq\|ECC-1\|NR3C1, Protein_Binding\|\|ChIP-seq\|K562\|CCNT2, Protein_Binding\|dex_50nm\|ChIP-seq\|A549\|NR3C1, Protein_Binding\|\|ChIP-seq\|HeLa-S3\|TFAP2C, Protein_Binding\|02pct\|ChIP-seq\|ECC-1\|POLR2A, Protein_Binding\|\|ChIP-seq\|HepG2\|EP300, Protein_Binding\|\|ChIP-seq\|HepG2\|RAD21, Protein_Binding\|\|ChIP-seq\|A549\|MAX, Protein_Binding\|\|ChIP-seq\|HepG2\|RCOR1, Protein_Binding\|\|ChIP-seq\|SK-N-SH\|TAF1, Protein_Binding\|\|ChIP-seq\|HepG2\|RXRA, Protein_Binding\|\|ChIP-seq\|NB4\|MAX, Protein_Binding\|\|ChIP-seq\|HeLa-S3\|MAX, Protein_Binding\|\|ChIP-seq\|HeLa-S3\|MAZ, Protein_Binding\|dex_100nm\|ChIP-seq\|A549\|NR3C1, Protein_Binding\|\|ChIP-seq\|HepG2\|FOXA2, Protein_Binding\|\|ChIP-seq\|K562\|TEAD4, Protein_Binding\|\|ChIP-seq\|K562\|TBL1XR1, Protein_Binding\|\|ChIP-seq\|H1-hESC\|TEAD4, Protein_Binding\|\|ChIP-seq\|K562\|TRIM28, Protein_Binding\|\|ChIP-seq\|K562\|GABPB1, Protein_Binding\|02pct\|ChIP-seq\|A549\|POLR2A, Protein_Binding\|\|ChIP-seq\|A549\|CEBPB, Protein_Binding\|\|ChIP-seq\|HeLa-S3\|TFAP2A, Protein_Binding\|\|ChIP-seq\|K562\|ELF1, Protein_Binding\|\|ChIP-seq\|K562\|POLR2A, Protein_Binding\|ifna30\|ChIP-seq\|K562\|JUN, Protein_Binding\|\|ChIP-seq\|HepG2\|NFIC, Protein_Binding\|differential\|ChIP-seq\|Caco2\|CDX2, Protein_Binding\|\|ChIP-seq\|HepG2\|SP1, Protein_Binding\|proliferation\|ChIP-seq\|Caco2\|CDX2, Protein_Binding\|\|ChIP-seq\|HepG2\|TEAD4, Protein_Binding\|\|ChIP-seq\|HepG2\|HNF4A, Protein_Binding\|\|ChIP-seq\|HeLa-S3\|CEBPB, Protein_Binding\|\|ChIP-seq\|K562\|UBTF, Protein_Binding\|02pct\|ChIP-seq\|A549\|TCF12, Protein_Binding\|\|ChIP-seq\|NB4\|POLR2A, Protein_Binding\|forskolin\|ChIP-seq\|HepG2\|PPARGC1A, Protein_Binding\|\|ChIP-seq\|K562\|EGR1, Protein_Binding\|\|ChIP-seq\|HepG2\|YY1, Protein_Binding\|\|ChIP-seq\|K562\|MAX, Protein_Binding\|\|ChIP-seq\|K562\|RCOR1, Protein_Binding\|\|ChIP-seq\|HepG2\|CEBPD, Protein_Binding\|\|ChIP-seq\|K562\|YY1, Protein_Binding\|forskolin\|ChIP-seq\|HepG2\|HNF4A, Protein_Binding\|\|ChIP-seq\|HCT-116\|POLR2A, Protein_Binding\|\|ChIP-seq\|NB4\|MYC, Protein_Binding\|\|ChIP-seq\|K562\|BHLHE40, Protein_Binding\|02pct\|ChIP-seq\|A549\|REST, Protein_Binding\|\|ChIP-seq\|IMR90\|CEBPB, Protein_Binding\|\|ChIP-seq\|HepG2\|CEBPB, Protein_Binding\|\|ChIP-seq\|K562\|ETS1, Protein_Binding\|\|ChIP-seq\|K562\|CBX3, Protein_Binding\|\|ChIP-seq\|K562\|SP1, Protein_Binding\|4ohtam_1um_36hr\|ChIP-seq\|MCF10A-Er-Src\|STAT3, Protein_Binding\|\|ChIP-seq\|PFSK-1\|FOXP2, Protein_Binding\|02pct\|ChIP-seq\|A549\|EP300, Protein_Binding\|02pct\|ChIP-seq\|A549\|ZBTB33, Protein_Binding\|dex_100nm\|ChIP-seq\|A549\|CREB1, Protein_Binding\|\|ChIP-seq\|K562\|MAZ, Protein_Binding\|ifng30\|ChIP-seq\|K562\|POLR2A, Protein_Binding\|\|ChIP-seq\|HCT-116\|TCF7L2, Protein_Binding\|\|ChIP-seq\|GM12878\|EGR1, Protein_Binding\|\|ChIP-seq\|HepG2\|CHD2, Protein_Binding\|ifng6h\|ChIP-seq\|K562\|POLR2A, Protein_Binding\|\|ChIP-seq\|HepG2\|HNF4G, Protein_Binding\|\|ChIP-seq\|HepG2\|TAF1, Protein_Binding\|\|ChIP-seq\|GM12878\|CHD2, Protein_Binding\|\|ChIP-seq\|K562\|CHD2, Protein_Binding\|differential\|ChIP-seq\|Caco2\|HNF4A, Protein_Binding\|\|ChIP-seq\|K562\|EP300, Protein_Binding\|forskolin\|ChIP-seq\|HepG2\|CEBPB, Protein_Binding\|\|ChIP-seq\|HeLa-S3\|POLR2A, Protein_Binding\|4ohtam_1um_36hr\|ChIP-seq\|MCF10A-Er-Src\|POLR2A, Protein_Binding\|dex_100nm\|ChIP-seq\|A549\|USF1, Protein_Binding\|\|ChIP-seq\|HepG2\|MYBL2, Protein_Binding\|\|ChIP-seq\|HeLa-S3\|SMARCB1, Protein_Binding\|\|ChIP-seq\|SK-N-MC\|FOXP2, Protein_Binding\|\|ChIP-seq\|HepG2\|POLR2A, Protein_Binding\|ifng30\|ChIP-seq\|K562\|IRF1, Protein_Binding\|\|ChIP-seq\|K562\|HMGN3, Protein_Binding\|\|ChIP-seq\|HeLa-S3\|TCF7L2, Protein_Binding\|dex_5nm\|ChIP-seq\|A549\|NR3C1, Protein_Binding\|ifng30\|ChIP-seq\|K562\|MYC, Protein_Binding\|\|ChIP-seq\|HepG2\|MAZ, Protein_Binding\|\|ChIP-seq\|K562\|RBBP5, Protein_Binding\|\|ChIP-seq\|K562\|ATF3, Protein_Binding\|\|ChIP-seq\|K562\|E2F4, Protein_Binding\|02pct\|ChIP-seq\|A549\|ATF3_ | _4_ |  | _NA_ |  | _NA_ |
| **_rs7495132*_** | **_C/T_** | **_15_** | **_91172901_** | **_intron_variant_** | **_ENSG00000166819_** | **_PLIN1_** | **_0.000404_** | **_-0.37_** | **_No data_** | **_7_** |  | **_NA_** |  | **_NA_** |
| **_rs9929218*_** | **_G/A_** | **_16_** | **_68820946_** | **_intron_variant_** | **_NA_** | **_NA_** | **_NA_** | **_NA_** | **_Motifs\|PWM\|\|ERG, Chromatin_Structure\|\|DNase-seq\|Fibropag08395, Chromatin_Structure\|\|DNase-seq\|Panisd, Chromatin_Structure\|\|DNase-seq\|Fibroblgm03348, Chromatin_Structure\|\|DNase-seq\|Ag10803, Chromatin_Structure\|\|DNase-seq\|M059j, Chromatin_Structure\|\|DNase-seq\|Hah, Chromatin_Structure\|\|DNase-seq\|Hvmf, Chromatin_Structure\|\|DNase-seq\|Stellate, Chromatin_Structure\|\|DNase-seq\|Hae, Chromatin_Structure\|\|DNase-seq\|Ag09309, Chromatin_Structure\|\|DNase-seq\|Hasp, Chromatin_Structure\|Lenticon\|DNase-seq\|Fibroblgm03348, Chromatin_Structure\|\|DNase-seq\|Fibrobl, Chromatin_Structure\|\|DNase-seq\|Hipe, Chromatin_Structure\|\|DNase-seq\|Mel2183, Chromatin_Structure\|\|DNase-seq\|Colo829, Chromatin_Structure\|\|DNase-seq\|Fibrop, Chromatin_Structure\|\|DNase-seq\|Gliobla, Chromatin_Structure\|\|DNase-seq\|Huh7, Chromatin_Structure\|\|DNase-seq\|Gm04503, Chromatin_Structure\|\|DNase-seq\|Bj, Chromatin_Structure\|\|DNase-seq\|Ag04449, Chromatin_Structure\|\|DNase-seq\|Nhlf, Chromatin_Structure\|\|FAIRE\|Hepg2, Protein_Binding\|proliferation\|ChIP-seq\|Caco2\|HNF4A, Protein_Binding\|\|ChIP-seq\|HepG2\|SIN3A, Protein_Binding\|\|ChIP-seq\|HepG2\|EP300, Protein_Binding\|\|ChIP-seq\|HepG2\|RXRA, Protein_Binding\|\|ChIP-seq\|HepG2\|SP1, Protein_Binding\|\|ChIP-seq\|HepG2\|TEAD4, Protein_Binding\|differential\|ChIP-seq\|Caco2\|HNF4A, Protein_Binding\|\|ChIP-seq\|HepG2\|MYBL2_** | **_3a_** |  | **_NA_** |  | **_NA_** |
| _rs61336918_ | _A/T_ | _16_ | _80007266_ |  | _ENSG00000261390_ | _RP11-345M22.2_ | _NA_ | _NA_ | _No data_ | _7_ |  | _NA_ |  | _NA_ |
| **_rs847208*_** | **_C/A_** | **_16_** | **_86254051_** |  | **_NA_** | **_NA_** | **_NA_** | **_NA_** | **_Motifs\|Footprinting\|SkMC\|, Chromatin_Structure\|\|DNase-seq\|Hpdlf, Chromatin_Structure\|\|DNase-seq\|Panisd, Chromatin_Structure\|\|DNase-seq\|Hcpe, Chromatin_Structure\|\|DNase-seq\|Melano, Chromatin_Structure\|\|DNase-seq\|Hmf, Chromatin_Structure\|\|DNase-seq\|Hbvp, Chromatin_Structure\|\|DNase-seq\|Imr90, Chromatin_Structure\|\|DNase-seq\|Chorion, Protein_Binding\|\|ChIP-seq\|HSPC\|IKZF1, Protein_Binding\|\|ChIP-seq\|IMR90\|CEBPB, Protein_Binding\|\|ChIP-seq\|IMR90\|POLR2A_** | **_4_** |  | **_NA_** |  | **_NA_** |
| **_rs2696839*_** | **_G/A/C_** | **_16_** | **_86340448_** |  | **_NA_** | **_NA_** | **_NA_** | **_NA_** | **_Motifs\|PWM\|\|Glis2_** | **_6_** |  | **_NA_** |  | **_NA_** |
| _rs899244_ | _C/T_ | _16_ | _86700030_ | _intron_variant_ | _ENSG00000140968_ | _IRF8_ | _0.00536_ | _0.13_ | _No data_ | _7_ |  | _NA_ |  | _NA_ |
| _rs12603526_ | _T/C_ | _17_ | _800593_ | _intron_variant_ | _NA_ | _NA_ | _NA_ | _NA_ | _Motifs\|PWM\|\|FOXO3, Motifs\|PWM\|\|FOXB1, Motifs\|Footprinting\|Melano\|FOXO3, Chromatin_Structure\|\|DNase-seq\|Huvec, Chromatin_Structure\|\|DNase-seq\|Heartoc, Chromatin_Structure\|\|DNase-seq\|Melano, Chromatin_Structure\|\|DNase-seq\|Hffmyc, Chromatin_Structure\|\|DNase-seq\|Monocd14, Chromatin_Structure\|\|DNase-seq\|Hbvp, Chromatin_Structure\|\|DNase-seq\|Fibrobl, Chromatin_Structure\|\|DNase-seq\|Hff, Chromatin_Structure\|\|DNase-seq\|Nhdfneo, Chromatin_Structure\|\|DNase-seq\|Hee, Chromatin_Structure\|\|DNase-seq\|Bj, Chromatin_Structure\|\|DNase-seq\|Psoasmuscleoc, Chromatin_Structure\|\|FAIRE\|Huvec, Chromatin_Structure\|\|DNase-seq\|Nhdfad_ | _5_ |  | _NA_ |  | _NA_ |
| _rs73975588_ | _A/C_ | _17_ | _816741_ | _intron_variant_ |  |  | _NA_ | _NA_ | _Chromatin_Structure\|\|DNase-seq\|Ipsnihi7, Chromatin_Structure\|\|DNase-seq\|Fibropag08395, Chromatin_Structure\|\|DNase-seq\|Cd20ro01794, Chromatin_Structure\|Ifng4h\|FAIRE\|Helas3, Chromatin_Structure\|\|DNase-seq\|Fibroblgm03348, Chromatin_Structure\|\|DNase-seq\|Ag10803, Chromatin_Structure\|Hypoxlaccon\|DNase-seq\|Mcf7, Chromatin_Structure\|\|DNase-seq\|Lncap, Chromatin_Structure\|\|DNase-seq\|Gm12891, Chromatin_Structure\|\|DNase-seq\|Gm19240, Chromatin_Structure\|\|DNase-seq\|Hvmf, Chromatin_Structure\|\|DNase-seq\|Olfneurosphere, Chromatin_Structure\|\|DNase-seq\|Gm20000, Chromatin_Structure\|\|DNase-seq\|H7es, Chromatin_Structure\|\|DNase-seq\|Monocd14, Chromatin_Structure\|\|DNase-seq\|Gm13976, Chromatin_Structure\|\|DNase-seq\|Hbvp, Chromatin_Structure\|Lenticon\|DNase-seq\|Fibroblgm03348, Chromatin_Structure\|\|DNase-seq\|Fibrobl, Chromatin_Structure\|Diffa5d\|DNase-seq\|H7es, Chromatin_Structure\|Andro\|DNase-seq\|Lncap, Chromatin_Structure\|\|DNase-seq\|Ips, Chromatin_Structure\|\|DNase-seq\|Mcf7, Chromatin_Structure\|\|DNase-seq\|Fibrop, Chromatin_Structure\|\|DNase-seq\|Gm10266, Chromatin_Structure\|\|DNase-seq\|T47d, Chromatin_Structure\|\|DNase-seq\|Helas3, Chromatin_Structure\|Ifna4h\|DNase-seq\|Helas3, Chromatin_Structure\|\|DNase-seq\|Hek293t, Chromatin_Structure\|Est10nm30m\|DNase-seq\|T47d, Chromatin_Structure\|\|DNase-seq\|Gm13977, Chromatin_Structure\|Lentimyod\|DNase-seq\|Fibroblgm03348, Chromatin_Structure\|Hypoxlac\|DNase-seq\|Mcf7, Chromatin_Structure\|\|DNase-seq\|Fibropag20443, Chromatin_Structure\|\|DNase-seq\|Medullo, Chromatin_Structure\|\|DNase-seq\|Gm12892_ | _5_ |  | _NA_ |  | _NA_ |
| _rs1078643_ | _G/A/C_ | _17_ | _10707241_ | _intron_variant_ | _ENSG00000065325_ | _GLP2R_ | _0.0446_ | _0.14_ | _Chromatin_Structure\|\|DNase-seq\|Gm12891_ | _5_ |  | _NA_ |  | _NA_ |
| _rs17836917_ | _G/A_ | _17_ | _32047282_ | _intron_variant_ | _NA_ | _NA_ | _NA_ | _NA_ | _Protein_Binding\|\|ChIP-seq\|HSPC\|IKZF1_ | _5_ |  | _NA_ |  | _NA_ |
| _rs983318_ | _G/A_ | _17_ | _70413253_ | _intron_variant_ | _NA_ | _NA_ | _NA_ | _NA_ | _Motifs\|PWM\|\|Gata3, Chromatin_Structure\|\|FAIRE\|UrotsaUt189, Chromatin_Structure\|\|DNase-seq\|Werirb1, Chromatin_Structure\|\|FAIRE\|Urotsa, Chromatin_Structure\|\|DNase-seq\|T47d, Chromatin_Structure\|\|DNase-seq\|Osteobl, Protein_Binding\|\|ChIP-seq\|U2OS\|SETDB1_ | _3a_ |  | _NA_ |  | _NA_ |
| _rs75954926_ | _A/G_ | _17_ | _81061048_ |  | _NA_ | _NA_ | _NA_ | _NA_ | _Related_Data\|methyl-Seq\|GM12878\|Differentially methylated region, Chromatin_Structure\|\|DNase-seq\|Monocd14ro1746, Chromatin_Structure\|Est10nm30m\|DNase-seq\|Ishikawa, Chromatin_Structure\|\|DNase-seq\|Ipsnihi11, Chromatin_Structure\|\|DNase-seq\|Cd20ro01794, Chromatin_Structure\|\|DNase-seq\|Heartoc, Chromatin_Structure\|Ifng4h\|FAIRE\|Helas3, Chromatin_Structure\|\|DNase-seq\|UrotsaUt189, Chromatin_Structure\|\|DNase-seq\|Sknsh, Chromatin_Structure\|Hypoxlaccon\|DNase-seq\|Mcf7, Chromatin_Structure\|\|DNase-seq\|Lncap, Chromatin_Structure\|\|DNase-seq\|K562G2mphase, Chromatin_Structure\|\|DNase-seq\|Cerebrumfrontaloc, Chromatin_Structure\|\|DNase-seq\|Gm12891, Chromatin_Structure\|\|DNase-seq\|8988t, Chromatin_Structure\|\|DNase-seq\|Gm10248, Chromatin_Structure\|\|DNase-seq\|Ipscwru1, Chromatin_Structure\|\|DNase-seq\|Medullod341, Chromatin_Structure\|\|DNase-seq\|Gm20000, Chromatin_Structure\|\|DNase-seq\|H7es, Chromatin_Structure\|\|DNase-seq\|Fibropag08396, Chromatin_Structure\|\|DNase-seq\|Monocd14, Chromatin_Structure\|\|DNase-seq\|Panislets, Chromatin_Structure\|\|DNase-seq\|Gm13976, Chromatin_Structure\|\|DNase-seq\|H1hesc, Chromatin_Structure\|\|DNase-seq\|Adultcd4th0, Chromatin_Structure\|Ifna4h\|FAIRE\|Helas3, Chromatin_Structure\|\|DNase-seq\|K562, Chromatin_Structure\|Tam10030\|DNase-seq\|Ishikawa, Chromatin_Structure\|Andro\|DNase-seq\|Lncap, Chromatin_Structure\|\|DNase-seq\|Adultcd4th1, Chromatin_Structure\|Saha1u72hr\|DNase-seq\|K562, Chromatin_Structure\|\|DNase-seq\|Ips, Chromatin_Structure\|\|DNase-seq\|Mcf7, Chromatin_Structure\|\|DNase-seq\|Fibrop, Chromatin_Structure\|\|DNase-seq\|Hepatocytes, Chromatin_Structure\|\|DNase-seq\|Gm10266, Chromatin_Structure\|\|DNase-seq\|Nhek, Chromatin_Structure\|\|DNase-seq\|Frontalcortexoc, Chromatin_Structure\|\|DNase-seq\|T47d, Chromatin_Structure\|\|DNase-seq\|A549, Chromatin_Structure\|\|DNase-seq\|Huh7, Chromatin_Structure\|Sahactrl\|DNase-seq\|K562, Chromatin_Structure\|Nabut\|DNase-seq\|K562, Chromatin_Structure\|\|DNase-seq\|Helas3, Chromatin_Structure\|\|DNase-seq\|Gm19238, Chromatin_Structure\|Ifna4h\|DNase-seq\|Helas3, Chromatin_Structure\|\|DNase-seq\|Hek293t, Chromatin_Structure\|\|DNase-seq\|Urotsa, Chromatin_Structure\|Est10nm30m\|DNase-seq\|T47d, Chromatin_Structure\|\|DNase-seq\|Gm19239, Chromatin_Structure\|\|DNase-seq\|Huh75, Chromatin_Structure\|\|DNase-seq\|Osteobl, Chromatin_Structure\|Est10nm30m\|DNase-seq\|Ecc1, Chromatin_Structure\|\|DNase-seq\|Naivebcell, Chromatin_Structure\|\|DNase-seq\|Gm13977, Chromatin_Structure\|\|DNase-seq\|Imr90, Chromatin_Structure\|\|DNase-seq\|Hepg2, Chromatin_Structure\|Hypoxlac\|DNase-seq\|Mcf7, Chromatin_Structure\|\|DNase-seq\|K562G1phase, Chromatin_Structure\|\|DNase-seq\|Medullo, Chromatin_Structure\|\|DNase-seq\|Chorion, Chromatin_Structure\|\|DNase-seq\|Myometr, Chromatin_Structure\|Dm002p1h\|DNase-seq\|Ecc1, Chromatin_Structure\|\|DNase-seq\|Gm12892, Chromatin_Structure\|\|DNase-seq\|Psoasmuscleoc, Chromatin_Structure\|Randshrna\|DNase-seq\|Mcf7, Protein_Binding\|\|ChIP-seq\|K562\|MAZ_ | _4_ |  | _NA_ |  | _NA_ |
| _rs4939827_ | _T/A/C_ | _18_ | _46453463_ | _intron_variant_ | _NA_ | _NA_ | _NA_ | _NA_ | _Motifs\|PWM\|\|Elf-1, Motifs\|PWM\|\|SPIB, Motifs\|PWM\|\|PU.1, Motifs\|PWM\|\|SPIC, Motifs\|PWM\|\|SPI1, Motifs\|PWM\|\|Spic, Motifs\|PWM\|\|Elf3, Motifs\|Footprinting\|Chorion\|Elf-1, Motifs\|Footprinting\|Chorion\|PU.1, Chromatin_Structure\|\|DNase-seq\|Nb4, Chromatin_Structure\|\|DNase-seq\|Monocd14ro1746, Chromatin_Structure\|\|DNase-seq\|Cd34mobilized, Chromatin_Structure\|\|FAIRE\|Gm12892, Chromatin_Structure\|\|DNase-seq\|H7es, Chromatin_Structure\|\|DNase-seq\|Monocd14, Chromatin_Structure\|\|DNase-seq\|K562, Chromatin_Structure\|\|DNase-seq\|Adultcd4th1, Chromatin_Structure\|\|DNase-seq\|Gcbcell, Chromatin_Structure\|\|DNase-seq\|Hepatocytes, Chromatin_Structure\|Znfp5\|DNase-seq\|K562, Chromatin_Structure\|\|DNase-seq\|Naivebcell, Chromatin_Structure\|\|DNase-seq\|Medullo_ | _5_ |  | _NA_ |  | _NA_ |
| _rs285245_ | _C/T_ | _19_ | _16420817_ | _intron_variant_ |  |  | _NA_ | _NA_ | _Motifs\|PWM\|\|ZSCAN4, Motifs\|PWM\|\|Gm397_ | _6_ |  | _NA_ |  | _NA_ |
| _rs10411210_ | _C/T_ | _19_ | _33532300_ | _intron_variant_ | _NA_ | _NA_ | _NA_ | _NA_ | _Motifs\|Footprinting\|Cll\|STAT3, Motifs\|Footprinting\|Htr8\|STAT3, Motifs\|Footprinting\|Fibrop\|FOXO1, Motifs\|Footprinting\|A549\|STAT3, Motifs\|PWM\|\|Tcf3, Motifs\|Footprinting\|Hepatocytes\|STAT3, Motifs\|Footprinting\|Hsmm\|FOXO1, Motifs\|Footprinting\|Hepg2\|STAT3, Motifs\|Footprinting\|Melano\|FOXO1, Motifs\|PWM\|\|STAT3, Motifs\|Footprinting\|Phte\|STAT3, Motifs\|Footprinting\|Hepg2\|FOXO1, Motifs\|Footprinting\|Huh75\|STAT3, Motifs\|Footprinting\|Hsmmt\|STAT3, Motifs\|Footprinting\|Gm12891\|FOXO1, Motifs\|Footprinting\|Phte\|FOXO1, Motifs\|Footprinting\|Hpde6e6e7\|STAT3, Motifs\|Footprinting\|A549\|, Motifs\|Footprinting\|Helas3\|STAT3, Motifs\|Footprinting\|SkMC\|, Motifs\|Footprinting\|H1hesc\|STAT3, Motifs\|Footprinting\|Melano\|STAT3, Motifs\|Footprinting\|Hsmm\|STAT3, Motifs\|Footprinting\|8988t\|STAT3, Motifs\|Footprinting\|Osteobl\|FOXO1, Motifs\|Footprinting\|Huh7\|STAT3, Motifs\|Footprinting\|8988t\|FOXO1, Motifs\|Footprinting\|Mcf7\|STAT3, Motifs\|Footprinting\|Gm19239\|STAT3, Motifs\|Footprinting\|Osteobl\|STAT3, Motifs\|Footprinting\|Lncap\|FOXO1, Motifs\|Footprinting\|Myometr\|FOXO1, Motifs\|Footprinting\|Helas3Ifna4h\|STAT3, Motifs\|Footprinting\|H1hesc\|FOXO1, Motifs\|Footprinting\|Huh75\|FOXO1, Motifs\|Footprinting\|Panislets\|STAT3, Motifs\|Footprinting\|Helas3\|FOXO1, Motifs\|Footprinting\|Panisd\|FOXO1, Motifs\|PWM\|\|FOXO1, Chromatin_Structure\|\|DNase-seq\|Progfib, Chromatin_Structure\|Diffa14d\|DNase-seq\|H7es, Chromatin_Structure\|\|DNase-seq\|Htr8, Chromatin_Structure\|\|DNase-seq\|Fibropag08395, Chromatin_Structure\|\|DNase-seq\|Hpde6e6e7, Chromatin_Structure\|\|DNase-seq\|Hrpe, Chromatin_Structure\|\|DNase-seq\|Hcf, Chromatin_Structure\|Ifng4h\|FAIRE\|Helas3, Chromatin_Structure\|\|DNase-seq\|Ag09319, Chromatin_Structure\|\|DNase-seq\|UrotsaUt189, Chromatin_Structure\|\|DNase-seq\|Prec, Chromatin_Structure\|Est100nm1h\|DNase-seq\|Mcf7, Chromatin_Structure\|\|DNase-seq\|Hcpe, Chromatin_Structure\|\|DNase-seq\|Ag10803, Chromatin_Structure\|Hypoxlaccon\|DNase-seq\|Mcf7, Chromatin_Structure\|\|DNase-seq\|M059j, Chromatin_Structure\|\|DNase-seq\|Lncap, Chromatin_Structure\|\|DNase-seq\|Nt2d1, Chromatin_Structure\|\|DNase-seq\|Cerebrumfrontaloc, Chromatin_Structure\|\|DNase-seq\|Gm04504, Chromatin_Structure\|\|DNase-seq\|Hcfaa, Chromatin_Structure\|\|DNase-seq\|8988t, Chromatin_Structure\|\|DNase-seq\|Hah, Chromatin_Structure\|\|DNase-seq\|Melano, Chromatin_Structure\|\|DNase-seq\|Hvmf, Chromatin_Structure\|\|DNase-seq\|Hrgec, Chromatin_Structure\|\|DNase-seq\|Hffmyc, Chromatin_Structure\|Ctcfshrna\|DNase-seq\|Mcf7, Chromatin_Structure\|\|DNase-seq\|Hmvecdlyneo, Chromatin_Structure\|\|DNase-seq\|Hae, Chromatin_Structure\|Estctrl0h\|DNase-seq\|Mcf7, Chromatin_Structure\|\|DNase-seq\|Hct116, Chromatin_Structure\|\|DNase-seq\|Hmvecdblneo, Chromatin_Structure\|\|DNase-seq\|H7es, Chromatin_Structure\|\|DNase-seq\|Hsmm, Chromatin_Structure\|\|DNase-seq\|Hmf, Chromatin_Structure\|\|DNase-seq\|Hpaf, Chromatin_Structure\|\|DNase-seq\|Skmc, Chromatin_Structure\|\|DNase-seq\|Hasp, Chromatin_Structure\|\|DNase-seq\|Panislets, Chromatin_Structure\|\|DNase-seq\|Saec, Chromatin_Structure\|Ifna4h\|FAIRE\|Helas3, Chromatin_Structure\|\|DNase-seq\|Hmvecdblad, Chromatin_Structure\|\|DNase-seq\|Nhbera, Chromatin_Structure\|Diffa5d\|DNase-seq\|H7es, Chromatin_Structure\|\|DNase-seq\|Hff, Chromatin_Structure\|\|DNase-seq\|Mel2183, Chromatin_Structure\|\|FAIRE\|Helas3, Chromatin_Structure\|\|DNase-seq\|Hrce, Chromatin_Structure\|Diffa2d\|DNase-seq\|H7es, Chromatin_Structure\|\|DNase-seq\|Mcf7, Chromatin_Structure\|\|DNase-seq\|Colo829, Chromatin_Structure\|\|DNase-seq\|Fibrop, Chromatin_Structure\|\|DNase-seq\|Hepatocytes, Chromatin_Structure\|\|DNase-seq\|Nhek, Chromatin_Structure\|\|DNase-seq\|Frontalcortexoc, Chromatin_Structure\|\|DNase-seq\|T47d, Chromatin_Structure\|\|DNase-seq\|A549, Chromatin_Structure\|\|DNase-seq\|Huh7, Chromatin_Structure\|\|DNase-seq\|Caco2, Chromatin_Structure\|\|DNase-seq\|Helas3, Chromatin_Structure\|\|DNase-seq\|Hsmmt, Chromatin_Structure\|\|DNase-seq\|Hgf, Chromatin_Structure\|\|DNase-seq\|Hbvsmc, Chromatin_Structure\|Ifna4h\|DNase-seq\|Helas3, Chromatin_Structure\|\|DNase-seq\|Nha, Chromatin_Structure\|\|DNase-seq\|Urotsa, Chromatin_Structure\|\|DNase-seq\|Rwpe1, Chromatin_Structure\|\|DNase-seq\|Gm19239, Chromatin_Structure\|\|DNase-seq\|Huh75, Chromatin_Structure\|\|DNase-seq\|Hre, Chromatin_Structure\|\|DNase-seq\|Hconf, Chromatin_Structure\|\|DNase-seq\|Nhdfneo, Chromatin_Structure\|\|DNase-seq\|Phte, Chromatin_Structure\|\|DNase-seq\|Hcm, Chromatin_Structure\|\|DNase-seq\|Hmvecdlyad, Chromatin_Structure\|\|DNase-seq\|Hee, Chromatin_Structure\|\|DNase-seq\|Hepg2, Chromatin_Structure\|Hypoxlac\|DNase-seq\|Mcf7, Chromatin_Structure\|\|DNase-seq\|Bj, Chromatin_Structure\|\|DNase-seq\|Nhlf, Chromatin_Structure\|\|DNase-seq\|Th1, Chromatin_Structure\|\|DNase-seq\|Panc1, Chromatin_Structure\|\|DNase-seq\|Gm18507, Chromatin_Structure\|\|DNase-seq\|Gm12878, Chromatin_Structure\|Randshrna\|DNase-seq\|Mcf7, Chromatin_Structure\|\|DNase-seq\|Nhdfad, Chromatin_Structure\|\|FAIRE\|Hepg2, Chromatin_Structure\|\|DNase-seq\|Be2c, Chromatin_Structure\|\|FAIRE\|A549, Chromatin_Structure\|\|DNase-seq\|Aoaf, Chromatin_Structure\|\|DNase-seq\|Rpmi7951, Protein_Binding\|\|ChIP-seq\|MCF-7\|ZNF217, Protein_Binding\|02pct\|ChIP-seq\|A549\|ELF1, Protein_Binding\|\|ChIP-seq\|HepG2\|FOSL2, Protein_Binding\|02pct\|ChIP-seq\|A549\|BCL3, Protein_Binding\|\|ChIP-seq\|A549\|POLR2A, Protein_Binding\|\|ChIP-seq\|HepG2\|JUND, Protein_Binding\|02pct\|ChIP-seq\|A549\|SIX5, Protein_Binding\|\|ChIP-seq\|PANC-1\|TCF7L2, Protein_Binding\|\|ChIP-seq\|HepG2\|BHLHE40, Protein_Binding\|\|ChIP-seq\|HepG2\|MBD4, Protein_Binding\|\|ChIP-seq\|HepG2\|MXI1, Protein_Binding\|proliferation\|ChIP-seq\|Caco2\|HNF4A, Protein_Binding\|\|ChIP-seq\|PANC-1\|SIN3A, Protein_Binding\|\|ChIP-seq\|HepG2\|FOXA1, Protein_Binding\|\|ChIP-seq\|HepG2\|HDAC2, Protein_Binding\|4ohtam_1um_12hr\|ChIP-seq\|MCF10A-Er-Src\|FOS, Protein_Binding\|dex_50nm\|ChIP-seq\|A549\|NR3C1, Protein_Binding\|\|ChIP-seq\|HeLa-S3\|TFAP2C, Protein_Binding\|\|ChIP-seq\|HepG2\|SIN3A, Protein_Binding\|\|ChIP-seq\|HepG2\|EP300, Protein_Binding\|02pct\|ChIP-seq\|A549\|GABPB1, Protein_Binding\|\|ChIP-seq\|A549\|MAX, Protein_Binding\|\|ChIP-seq\|HepG2\|RCOR1, Protein_Binding\|\|ChIP-seq\|HepG2\|RXRA, Protein_Binding\|\|ChIP-seq\|HeLa-S3\|STAT3, Protein_Binding\|\|ChIP-seq\|HepG2\|FOXA2, Protein_Binding\|dex_100nm\|ChIP-seq\|A549\|NR3C1, Protein_Binding\|\|ChIP-seq\|A549\|CEBPB, Protein_Binding\|4ohtam_1um_12hr\|ChIP-seq\|MCF10A-Er-Src\|STAT3, Protein_Binding\|02pct\|ChIP-seq\|A549\|SIN3A, Protein_Binding\|ifng30\|ChIP-seq\|HeLa-S3\|STAT1, Protein_Binding\|\|ChIP-seq\|HeLa-S3\|TFAP2A, Protein_Binding\|\|ChIP-seq\|HepG2\|REST, Protein_Binding\|\|ChIP-seq\|HepG2\|NFIC, Protein_Binding\|differential\|ChIP-seq\|Caco2\|CDX2, Protein_Binding\|4ohtam_1um_36hr\|ChIP-seq\|MCF10A-Er-Src\|FOS, Protein_Binding\|\|ChIP-seq\|HepG2\|SP1, Protein_Binding\|\|ChIP-seq\|HepG2\|TEAD4, Protein_Binding\|\|ChIP-seq\|HepG2\|HNF4A, Protein_Binding\|\|ChIP-seq\|HeLa-S3\|EP300, Protein_Binding\|\|ChIP-seq\|HeLa-S3\|CEBPB, Protein_Binding\|02pct\|ChIP-seq\|A549\|TCF12, Protein_Binding\|01pct_12hr\|ChIP-seq\|MCF10A-Er-Src\|STAT3, Protein_Binding\|\|ChIP-seq\|HepG2\|YY1, Protein_Binding\|\|ChIP-seq\|PANC-1\|POLR2A, Protein_Binding\|\|ChIP-seq\|PFSK-1\|POLR2A, Protein_Binding\|forskolin\|ChIP-seq\|HepG2\|HNF4A, Protein_Binding\|\|ChIP-seq\|HCT-116\|POLR2A, Protein_Binding\|\|ChIP-seq\|HepG2\|ARID3A, Protein_Binding\|02pct\|ChIP-seq\|A549\|REST, Protein_Binding\|\|ChIP-seq\|HepG2\|CEBPB, Protein_Binding\|01pct\|ChIP-seq\|MCF10A-Er-Src\|STAT3, Protein_Binding\|02pct\|ChIP-seq\|A549\|FOSL2, Protein_Binding\|4ohtam_1um_36hr\|ChIP-seq\|MCF10A-Er-Src\|STAT3, Protein_Binding\|02pct\|ChIP-seq\|A549\|EP300, Protein_Binding\|02pct\|ChIP-seq\|A549\|ZBTB33, Protein_Binding\|dex_100nm\|ChIP-seq\|A549\|CREB1, Protein_Binding\|\|ChIP-seq\|A549\|RAD21, Protein_Binding\|4ohtam_1um_4hr\|ChIP-seq\|MCF10A-Er-Src\|FOS, Protein_Binding\|\|ChIP-seq\|HepG2\|HNF4G, Protein_Binding\|differential\|ChIP-seq\|Caco2\|HNF4A, Protein_Binding\|\|ChIP-seq\|HeLa-S3\|RFX5, Protein_Binding\|dex_100nm\|ChIP-seq\|A549\|USF1, Protein_Binding\|\|ChIP-seq\|HepG2\|MYBL2, Protein_Binding\|\|ChIP-seq\|MCF-7\|TCF7L2, Protein_Binding\|\|ChIP-seq\|HeLa-S3\|TCF7L2, Protein_Binding\|01pct_4hr\|ChIP-seq\|MCF10A-Er-Src\|STAT3, Protein_Binding\|\|ChIP-seq\|HepG2\|RFX5, Protein_Binding\|dex_5nm\|ChIP-seq\|A549\|NR3C1, Protein_Binding\|02pct\|ChIP-seq\|A549\|USF1, Protein_Binding\|\|ChIP-seq\|HepG2\|MAZ, Protein_Binding\|02pct\|ChIP-seq\|A549\|ETS1, Protein_Binding\|02pct\|ChIP-seq\|A549\|ATF3, Protein_Binding\|\|ChIP-seq\|HepG2\|TBP_ | _2a_ |  | _NA_ |  | _NA_ |
| _rs1800469_ | _A/G_ | _19_ | _41860296_ | _intron_variant_ | _NA_ | _NA_ | _NA_ | _NA_ | _Single_Nucleotides\|MGC4093\|eQTL\|Lymphoblastoid, Motifs\|Footprinting\|Gm12892\|COUPTF, Motifs\|Footprinting\|Huh7\|COUPTF, Motifs\|Footprinting\|Huh75\|COUPTF, Motifs\|PWM\|\|COUPTF, Motifs\|Footprinting\|Gm12891\|COUPTF, Motifs\|Footprinting\|Chorion\|COUPTF, Motifs\|Footprinting\|A549\|COUPTF, Motifs\|Footprinting\|Gm12878\|COUPTF, Motifs\|Footprinting\|H1hesc\|COUPTF, Motifs\|Footprinting\|Medullo\|COUPTF, Chromatin_Structure\|\|DNase-seq\|Progfib, Chromatin_Structure\|\|DNase-seq\|H1hesc, Chromatin_Structure\|Lenticon\|DNase-seq\|Fibroblgm03348, Chromatin_Structure\|\|DNase-seq\|Adultcd4th0, Chromatin_Structure\|\|DNase-seq\|Fibrobl, Chromatin_Structure\|\|DNase-seq\|Ipsnihi7, Chromatin_Structure\|\|DNase-seq\|Htr8, Chromatin_Structure\|\|DNase-seq\|K562, Chromatin_Structure\|\|DNase-seq\|Adultcd4th1, Chromatin_Structure\|\|DNase-seq\|Hpde6e6e7, Chromatin_Structure\|\|DNase-seq\|Gcbcell, Chromatin_Structure\|\|DNase-seq\|Cll, Chromatin_Structure\|\|DNase-seq\|Huvec, Chromatin_Structure\|Saha1u72hr\|DNase-seq\|K562, Chromatin_Structure\|\|DNase-seq\|Ipsnihi11, Chromatin_Structure\|\|DNase-seq\|Cd20ro01794, Chromatin_Structure\|\|DNase-seq\|Ips, Chromatin_Structure\|\|DNase-seq\|Mcf7, Chromatin_Structure\|\|DNase-seq\|Heartoc, Chromatin_Structure\|\|DNase-seq\|Fibrop, Chromatin_Structure\|\|DNase-seq\|UrotsaUt189, Chromatin_Structure\|\|DNase-seq\|Panisd, Chromatin_Structure\|\|DNase-seq\|Cmk, Chromatin_Structure\|\|DNase-seq\|Fibroblgm03348, Chromatin_Structure\|\|DNase-seq\|Hepatocytes, Chromatin_Structure\|\|DNase-seq\|Cerebellumoc, Chromatin_Structure\|\|DNase-seq\|Gm10266, Chromatin_Structure\|\|DNase-seq\|Nhek, Chromatin_Structure\|\|DNase-seq\|Frontalcortexoc, Chromatin_Structure\|\|DNase-seq\|Sknsh, Chromatin_Structure\|Hypoxlaccon\|DNase-seq\|Mcf7, Chromatin_Structure\|\|DNase-seq\|Gliobla, Chromatin_Structure\|\|DNase-seq\|T47d, Chromatin_Structure\|\|DNase-seq\|A549, Chromatin_Structure\|\|DNase-seq\|Huh7, Chromatin_Structure\|\|DNase-seq\|Lncap, Chromatin_Structure\|Sahactrl\|DNase-seq\|K562, Chromatin_Structure\|\|DNase-seq\|Helas3, Chromatin_Structure\|Nabut\|DNase-seq\|K562, Chromatin_Structure\|\|DNase-seq\|K562G2mphase, Chromatin_Structure\|\|DNase-seq\|Hsmmt, Chromatin_Structure\|\|DNase-seq\|Gm19238, Chromatin_Structure\|\|DNase-seq\|Cerebrumfrontaloc, Chromatin_Structure\|\|DNase-seq\|Hek293t, Chromatin_Structure\|\|DNase-seq\|Urotsa, Chromatin_Structure\|\|DNase-seq\|Gm12891, Chromatin_Structure\|\|DNase-seq\|8988t, Chromatin_Structure\|\|DNase-seq\|Gm19239, Chromatin_Structure\|\|DNase-seq\|Huh75, Chromatin_Structure\|\|DNase-seq\|Rwpe1, Chromatin_Structure\|\|DNase-seq\|Gm19240, Chromatin_Structure\|\|DNase-seq\|Hmec, Chromatin_Structure\|\|DNase-seq\|Melano, Chromatin_Structure\|\|DNase-seq\|Osteobl, Chromatin_Structure\|Est10nm30m\|DNase-seq\|Ecc1, Chromatin_Structure\|\|DNase-seq\|Naivebcell, Chromatin_Structure\|\|DNase-seq\|Olfneurosphere, Chromatin_Structure\|\|DNase-seq\|H9es, Chromatin_Structure\|Ctcfshrna\|DNase-seq\|Mcf7, Chromatin_Structure\|\|DNase-seq\|Stellate, Chromatin_Structure\|\|DNase-seq\|Gm13977, Chromatin_Structure\|\|DNase-seq\|Imr90, Chromatin_Structure\|Lentimyod\|DNase-seq\|Fibroblgm03348, Chromatin_Structure\|\|DNase-seq\|Hepg2, Chromatin_Structure\|\|DNase-seq\|Gm10248, Chromatin_Structure\|Hypoxlac\|DNase-seq\|Mcf7, Chromatin_Structure\|\|DNase-seq\|Ipscwru1, Chromatin_Structure\|\|DNase-seq\|Hsmmfshd, Chromatin_Structure\|\|DNase-seq\|Fibropag20443, Chromatin_Structure\|\|DNase-seq\|Hsmmemb, Chromatin_Structure\|\|FAIRE\|Gm12892, Chromatin_Structure\|\|DNase-seq\|Medullo, Chromatin_Structure\|\|DNase-seq\|Medullod341, Chromatin_Structure\|\|DNase-seq\|K562G1phase, Chromatin_Structure\|\|DNase-seq\|Chorion, Chromatin_Structure\|\|DNase-seq\|Myometr, Chromatin_Structure\|\|DNase-seq\|Gm20000, Chromatin_Structure\|Dm002p1h\|DNase-seq\|Ecc1, Chromatin_Structure\|\|DNase-seq\|Gm12892, Chromatin_Structure\|\|DNase-seq\|Gm18507, Chromatin_Structure\|\|DNase-seq\|H7es, Chromatin_Structure\|\|DNase-seq\|Hsmm, Chromatin_Structure\|\|DNase-seq\|Fibropag08396, Chromatin_Structure\|\|DNase-seq\|Gm12878, Chromatin_Structure\|\|DNase-seq\|Psoasmuscleoc, Chromatin_Structure\|Randshrna\|DNase-seq\|Mcf7, Chromatin_Structure\|\|DNase-seq\|Monocd14, Chromatin_Structure\|\|DNase-seq\|Cd4naivewb78495824, Chromatin_Structure\|\|DNase-seq\|Panislets, Chromatin_Structure\|\|DNase-seq\|Gm13976, Protein_Binding\|02pct\|ChIP-seq\|A549\|BCL3, Protein_Binding\|\|ChIP-seq\|H1-hESC\|MXI1, Protein_Binding\|02pct\|ChIP-seq\|A549\|YY1, Protein_Binding\|02pct\|ChIP-seq\|A549\|SIX5, Protein_Binding\|\|ChIP-seq\|GM12878\|FOXM1, Protein_Binding\|\|ChIP-seq\|GM12878\|POLR2A, Protein_Binding\|02pct\|ChIP-seq\|A549\|SIN3A, Protein_Binding\|\|ChIP-seq\|Jurkat\|CREBBP, Protein_Binding\|02pct\|ChIP-seq\|A549\|TCF12_ | _1b_ |  | _NA_ |  | _NA_ |
| _rs12979278_ | _C/T_ | _19_ | _49218602_ | _synonymous_variant_ | _ENSG00000176920_ | _FUT2_ | _3.76E-07_ | _-0.18_ | _Chromatin_Structure\|\|DNase-seq\|Monocd14ro1746, Chromatin_Structure\|\|DNase-seq\|Cd20ro01794, Chromatin_Structure\|\|DNase-seq\|Heartoc, Chromatin_Structure\|Ifng4h\|FAIRE\|Helas3, Chromatin_Structure\|\|DNase-seq\|Cerebrumfrontaloc, Chromatin_Structure\|\|DNase-seq\|Gm12891, Chromatin_Structure\|\|DNase-seq\|Stellate, Chromatin_Structure\|\|FAIRE\|Gm12892, Chromatin_Structure\|\|DNase-seq\|H7es, Chromatin_Structure\|\|DNase-seq\|Monocd14, Chromatin_Structure\|\|DNase-seq\|H1hesc, Chromatin_Structure\|\|DNase-seq\|Adultcd4th0, Chromatin_Structure\|\|DNase-seq\|Adultcd4th1, Chromatin_Structure\|\|DNase-seq\|Cll, Chromatin_Structure\|\|DNase-seq\|Hepatocytes, Chromatin_Structure\|\|DNase-seq\|Cerebellumoc, Chromatin_Structure\|\|DNase-seq\|Gm10266, Chromatin_Structure\|\|DNase-seq\|Frontalcortexoc, Chromatin_Structure\|\|DNase-seq\|Huh7, Chromatin_Structure\|\|DNase-seq\|Gm19238, Chromatin_Structure\|\|DNase-seq\|Huh75, Chromatin_Structure\|\|DNase-seq\|Gm13977, Chromatin_Structure\|\|DNase-seq\|Th1, Chromatin_Structure\|\|DNase-seq\|Chorion, Chromatin_Structure\|\|DNase-seq\|Gm12892, Chromatin_Structure\|\|DNase-seq\|Psoasmuscleoc, Chromatin_Structure\|\|DNase-seq\|Gm06990, Protein_Binding\|forskolin\|ChIP-seq\|HepG2\|POLR2A, Protein_Binding\|\|ChIP-seq\|K562\|ZBTB7A_ | _4_ |  | _NA_ |  | _NA_ |
| _rs73068325_ | _C/T_ | _19_ | _59079096_ | _intron_variant_ | _NA_ | _NA_ | _NA_ | _NA_ | _No data_ | _7_ |  | _NA_ |  | _NA_ |
| _rs961253_ | _C/A_ | _20_ | _6404281_ |  | _ENSG00000125885_ | _MCM8_ | _0.0054_ | _-0.15_ | _Chromatin_Structure\|\|DNase-seq\|Hrpe, Chromatin_Structure\|\|FAIRE\|A549_ | _5_ |  | _NA_ |  | _NA_ |
| _rs994308_ | _C/T_ | _20_ | _6603622_ |  | _NA_ | _NA_ | _NA_ | _NA_ | _No data_ | _7_ |  | _NA_ |  | _NA_ |
| _rs6085661_ | _C/T_ | _20_ | _6693128_ |  | _ENSG00000088766_ | _CRLS1_ | _0.00711_ | _0.1_ | _Chromatin_Structure\|\|DNase-seq\|Hpaec, Chromatin_Structure\|\|DNase-seq\|Hmvecdneo, Chromatin_Structure\|\|DNase-seq\|Hpde6e6e7, Chromatin_Structure\|\|DNase-seq\|Hrpe, Chromatin_Structure\|\|DNase-seq\|Huvec, Chromatin_Structure\|\|DNase-seq\|Hcf, Chromatin_Structure\|Ifng4h\|FAIRE\|Helas3, Chromatin_Structure\|Est100nm1h\|DNase-seq\|Mcf7, Chromatin_Structure\|\|DNase-seq\|Rptec, Chromatin_Structure\|\|DNase-seq\|M059j, Chromatin_Structure\|\|DNase-seq\|Hcfaa, Chromatin_Structure\|\|DNase-seq\|Hmveclbl, Chromatin_Structure\|\|DNase-seq\|Hah, Chromatin_Structure\|\|DNase-seq\|Hrgec, Chromatin_Structure\|\|DNase-seq\|Hmvecdlyneo, Chromatin_Structure\|\|DNase-seq\|Hae, Chromatin_Structure\|Estctrl0h\|DNase-seq\|Mcf7, Chromatin_Structure\|\|DNase-seq\|Hmvecdblneo, Chromatin_Structure\|\|DNase-seq\|H7es, Chromatin_Structure\|\|DNase-seq\|Hasp, Chromatin_Structure\|\|DNase-seq\|Saec, Chromatin_Structure\|\|DNase-seq\|Hmvecdblad, Chromatin_Structure\|Diffa5d\|DNase-seq\|H7es, Chromatin_Structure\|\|FAIRE\|Helas3, Chromatin_Structure\|\|DNase-seq\|Hrce, Chromatin_Structure\|\|DNase-seq\|Mcf7, Chromatin_Structure\|\|DNase-seq\|Huh7, Chromatin_Structure\|\|DNase-seq\|Helas3, Chromatin_Structure\|\|DNase-seq\|Rwpe1, Chromatin_Structure\|\|DNase-seq\|Huh75, Chromatin_Structure\|\|DNase-seq\|Hee, Chromatin_Structure\|Diff4d\|DNase-seq\|Lhcnm2, Chromatin_Structure\|\|DNase-seq\|Hmveclly, Chromatin_Structure\|\|DNase-seq\|Lhcnm2, Chromatin_Structure\|\|DNase-seq\|Panc1, Chromatin_Structure\|\|DNase-seq\|Hs27a, Chromatin_Structure\|\|FAIRE\|Huvec, Chromatin_Structure\|\|DNase-seq\|Rpmi7951, Protein_Binding\|\|ChIP-seq\|HeLa-S3\|SMC3, Protein_Binding\|\|ChIP-seq\|HeLa-S3\|RCOR1, Protein_Binding\|\|ChIP-seq\|HUVEC\|GATA2, Protein_Binding\|\|ChIP-seq\|HeLa-S3\|JUN, Protein_Binding\|01pct\|ChIP-seq\|MCF10A-Er-Src\|FOS, Protein_Binding\|4ohtam_1um_12hr\|ChIP-seq\|MCF10A-Er-Src\|FOS, Protein_Binding\|\|ChIP-seq\|HeLa-S3\|USF2, Protein_Binding\|\|ChIP-seq\|HeLa-S3\|RAD21, Protein_Binding\|\|ChIP-seq\|HeLa-S3\|EP300, Protein_Binding\|\|ChIP-seq\|HeLa-S3\|CEBPB, Protein_Binding\|\|ChIP-seq\|HeLa-S3\|MXI1, Protein_Binding\|\|ChIP-seq\|HeLa-S3\|JUND, Protein_Binding\|\|ChIP-seq\|HUVEC\|FOS, Protein_Binding\|4ohtam_1um_4hr\|ChIP-seq\|MCF10A-Er-Src\|FOS, Protein_Binding\|4ohtam_1um_4hr\|ChIP-seq\|MCF10A-Er-Src\|MYC, Protein_Binding\|\|ChIP-seq\|HeLa-S3\|RFX5, Protein_Binding\|\|ChIP-seq\|HeLa-S3\|TCF7L2, Protein_Binding\|\|ChIP-seq\|HUVEC\|POLR2A_ | _4_ |  | _NA_ |  | _NA_ |
| _rs28488_ | _C/T_ | _20_ | _6762221_ |  | _NA_ | _NA_ | _NA_ | _NA_ | _Motifs\|PWM\|\|DMRT1_ | _6_ |  | _NA_ |  | _NA_ |
| _rs2423279_ | _T/C_ | _20_ | _7812350_ |  | _NA_ | _NA_ | _NA_ | _NA_ | _No data_ | _7_ |  | _NA_ |  | _NA_ |
| _rs2295444_ | _C/G/T_ | _20_ | _33173883_ | _intron_variant_ | _NA_ | _NA_ | _NA_ | _NA_ | _Motifs\|PWM\|\|Sry_ | _6_ |  | _NA_ |  | _NA_ |
| **_rs6065668*_** | **_C/T_** | **_20_** | **_42532821_** |  | **_NA_** | **_NA_** | **_NA_** | **_NA_** | **_Chromatin_Structure\|\|DNase-seq\|Colo829_** | **_5_** |  | **_NA_** |  | **_NA_** |
| _rs2179593_ | _C/A_ | _20_ | _42660286_ | _intron_variant_ | _ENSG00000124249_ | _KCNK15_ | _0.0131_ | _-0.17_ | _Chromatin_Structure\|\|DNase-seq\|Htr8, Chromatin_Structure\|\|DNase-seq\|Hae, Chromatin_Structure\|\|DNase-seq\|Tregwb78495824, Chromatin_Structure\|\|DNase-seq\|Rpmi7951_ | _5_ |  | _NA_ |  | _NA_ |
| _rs6066825_ | _A/G/T_ | _20_ | _47340117_ | _intron_variant_ | _ENSG00000124214_ | _STAU1_ | _0.0479_ | _-0.06_ | _Chromatin_Structure\|\|DNase-seq\|Progfib, Chromatin_Structure\|\|DNase-seq\|Nb4, Chromatin_Structure\|\|DNase-seq\|Monocd14ro1746, Chromatin_Structure\|\|DNase-seq\|Huvec, Chromatin_Structure\|Est100nm1h\|DNase-seq\|Mcf7, Chromatin_Structure\|Hypoxlaccon\|DNase-seq\|Mcf7, Chromatin_Structure\|\|DNase-seq\|Hpf, Chromatin_Structure\|\|DNase-seq\|Hvmf, Chromatin_Structure\|\|DNase-seq\|Hrgec, Chromatin_Structure\|\|DNase-seq\|Olfneurosphere, Chromatin_Structure\|Estctrl0h\|DNase-seq\|Mcf7, Chromatin_Structure\|\|DNase-seq\|H7es, Chromatin_Structure\|\|DNase-seq\|Hsmm, Chromatin_Structure\|\|DNase-seq\|Monocd14, Chromatin_Structure\|\|DNase-seq\|Gm13976, Chromatin_Structure\|\|DNase-seq\|Hbvp, Chromatin_Structure\|\|DNase-seq\|Hipe, Chromatin_Structure\|Diffa5d\|DNase-seq\|H7es, Chromatin_Structure\|\|DNase-seq\|Mcf7, Chromatin_Structure\|\|DNase-seq\|A549, Chromatin_Structure\|\|DNase-seq\|Huh7, Chromatin_Structure\|\|DNase-seq\|Hsmmt, Chromatin_Structure\|\|DNase-seq\|Hgf, Chromatin_Structure\|\|DNase-seq\|Hbvsmc, Chromatin_Structure\|\|DNase-seq\|Huh75, Chromatin_Structure\|\|DNase-seq\|Hre, Chromatin_Structure\|\|DNase-seq\|Nhdfneo, Chromatin_Structure\|\|DNase-seq\|Hepg2, Chromatin_Structure\|Hypoxlac\|DNase-seq\|Mcf7, Chromatin_Structure\|\|DNase-seq\|Gm12892, Chromatin_Structure\|\|DNase-seq\|Gm18507, Chromatin_Structure\|\|FAIRE\|Huvec, Protein_Binding\|\|ChIP-seq\|HepG2\|FOXA1, Protein_Binding\|serum_stimulated_media\|ChIP-seq\|MCF-7\|MYC, Protein_Binding\|dex_50nm\|ChIP-seq\|A549\|NR3C1, Protein_Binding\|vehicle\|ChIP-seq\|MCF-7\|MYC, Protein_Binding\|\|ChIP-seq\|HepG2\|FOXA2, Protein_Binding\|\|ChIP-seq\|HepG2\|NFIC, Protein_Binding\|serum_starved_media\|ChIP-seq\|MCF-7\|POLR2A, Protein_Binding\|\|ChIP-seq\|MCF-7\|GATA3, Protein_Binding\|\|ChIP-seq\|GM12878\|RUNX3, Protein_Binding\|\|ChIP-seq\|HepG2\|MYBL2_ | _4_ |  | _NA_ |  | _NA_ |
| _rs4811050_ | _G/A_ | _20_ | _48980670_ |  | _ENSG00000197818_ | _SLC9A8_ | _0.0116_ | _0.08_ | _Motifs\|PWM\|\|ER, Motifs\|PWM\|\|Gfi1b, Motifs\|Footprinting\|Htr8\|Gfi1b, Motifs\|Footprinting\|Mcf7\|Gfi1b, Motifs\|Footprinting\|Mcf7Hypoxlac\|Gfi1b, Chromatin_Structure\|\|FAIRE\|Htr8, Chromatin_Structure\|\|DNase-seq\|Gliobla_ | _5_ |  | _NA_ |  | _NA_ |
| _rs1810502_ | _C/T_ | _20_ | _49057488_ |  | _ENSG0000013550_ |  | _NA_ | _NA_ | _Single_Nucleotides\|KCNG1\|eQTL\|Liver, Single_Nucleotides\|BCAS4\|eQTL\|Liver, Single_Nucleotides\|CEBPB\|eQTL\|Liver, Motifs\|Footprinting\|Hmec\|Pax-8, Motifs\|Footprinting\|Hsmm\|Pax-8, Motifs\|Footprinting\|Huvec\|Pax-8, Motifs\|Footprinting\|Gliobla\|Pax-8, Motifs\|Footprinting\|Nhek\|Pax-8, Motifs\|Footprinting\|Chorion\|Pax-8, Motifs\|Footprinting\|Myometr\|Pax-8, Motifs\|PWM\|\|PAX5, Motifs\|Footprinting\|Huh75\|Pax-8, Motifs\|Footprinting\|LncapAndro\|Pax-8, Motifs\|Footprinting\|Phte\|Pax-8, Motifs\|Footprinting\|Gm12878\|Pax-8, Motifs\|Footprinting\|Panisd\|Pax-8, Motifs\|PWM\|\|Pax-8, Motifs\|Footprinting\|Helas3Ifna4h\|Pax-8, Motifs\|PWM\|\|PAX2, Motifs\|Footprinting\|Osteobl\|Pax-8, Motifs\|Footprinting\|Helas3\|Pax-8, Motifs\|Footprinting\|Htr8\|Pax-8, Motifs\|Footprinting\|8988t\|Pax-8, Motifs\|Footprinting\|Hepg2\|Pax-8, Motifs\|Footprinting\|A549\|Pax-8, Motifs\|Footprinting\|Mcf7\|Pax-8, Motifs\|Footprinting\|Mcf7Hypoxlac\|Pax-8, Motifs\|Footprinting\|K562\|Pax-8, Motifs\|Footprinting\|Fibrop\|Pax-8, Motifs\|Footprinting\|Gm12892\|Pax-8, Motifs\|Footprinting\|Hepatocytes\|Pax-8, Motifs\|Footprinting\|Hpde6e6e7\|Pax-8, Motifs\|Footprinting\|AosmcSerumfree\|Pax-8, Motifs\|Footprinting\|Fibrobl\|Pax-8, Motifs\|Footprinting\|Hsmmt\|Pax-8, Chromatin_Structure\|\|DNase-seq\|Progfib, Chromatin_Structure\|\|DNase-seq\|Nb4, Chromatin_Structure\|\|DNase-seq\|Htr8, Chromatin_Structure\|\|DNase-seq\|Hpaec, Chromatin_Structure\|Znfb34a8\|DNase-seq\|K562, Chromatin_Structure\|\|DNase-seq\|Hpdlf, Chromatin_Structure\|\|DNase-seq\|Hmvecdneo, Chromatin_Structure\|\|DNase-seq\|Fibropag08395, Chromatin_Structure\|\|DNase-seq\|Monocd14ro1746, Chromatin_Structure\|\|DNase-seq\|Hpde6e6e7, Chromatin_Structure\|\|DNase-seq\|Hrpe, Chromatin_Structure\|\|DNase-seq\|Huvec, Chromatin_Structure\|\|FAIRE\|UrotsaUt189, Chromatin_Structure\|Znfe103c6\|DNase-seq\|K562, Chromatin_Structure\|\|DNase-seq\|Hcf, Chromatin_Structure\|Ifng4h\|FAIRE\|Helas3, Chromatin_Structure\|\|DNase-seq\|Ag09319, Chromatin_Structure\|\|DNase-seq\|UrotsaUt189, Chromatin_Structure\|\|DNase-seq\|Panisd, Chromatin_Structure\|\|DNase-seq\|Prec, Chromatin_Structure\|\|DNase-seq\|Fibroblgm03348, Chromatin_Structure\|Est100nm1h\|DNase-seq\|Mcf7, Chromatin_Structure\|Znf4c50c4\|DNase-seq\|K562, Chromatin_Structure\|\|DNase-seq\|Ag10803, Chromatin_Structure\|\|DNase-seq\|Rptec, Chromatin_Structure\|Hypoxlaccon\|DNase-seq\|Mcf7, Chromatin_Structure\|\|DNase-seq\|Gm12864, Chromatin_Structure\|\|DNase-seq\|M059j, Chromatin_Structure\|\|DNase-seq\|Lncap, Chromatin_Structure\|\|FAIRE\|Htr8, Chromatin_Structure\|\|DNase-seq\|K562G2mphase, Chromatin_Structure\|\|DNase-seq\|Cd34mobilized, Chromatin_Structure\|Nabut\|FAIRE\|K562, Chromatin_Structure\|\|DNase-seq\|Gm04504, Chromatin_Structure\|\|DNase-seq\|Hmvecdad, Chromatin_Structure\|\|FAIRE\|Nhek, Chromatin_Structure\|\|DNase-seq\|Hcfaa, Chromatin_Structure\|\|DNase-seq\|8988t, Chromatin_Structure\|\|DNase-seq\|Hmveclbl, Chromatin_Structure\|\|DNase-seq\|Hah, Chromatin_Structure\|\|DNase-seq\|Wi38, Chromatin_Structure\|Ohurea\|FAIRE\|K562, Chromatin_Structure\|\|DNase-seq\|Hvmf, Chromatin_Structure\|\|DNase-seq\|Hrgec, Chromatin_Structure\|\|DNase-seq\|Olfneurosphere, Chromatin_Structure\|\|DNase-seq\|Hffmyc, Chromatin_Structure\|\|DNase-seq\|Stellate, Chromatin_Structure\|Ctcfshrna\|DNase-seq\|Mcf7, Chromatin_Structure\|\|DNase-seq\|Hmvecdlyneo, Chromatin_Structure\|\|DNase-seq\|Hae, Chromatin_Structure\|\|DNase-seq\|Th2, Chromatin_Structure\|Estctrl0h\|DNase-seq\|Mcf7, Chromatin_Structure\|Serumfree\|DNase-seq\|Aosmc, Chromatin_Structure\|\|DNase-seq\|Ag04450, Chromatin_Structure\|\|DNase-seq\|Hct116, Chromatin_Structure\|\|DNase-seq\|H7es, Chromatin_Structure\|\|DNase-seq\|Hmvecdblneo, Chromatin_Structure\|\|DNase-seq\|Hsmm, Chromatin_Structure\|\|DNase-seq\|Fibropag08396, Chromatin_Structure\|\|DNase-seq\|Hac, Chromatin_Structure\|\|DNase-seq\|Monocd14, Chromatin_Structure\|\|DNase-seq\|Ag09309, Chromatin_Structure\|\|DNase-seq\|Hpaf, Chromatin_Structure\|\|DNase-seq\|Skmc, Chromatin_Structure\|\|DNase-seq\|Hasp, Chromatin_Structure\|\|DNase-seq\|Th2wb54553204, Chromatin_Structure\|\|DNase-seq\|Panislets, Chromatin_Structure\|\|FAIRE\|Gliobla, Chromatin_Structure\|Znfa41c6\|DNase-seq\|K562, Chromatin_Structure\|Znff41b2\|DNase-seq\|K562, Chromatin_Structure\|\|DNase-seq\|Saec, Chromatin_Structure\|\|DNase-seq\|H1hesc, Chromatin_Structure\|Lenticon\|DNase-seq\|Fibroblgm03348, Chromatin_Structure\|Ifna4h\|FAIRE\|Helas3, Chromatin_Structure\|\|DNase-seq\|Adultcd4th0, Chromatin_Structure\|\|DNase-seq\|Fibrobl, Chromatin_Structure\|\|DNase-seq\|Hmvecdblad, Chromatin_Structure\|\|DNase-seq\|Nhbera, Chromatin_Structure\|\|DNase-seq\|Hipe, Chromatin_Structure\|Diffa5d\|DNase-seq\|H7es, Chromatin_Structure\|\|DNase-seq\|K562, Chromatin_Structure\|\|DNase-seq\|Hff, Chromatin_Structure\|\|DNase-seq\|Th1wb54553204, Chromatin_Structure\|\|DNase-seq\|Adultcd4th1, Chromatin_Structure\|\|DNase-seq\|Mel2183, Chromatin_Structure\|\|FAIRE\|Helas3, Chromatin_Structure\|\|FAIRE\|Urotsa, Chromatin_Structure\|\|DNase-seq\|Hnpce, Chromatin_Structure\|\|DNase-seq\|Hrce, Chromatin_Structure\|Saha1u72hr\|DNase-seq\|K562, Chromatin_Structure\|\|DNase-seq\|Hl60, Chromatin_Structure\|\|DNase-seq\|Mcf7, Chromatin_Structure\|\|DNase-seq\|Colo829, Chromatin_Structure\|\|DNase-seq\|Fibrop, Chromatin_Structure\|\|DNase-seq\|Cmk, Chromatin_Structure\|\|DNase-seq\|Nhek, Chromatin_Structure\|\|DNase-seq\|Gliobla, Chromatin_Structure\|\|DNase-seq\|T47d, Chromatin_Structure\|\|DNase-seq\|A549, Chromatin_Structure\|\|DNase-seq\|Huh7, Chromatin_Structure\|\|DNase-seq\|Caco2, Chromatin_Structure\|Sahactrl\|DNase-seq\|K562, Chromatin_Structure\|\|DNase-seq\|Helas3, Chromatin_Structure\|Nabut\|DNase-seq\|K562, Chromatin_Structure\|\|DNase-seq\|Hgf, Chromatin_Structure\|\|DNase-seq\|Hsmmt, Chromatin_Structure\|\|DNase-seq\|Hbvsmc, Chromatin_Structure\|Ifna4h\|DNase-seq\|Helas3, Chromatin_Structure\|Znfp5\|DNase-seq\|K562, Chromatin_Structure\|\|DNase-seq\|Nha, Chromatin_Structure\|\|DNase-seq\|Urotsa, Chromatin_Structure\|\|DNase-seq\|Rwpe1, Chromatin_Structure\|\|DNase-seq\|Huh75, Chromatin_Structure\|\|DNase-seq\|Gm04503, Chromatin_Structure\|\|DNase-seq\|Hconf, Chromatin_Structure\|\|DNase-seq\|Hre, Chromatin_Structure\|\|DNase-seq\|Hmec, Chromatin_Structure\|\|DNase-seq\|Nhdfneo, Chromatin_Structure\|\|FAIRE\|K562, Chromatin_Structure\|\|DNase-seq\|Osteobl, Chromatin_Structure\|\|DNase-seq\|Phte, Chromatin_Structure\|\|DNase-seq\|Hcm, Chromatin_Structure\|\|DNase-seq\|Hmvecdlyad, Chromatin_Structure\|\|DNase-seq\|Hee, Chromatin_Structure\|Ohtam\|DNase-seq\|Wi38, Chromatin_Structure\|Diff4d\|DNase-seq\|Lhcnm2, Chromatin_Structure\|\|DNase-seq\|Imr90, Chromatin_Structure\|Lentimyod\|DNase-seq\|Fibroblgm03348, Chromatin_Structure\|\|DNase-seq\|Hepg2, Chromatin_Structure\|\|DNase-seq\|Hmveclly, Chromatin_Structure\|Hypoxlac\|DNase-seq\|Mcf7, Chromatin_Structure\|\|DNase-seq\|Bj, Chromatin_Structure\|Hypoxlac\|FAIRE\|Mcf7, Chromatin_Structure\|\|DNase-seq\|Lhcnm2, Chromatin_Structure\|\|DNase-seq\|Ag04449, Chromatin_Structure\|\|DNase-seq\|Hsmmfshd, Chromatin_Structure\|\|DNase-seq\|Fibropag20443, Chromatin_Structure\|\|DNase-seq\|Hsmmemb, Chromatin_Structure\|\|DNase-seq\|Nhlf, Chromatin_Structure\|\|DNase-seq\|Th1, Chromatin_Structure\|\|DNase-seq\|K562G1phase, Chromatin_Structure\|\|DNase-seq\|Chorion, Chromatin_Structure\|\|DNase-seq\|Myometr, Chromatin_Structure\|Znfg54a11\|DNase-seq\|K562, Chromatin_Structure\|\|DNase-seq\|Gm12892, Chromatin_Structure\|\|DNase-seq\|Hs27a, Chromatin_Structure\|\|DNase-seq\|Gm12878, Chromatin_Structure\|\|DNase-seq\|Psoasmuscleoc, Chromatin_Structure\|Randshrna\|DNase-seq\|Mcf7, Chromatin_Structure\|Znf2c10c5\|DNase-seq\|K562, Chromatin_Structure\|\|FAIRE\|Huvec, Chromatin_Structure\|\|DNase-seq\|Msc, Chromatin_Structure\|Znf4g7d3\|DNase-seq\|K562, Chromatin_Structure\|\|FAIRE\|Hepg2, Chromatin_Structure\|\|DNase-seq\|Nhdfad, Chromatin_Structure\|\|DNase-seq\|Be2c, Chromatin_Structure\|\|DNase-seq\|Aoaf, Chromatin_Structure\|\|DNase-seq\|Rpmi7951, Protein_Binding\|\|ChIP-seq\|MCF-7\|ZNF217, Protein_Binding\|\|ChIP-seq\|SK-N-SH\|EP300, Protein_Binding\|02pct\|ChIP-seq\|A549\|ELF1, Protein_Binding\|\|ChIP-seq\|HepG2\|FOSL2, Protein_Binding\|02pct\|ChIP-seq\|A549\|BCL3, Protein_Binding\|\|ChIP-seq\|GM12878\|BATF, Protein_Binding\|\|ChIP-seq\|HeLa-S3\|SMC3, Protein_Binding\|\|ChIP-seq\|HepG2\|JUND, Protein_Binding\|02pct\|ChIP-seq\|A549\|SIX5, Protein_Binding\|\|ChIP-seq\|HepG2\|BHLHE40, Protein_Binding\|\|ChIP-seq\|HepG2\|MBD4, Protein_Binding\|\|ChIP-seq\|HeLa-S3\|RCOR1, Protein_Binding\|\|ChIP-seq\|HeLa-S3\|CHD2, Protein_Binding\|\|ChIP-seq\|HepG2\|MXI1, Protein_Binding\|\|ChIP-seq\|HUVEC\|GATA2, Protein_Binding\|\|ChIP-seq\|HepG2\|FOXA1, Protein_Binding\|\|ChIP-seq\|HUVEC\|JUN, Protein_Binding\|\|ChIP-seq\|K562\|JUND, Protein_Binding\|01pct\|ChIP-seq\|MCF10A-Er-Src\|MYC, Protein_Binding\|\|ChIP-seq\|HeLa-S3\|JUN, Protein_Binding\|\|ChIP-seq\|K562\|MYC, Protein_Binding\|\|ChIP-seq\|K562\|ATF1, Protein_Binding\|serum_stimulated_media\|ChIP-seq\|MCF-7\|MYC, Protein_Binding\|01pct\|ChIP-seq\|MCF10A-Er-Src\|FOS, Protein_Binding\|4ohtam_1um_12hr\|ChIP-seq\|MCF10A-Er-Src\|FOS, Protein_Binding\|\|ChIP-seq\|HepG2\|HDAC2, Protein_Binding\|shbrg1\|ChIP-seq\|CD36\|GATA1, Protein_Binding\|dex_50nm\|ChIP-seq\|A549\|NR3C1, Protein_Binding\|vehicle\|ChIP-seq\|MCF-7\|MYC, Protein_Binding\|\|ChIP-seq\|HeLa-S3\|ZNF143, Protein_Binding\|\|ChIP-seq\|HeLa-S3\|TFAP2C, Protein_Binding\|\|ChIP-seq\|U87\|POLR2A, Protein_Binding\|ifna6h\|ChIP-seq\|K562\|MYC, Protein_Binding\|\|ChIP-seq\|HepG2\|SIN3A, Protein_Binding\|ifng6h\|ChIP-seq\|K562\|JUN, Protein_Binding\|\|ChIP-seq\|HepG2\|EP300, Protein_Binding\|\|ChIP-seq\|HepG2\|RCOR1, Protein_Binding\|\|ChIP-seq\|HepG2\|RAD21, Protein_Binding\|\|ChIP-seq\|HepG2\|JUN, Protein_Binding\|02pct\|ChIP-seq\|A549\|GABPB1, Protein_Binding\|ifng6h\|ChIP-seq\|K562\|MYC, Protein_Binding\|\|ChIP-seq\|GM12878\|BCL11A, Protein_Binding\|\|ChIP-seq\|HeLa-S3\|STAT3, Protein_Binding\|\|ChIP-seq\|HeLa-S3\|MAX, Protein_Binding\|dex_100nm\|ChIP-seq\|A549\|NR3C1, Protein_Binding\|\|ChIP-seq\|K562\|TBL1XR1, Protein_Binding\|\|ChIP-seq\|K562\|TRIM28, Protein_Binding\|\|ChIP-seq\|K562\|GABPB1, Protein_Binding\|\|ChIP-seq\|HeLa-S3\|RAD21, Protein_Binding\|\|ChIP-seq\|A549\|CEBPB, Protein_Binding\|4ohtam_1um_12hr\|ChIP-seq\|MCF10A-Er-Src\|STAT3, Protein_Binding\|\|ChIP-seq\|PFSK-1\|REST, Protein_Binding\|02pct\|ChIP-seq\|A549\|SIN3A, Protein_Binding\|\|ChIP-seq\|HepG2\|REST, Protein_Binding\|\|ChIP-seq\|HeLa-S3\|TFAP2A, Protein_Binding\|\|ChIP-seq\|K562\|JUN, Protein_Binding\|\|ChIP-seq\|K562\|JUNB, Protein_Binding\|\|ChIP-seq\|K562\|ELF1, Protein_Binding\|\|ChIP-seq\|K562\|POLR2A, Protein_Binding\|ifna6h\|ChIP-seq\|K562\|JUN, Protein_Binding\|ifna30\|ChIP-seq\|K562\|JUN, Protein_Binding\|differential\|ChIP-seq\|Caco2\|CDX2, Protein_Binding\|\|ChIP-seq\|HepG2\|NFIC, Protein_Binding\|4ohtam_1um_36hr\|ChIP-seq\|MCF10A-Er-Src\|FOS, Protein_Binding\|\|ChIP-seq\|MCF-7\|E2F1, Protein_Binding\|estrogen\|ChIP-seq\|MCF-7\|MYC, Protein_Binding\|\|ChIP-seq\|K562\|FOSL1, Protein_Binding\|\|ChIP-seq\|HeLa-S3\|MAFK, Protein_Binding\|\|ChIP-seq\|HeLa-S3\|EP300, Protein_Binding\|\|ChIP-seq\|HeLa-S3\|CEBPB, Protein_Binding\|\|ChIP-seq\|K562\|UBTF, Protein_Binding\|02pct\|ChIP-seq\|A549\|TCF12, Protein_Binding\|01pct_12hr\|ChIP-seq\|MCF10A-Er-Src\|STAT3, Protein_Binding\|dex_500pm\|ChIP-seq\|A549\|NR3C1, Protein_Binding\|\|ChIP-seq\|HepG2\|YY1, Protein_Binding\|\|ChIP-seq\|HeLa-S3\|ELK4, Protein_Binding\|\|ChIP-seq\|K562\|MAX, Protein_Binding\|\|ChIP-seq\|K562\|RCOR1, Protein_Binding\|\|ChIP-seq\|HepG2\|CEBPD, Protein_Binding\|shLuc\|ChIP-seq\|CD36\|GATA1, Protein_Binding\|forskolin\|ChIP-seq\|HepG2\|HNF4A, Protein_Binding\|\|ChIP-seq\|HCT-116\|POLR2A, Protein_Binding\|02pct\|ChIP-seq\|A549\|YY1, Protein_Binding\|\|ChIP-seq\|HeLa-S3\|MXI1, Protein_Binding\|\|ChIP-seq\|K562\|HDAC2, Protein_Binding\|\|ChIP-seq\|HepG2\|ARID3A, Protein_Binding\|\|ChIP-seq\|K562\|BHLHE40, Protein_Binding\|02pct\|ChIP-seq\|A549\|REST, Protein_Binding\|\|ChIP-seq\|MCF-7\|GATA3, Protein_Binding\|\|ChIP-seq\|K562\|SRF, Protein_Binding\|\|ChIP-seq\|HepG2\|CEBPB, Protein_Binding\|\|ChIP-seq\|HeLa-S3\|JUND, Protein_Binding\|\|ChIP-seq\|K562\|GATA2, Protein_Binding\|\|ChIP-seq\|IMR90\|POLR2A, Protein_Binding\|01pct\|ChIP-seq\|MCF10A-Er-Src\|STAT3, Protein_Binding\|02pct\|ChIP-seq\|A549\|FOSL2, Protein_Binding\|\|ChIP-seq\|K562\|CBX3, Protein_Binding\|forskolin\|ChIP-seq\|HepG2\|ESRRA, Protein_Binding\|\|ChIP-seq\|K562\|PML, Protein_Binding\|4ohtam_1um_36hr\|ChIP-seq\|MCF10A-Er-Src\|STAT3, Protein_Binding\|02pct\|ChIP-seq\|A549\|EP300, Protein_Binding\|\|ChIP-seq\|SK-N-SH\|POLR2A, Protein_Binding\|\|ChIP-seq\|GM12878\|RUNX3, Protein_Binding\|02pct\|ChIP-seq\|A549\|ZBTB33, Protein_Binding\|dex_100nm\|ChIP-seq\|A549\|CREB1, Protein_Binding\|ifna30\|ChIP-seq\|K562\|MYC, Protein_Binding\|\|ChIP-seq\|HCT-116\|TCF7L2, Protein_Binding\|\|ChIP-seq\|HUVEC\|FOS, Protein_Binding\|\|ChIP-seq\|HepG2\|MYC, Protein_Binding\|4ohtam_1um_4hr\|ChIP-seq\|MCF10A-Er-Src\|FOS, Protein_Binding\|\|ChIP-seq\|HeLa-S3\|FOS, Protein_Binding\|\|ChIP-seq\|HepG2\|HNF4G, Protein_Binding\|\|ChIP-seq\|K562\|BCLAF1, Protein_Binding\|\|ChIP-seq\|GM12878\|PAX5, Protein_Binding\|\|ChIP-seq\|HepG2\|SMC3, Protein_Binding\|differential\|ChIP-seq\|Caco2\|HNF4A, Protein_Binding\|01pct\|ChIP-seq\|MCF10A-Er-Src\|POLR2A, Protein_Binding\|\|ChIP-seq\|HepG2\|TCF12, Protein_Binding\|\|ChIP-seq\|K562\|EP300, Protein_Binding\|ifng30\|ChIP-seq\|K562\|JUN, Protein_Binding\|4ohtam_1um_4hr\|ChIP-seq\|MCF10A-Er-Src\|MYC, Protein_Binding\|forskolin\|ChIP-seq\|HepG2\|CEBPB, Protein_Binding\|\|ChIP-seq\|HeLa-S3\|RFX5, Protein_Binding\|\|ChIP-seq\|HepG2\|MYBL2, Protein_Binding\|\|ChIP-seq\|K562\|TAL1, Protein_Binding\|\|ChIP-seq\|K562\|STAT5A, Protein_Binding\|\|ChIP-seq\|HepG2\|POLR2A, Protein_Binding\|\|ChIP-seq\|HeLa-S3\|TBP, Protein_Binding\|\|ChIP-seq\|HeLa-S3\|TCF7L2, Protein_Binding\|01pct_4hr\|ChIP-seq\|MCF10A-Er-Src\|STAT3, Protein_Binding\|\|ChIP-seq\|K562\|ZNF143, Protein_Binding\|\|ChIP-seq\|HUVEC\|POLR2A, Protein_Binding\|\|ChIP-seq\|K562\|FOS, Protein_Binding\|dex_5nm\|ChIP-seq\|A549\|NR3C1, Protein_Binding\|ifng30\|ChIP-seq\|K562\|MYC, Protein_Binding\|\|ChIP-seq\|K562\|MAFF, Protein_Binding\|\|ChIP-seq\|HeLa-S3\|GABPB1, Protein_Binding\|\|ChIP-seq\|K562\|ATF3, Protein_Binding\|02pct\|ChIP-seq\|A549\|ATF3, Protein_Binding\|\|ChIP-seq\|NHEK\|POLR2A_ | _1b_ |  | _NA_ |  | _NA_ |
| _rs6091213_ | _T/A/C_ | _20_ | _49384745_ |  | _ENSG00000259456_ | _RP5-914P20.5_ | _0.0129_ | _0.19_ | _Chromatin_Structure\|Est100nm1h\|DNase-seq\|Mcf7, Chromatin_Structure\|Estctrl0h\|DNase-seq\|Mcf7, Chromatin_Structure\|\|DNase-seq\|H7es, Chromatin_Structure\|\|DNase-seq\|K562, Chromatin_Structure\|Saha1u72hr\|DNase-seq\|K562, Chromatin_Structure\|\|DNase-seq\|Mcf7, Chromatin_Structure\|\|DNase-seq\|Huh7, Chromatin_Structure\|\|DNase-seq\|Huh75, Protein_Binding\|\|ChIP-seq\|H1-hESC\|CTCF, Protein_Binding\|\|ChIP-seq\|K562\|NR2F2_ | _4_ |  | _NA_ |  | _NA_ |
| _rs1741640_ | _T/C_ | _20_ | _60932414_ | _intron_variant_ | _ENSG00000149679_ | _CABLES2_ | _6.38E-05_ | _-0.25_ | _Chromatin_Structure\|\|DNase-seq\|Progfib, Chromatin_Structure\|\|DNase-seq\|Htr8, Chromatin_Structure\|\|DNase-seq\|Hpaec, Chromatin_Structure\|\|DNase-seq\|Fibropag08395, Chromatin_Structure\|\|DNase-seq\|Hpde6e6e7, Chromatin_Structure\|\|DNase-seq\|Huvec, Chromatin_Structure\|\|FAIRE\|UrotsaUt189, Chromatin_Structure\|Znfe103c6\|DNase-seq\|K562, Chromatin_Structure\|Est10nm30m\|DNase-seq\|Ishikawa, Chromatin_Structure\|\|DNase-seq\|Cd20ro01794, Chromatin_Structure\|\|DNase-seq\|Heartoc, Chromatin_Structure\|\|DNase-seq\|Hcf, Chromatin_Structure\|Ifng4h\|FAIRE\|Helas3, Chromatin_Structure\|\|DNase-seq\|UrotsaUt189, Chromatin_Structure\|\|DNase-seq\|Panisd, Chromatin_Structure\|\|DNase-seq\|Fibroblgm03348, Chromatin_Structure\|\|DNase-seq\|Sknsh, Chromatin_Structure\|Hypoxlaccon\|DNase-seq\|Mcf7, Chromatin_Structure\|\|FAIRE\|Htr8, Chromatin_Structure\|\|DNase-seq\|Lncap, Chromatin_Structure\|\|DNase-seq\|K562G2mphase, Chromatin_Structure\|\|DNase-seq\|Cerebrumfrontaloc, Chromatin_Structure\|\|DNase-seq\|Hmvecdad, Chromatin_Structure\|\|DNase-seq\|Gm12891, Chromatin_Structure\|\|DNase-seq\|8988t, Chromatin_Structure\|\|DNase-seq\|Hpf, Chromatin_Structure\|\|DNase-seq\|Gm19240, Chromatin_Structure\|\|DNase-seq\|Melano, Chromatin_Structure\|ra\|DNase-seq\|Sknsh, Chromatin_Structure\|\|DNase-seq\|Olfneurosphere, Chromatin_Structure\|Ctcfshrna\|DNase-seq\|Mcf7, Chromatin_Structure\|\|DNase-seq\|Stellate, Chromatin_Structure\|\|DNase-seq\|Medullod341, Chromatin_Structure\|Serumfree\|DNase-seq\|Aosmc, Chromatin_Structure\|\|DNase-seq\|Gm20000, Chromatin_Structure\|\|DNase-seq\|H7es, Chromatin_Structure\|\|DNase-seq\|Fibropag08396, Chromatin_Structure\|\|DNase-seq\|Hsmm, Chromatin_Structure\|\|DNase-seq\|Monocd14, Chromatin_Structure\|\|DNase-seq\|Panislets, Chromatin_Structure\|\|DNase-seq\|Gm13976, Chromatin_Structure\|\|DNase-seq\|H1hesc, Chromatin_Structure\|\|DNase-seq\|Hbvp, Chromatin_Structure\|Lenticon\|DNase-seq\|Fibroblgm03348, Chromatin_Structure\|Ifna4h\|FAIRE\|Helas3, Chromatin_Structure\|\|DNase-seq\|Fibrobl, Chromatin_Structure\|\|DNase-seq\|Hmvecdblad, Chromatin_Structure\|\|DNase-seq\|K562, Chromatin_Structure\|Tam10030\|DNase-seq\|Ishikawa, Chromatin_Structure\|Andro\|DNase-seq\|Lncap, Chromatin_Structure\|\|FAIRE\|Urotsa, Chromatin_Structure\|Saha1u72hr\|DNase-seq\|K562, Chromatin_Structure\|\|DNase-seq\|Ips, Chromatin_Structure\|\|DNase-seq\|Mcf7, Chromatin_Structure\|\|DNase-seq\|Fibrop, Chromatin_Structure\|\|DNase-seq\|Hepatocytes, Chromatin_Structure\|\|DNase-seq\|Cerebellumoc, Chromatin_Structure\|\|DNase-seq\|Nhek, Chromatin_Structure\|\|DNase-seq\|Frontalcortexoc, Chromatin_Structure\|\|DNase-seq\|Gliobla, Chromatin_Structure\|\|DNase-seq\|T47d, Chromatin_Structure\|\|DNase-seq\|A549, Chromatin_Structure\|\|DNase-seq\|Huh7, Chromatin_Structure\|Sahactrl\|DNase-seq\|K562, Chromatin_Structure\|\|DNase-seq\|Helas3, Chromatin_Structure\|Nabut\|DNase-seq\|K562, Chromatin_Structure\|\|DNase-seq\|Hgf, Chromatin_Structure\|\|DNase-seq\|Hsmmt, Chromatin_Structure\|\|DNase-seq\|Gm19238, Chromatin_Structure\|Ifna4h\|DNase-seq\|Helas3, Chromatin_Structure\|\|DNase-seq\|Urotsa, Chromatin_Structure\|Est10nm30m\|DNase-seq\|T47d, Chromatin_Structure\|\|DNase-seq\|Gm19239, Chromatin_Structure\|\|DNase-seq\|Rwpe1, Chromatin_Structure\|\|DNase-seq\|Huh75, Chromatin_Structure\|\|DNase-seq\|Hmec, Chromatin_Structure\|\|FAIRE\|K562, Chromatin_Structure\|\|DNase-seq\|Osteobl, Chromatin_Structure\|Est10nm30m\|DNase-seq\|Ecc1, Chromatin_Structure\|\|DNase-seq\|Phte, Chromatin_Structure\|\|DNase-seq\|Gm13977, Chromatin_Structure\|\|DNase-seq\|Imr90, Chromatin_Structure\|\|DNase-seq\|Hmveclly, Chromatin_Structure\|\|DNase-seq\|Hepg2, Chromatin_Structure\|Lentimyod\|DNase-seq\|Fibroblgm03348, Chromatin_Structure\|Hypoxlac\|DNase-seq\|Mcf7, Chromatin_Structure\|\|DNase-seq\|Hsmmfshd, Chromatin_Structure\|\|DNase-seq\|Fibropag20443, Chromatin_Structure\|\|DNase-seq\|Hsmmemb, Chromatin_Structure\|\|DNase-seq\|K562G1phase, Chromatin_Structure\|\|DNase-seq\|Medullo, Chromatin_Structure\|\|DNase-seq\|Chorion, Chromatin_Structure\|\|DNase-seq\|Myometr, Chromatin_Structure\|Znfg54a11\|DNase-seq\|K562, Chromatin_Structure\|Dm002p1h\|DNase-seq\|Ecc1, Chromatin_Structure\|\|DNase-seq\|Gm12892, Chromatin_Structure\|\|DNase-seq\|Hs27a, Chromatin_Structure\|\|DNase-seq\|Psoasmuscleoc, Chromatin_Structure\|Randshrna\|DNase-seq\|Mcf7, Chromatin_Structure\|\|FAIRE\|Huvec, Chromatin_Structure\|\|DNase-seq\|Be2c, Chromatin_Structure\|\|DNase-seq\|Aoaf, Chromatin_Structure\|\|DNase-seq\|Rpmi7951, Protein_Binding\|\|ChIP-seq\|PBDE\|GATA1, Protein_Binding\|\|ChIP-seq\|SK-N-SH\|EP300, Protein_Binding\|\|ChIP-seq\|K562\|ZBTB7A, Protein_Binding\|dex_100nm\|ChIP-seq\|A549\|POLR2A, Protein_Binding\|\|ChIP-seq\|HUVEC\|GATA2, Protein_Binding\|\|ChIP-seq\|K562\|JUND, Protein_Binding\|dex_100nm\|ChIP-seq\|ECC-1\|NR3C1, Protein_Binding\|\|ChIP-seq\|K562\|CCNT2, Protein_Binding\|4ohtam_1um_12hr\|ChIP-seq\|MCF10A-Er-Src\|FOS, Protein_Binding\|dex_50nm\|ChIP-seq\|A549\|NR3C1, Protein_Binding\|shbrg1\|ChIP-seq\|CD36\|GATA1, Protein_Binding\|\|ChIP-seq\|HeLa-S3\|MAZ, Protein_Binding\|\|ChIP-seq\|K562\|TBP, Protein_Binding\|dex_100nm\|ChIP-seq\|A549\|NR3C1, Protein_Binding\|\|ChIP-seq\|K562\|TEAD4, Protein_Binding\|\|ChIP-seq\|K562\|TRIM28, Protein_Binding\|\|ChIP-seq\|K562\|GABPB1, Protein_Binding\|\|ChIP-seq\|K562\|POLR2A, Protein_Binding\|4ohtam_1um_36hr\|ChIP-seq\|MCF10A-Er-Src\|FOS, Protein_Binding\|\|ChIP-seq\|HeLa-S3\|GTF2F1, Protein_Binding\|\|ChIP-seq\|K562\|MAX, Protein_Binding\|\|ChIP-seq\|K562\|YY1, Protein_Binding\|shLuc\|ChIP-seq\|CD36\|GATA1, Protein_Binding\|\|ChIP-seq\|HCT-116\|POLR2A, Protein_Binding\|\|ChIP-seq\|K562\|GATA2, Protein_Binding\|\|ChIP-seq\|K562\|ETS1, Protein_Binding\|02pct\|ChIP-seq\|A549\|FOSL2, Protein_Binding\|\|ChIP-seq\|K562\|MAZ, Protein_Binding\|\|ChIP-seq\|HCT-116\|TCF7L2, Protein_Binding\|\|ChIP-seq\|HUVEC\|FOS, Protein_Binding\|\|ChIP-seq\|K562\|NR2F2, Protein_Binding\|\|ChIP-seq\|K562\|EP300, Protein_Binding\|\|ChIP-seq\|K562\|TAL1, Protein_Binding\|\|ChIP-seq\|K562\|STAT5A, Protein_Binding\|\|ChIP-seq\|K562\|GATA1, Protein_Binding\|\|ChIP-seq\|K562\|HMGN3, Protein_Binding\|\|ChIP-seq\|HUVEC\|POLR2A, Protein_Binding\|ifng30\|ChIP-seq\|K562\|MYC_ | _4_ |  | _NA_ |  | _NA_ |
| _rs3787089_ | _C/T_ | _20_ | _62316630_ | _intron_variant_ | _ENSG00000203896.5_ | _LIME1_ | _3.27E-06_ | _-0.16_ | _Chromatin_Structure\|\|DNase-seq\|Cd20ro01794, Chromatin_Structure\|\|DNase-seq\|Heartoc, Chromatin_Structure\|\|DNase-seq\|Lncap, Chromatin_Structure\|\|DNase-seq\|Gm12891, Chromatin_Structure\|\|DNase-seq\|8988t, Chromatin_Structure\|\|DNase-seq\|Gm19240, Chromatin_Structure\|\|DNase-seq\|Medullod341, Chromatin_Structure\|\|DNase-seq\|H7es, Chromatin_Structure\|\|DNase-seq\|H1hesc, Chromatin_Structure\|Ifna4h\|FAIRE\|Helas3, Chromatin_Structure\|\|DNase-seq\|Adultcd4th0, Chromatin_Structure\|\|DNase-seq\|Adultcd4th1, Chromatin_Structure\|\|DNase-seq\|Ips, Chromatin_Structure\|\|DNase-seq\|Hepatocytes, Chromatin_Structure\|\|DNase-seq\|Frontalcortexoc, Chromatin_Structure\|\|DNase-seq\|Huh7, Chromatin_Structure\|\|DNase-seq\|Gm19238, Chromatin_Structure\|\|DNase-seq\|Huh75, Chromatin_Structure\|\|DNase-seq\|Hmec, Chromatin_Structure\|\|DNase-seq\|Osteobl, Chromatin_Structure\|\|DNase-seq\|Hepg2, Chromatin_Structure\|\|DNase-seq\|Medullo, Chromatin_Structure\|\|DNase-seq\|Gm12892, Protein_Binding\|\|ChIP-seq\|HeLa-S3\|POLR2A_ | _4_ |  | _NA_ |  | _NA_ |
| _rs5934683_ | _T/C_ | _X_ | _9751474_ | _intron_variant_ | _NA_ | _NA_ | _NA_ | _NA_ | _Chromatin_Structure\|\|DNase-seq\|Gm19240, Chromatin_Structure\|\|DNase-seq\|Hsmm, Chromatin_Structure\|\|DNase-seq\|Mcf7, Chromatin_Structure\|\|DNase-seq\|Hepatocytes, Chromatin_Structure\|\|DNase-seq\|Hsmmt, Chromatin_Structure\|\|DNase-seq\|Osteobl, Chromatin_Structure\|\|DNase-seq\|H9es, Chromatin_Structure\|\|DNase-seq\|Myometr, Chromatin_Structure\|\|DNase-seq\|Psoasmuscleoc_ | _5_ |  | _NA_ |  | _NA_ |
| _rs2732875_ | _C/G/T_ | _X_ | _9763898_ | _intron_variant_ | _ENSG00000146950.8_ | _SHROOM2_ | _1.16E-19_ | _0.51_ | _No data_ | _7_ |  | _NA_ |  | _NA_ |

*Variants associated with colorectal cancer survival outcomes at p<0.05
